# Synthesis of Dolutegravir Exploiting Continuous Flow Chemistry

**DOI:** 10.1021/acs.joc.3c01365

**Published:** 2023-08-08

**Authors:** Sinazo Nqeketo, Paul Watts

**Affiliations:** Nelson Mandela University, University Way,Port Elizabeth6031, South Africa

## Abstract

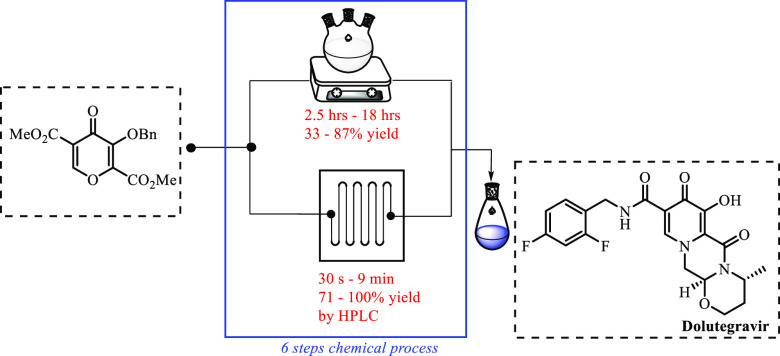

An efficient continuous flow process for the synthesis of dolutegravir, an active pharmaceutical ingredient (API) for HIV treatment, was investigated. The synthetic procedure starts from a readily available benzyl-protected pyran via six chemical transformations using continuous flow reactors. The significant advantage of this flow process includes the reduction of the overall reaction time from 34.5 h in batch to 14.5 min. The overall yield of each reaction step improved dramatically upon flow optimization. Another key feature of this synthesis is telescoping multiple steps.

## Introduction

Human immunodeficiency virus (HIV) remains a critical global public health challenge, and to date, there are around 38 million people living with HIV in the world.^1^ A lot of antiretroviral drugs to combat this epidemic have been approved by the US Food and Drug Administration (FDA). HIV integrase inhibitors (INIs) are the most recently approved class of drugs that interfere with the HIV integrase enzyme and inhibit it from inserting viral DNA into the human genome. Currently, there are five integrase inhibitors approved by the FDA.^2^ Raltegravir and elvitegravir, first-generation integrase inhibitors, which were approved in 2007 and 2012, respectively, followed by newly approved on-demand second-generation integrase inhibitor dolutegravir (2013) and its analogues, bictegravir, approved in 2018, and cabotegravir, approved in 2021.^3,4^ Considering the high demand of INI drugs, particularly dolutegravir, which is currently recommended by the World Health Organization (WHO) for the first-line treatment of HIV initiating antiretroviral therapy and their forthcoming patent expiration, their process research has drawn a lot of attention.

**Figure 1:**
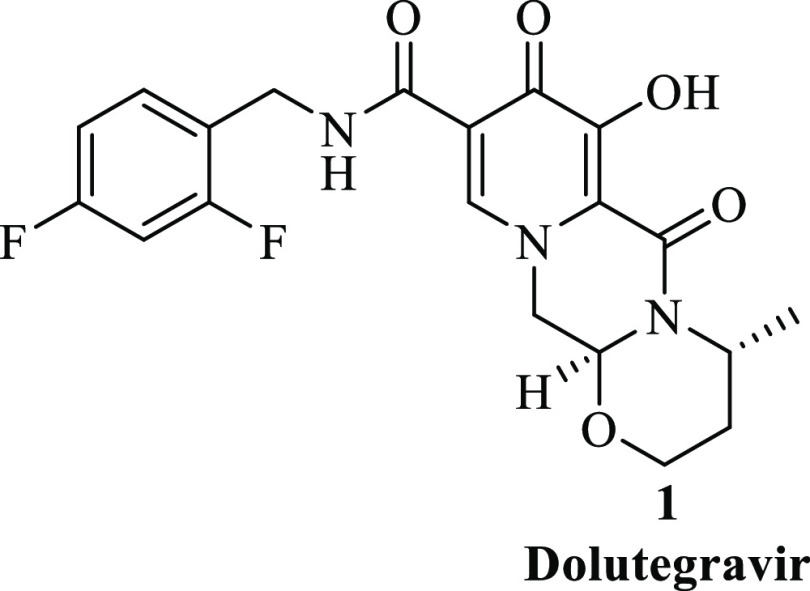
Dolutegravir **1** structure.

Numerous batch synthetic approaches to dolutegravir **1** have been documented and reviewed in the literature (Figure 1).^5−9^ Recently, applications of continuous flow processing toward the formation of dolutegravir have been reported.^3,10^ The reported continuous flow methods of dolutegravir are synthesized following a similar strategy:^3,10^ first, constructing a pyridone ring over a three-step procedure, which is then cyclized using 3-(*R*)-amino-1-butanol, followed by four sequential chemical reaction transformations, to the formation of the desired dolutegravir.

In 2018, a seven-step continuous flow synthesis toward dolutegravir was published by Ziegler et al.^3^ This was developed by adapting the research published by Wang and co-workers from GSK.^11^ The process constituted three separate flow operations in 24% overall yield, wherein the first flow operation included the first three steps, a continuous flow-through process for the production of pyridinone intermediate in 57% yield overall, which was isolated by crystallization in a total residence time of 74 min. The key features of this flow process are direct amidation of the ester to reduce the step count, rapid manufacturing time, and separation of the acetal deprotection formation flow reactors to attain high reactivity and selectivity for the tricyclic product.

While the synthesis of dolutegravir when performed under continuous flow conditions was successfully accomplished with great success, this developed process has several limitations. These limitations are the introduction of expensive fluorinated benzylamine during the early stages of the reaction. (*R*)-3-aminobutan-1-ol and 2,4-difluorobenzylamine are the major cost drivers during the production of dolutegravir, with (*R*)-3-aminobutan-1-ol contributing nearly 30% of the overall cost.^12^ An improved approach to circumvent this issue is therefore highly desirable, wherein the expensive fluorinated reagent is introduced at a later stage. A patent application by Cipla was published in 2019;^10^ similar to the flow procedure of Ziegler and coauthors, the process begins from the preparation of pyridone-acid intermediate, followed by another four-step route to dolutegravir. Their total semicontinuous process was successfully achieved in less than 50 min, making this approach a major player in the process developments toward dolutegravir.

As mentioned, the flow routes to dolutegravir in the work by Ziegler et al. and in a Cipla patent application both begin with the construction of pyridone **3,** followed by subsequent five steps toward the desired drug. Recently, Sankareswaran et al. published a five-step synthetic route based on a densely functionalized pyridinone as the starting material.^8^ To the best of our knowledge, no continuous flow method for constructing dolutegravir from this readily available benzyl-protected pyran **2** starting material has yet been reported. Production of dolutegravir from a different precursor, as opposed to the already reported flow routes, is worth attempting to expand the scope of the development of this API. We postulate that adopting the use of pyran **2** would result in an alternate route constituting a fewer steps and hence a shorter development cycle.

**Scheme 1:**
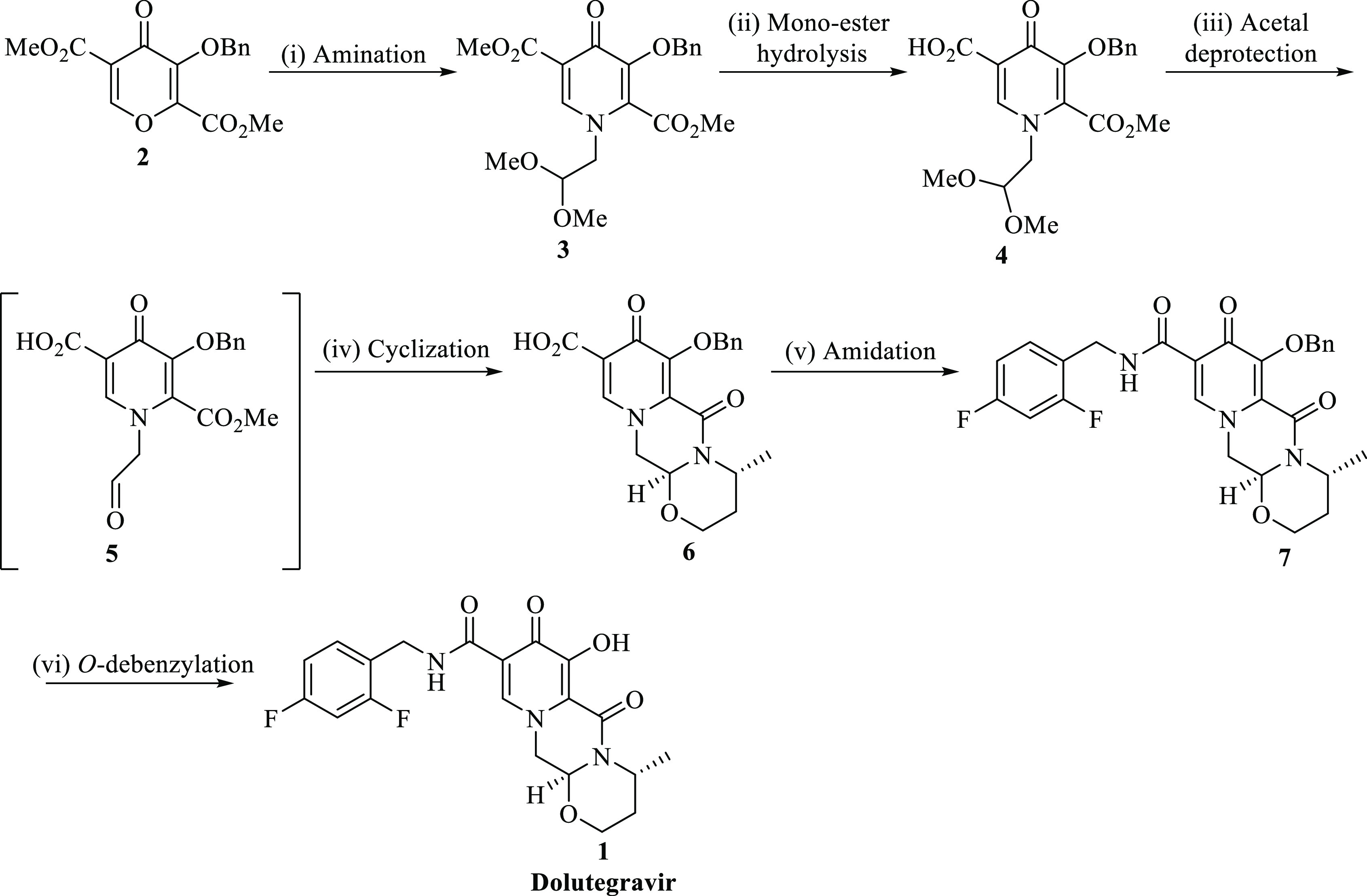
Synthetic Route of Dolutegravir **1**

Herein, we seek to establish a new efficient approach toward integrase inhibitors dolutegravir **1** from a readily available starting material, which when fully optimized will provide a step change in pharmaceutical manufacturing technology. It is expected that such continuous-flow synthetic routes toward dolutegravir will enable companies to provide greater access to dolutegravir-containing combination therapies in a shorter development cycle and higher yields. The route was derived from a batch research work by Sankareswaran et al.^8^ and Wang et al.^11^ and depicted in Scheme 1 with modifications.

## Results and Discussion

### Batch Synthesis of Dolutegravir

Our study began by developing and analyzing a batch process for dolutegravir, which was started from a commercially available benzyl-protected pyran **2**. After the perusal of the literature of different processes that have already been published, the modified batch process of our study was initiated by adopting the route reported by Sankareswaran et al.^8^ Considering the similar conditions for the preparation of the first step for the envisioned method to their process, we employed the same strategy, and the desired pyridinone **3** was afforded in 86% isolated yield by reacting **2** with aminoacetaldehyde dimethylamine **8** in the presence of a base at room temperature using methanol as a solvent. In the work of Sankareswaran et al., the resultant pyridinone **3** was then reacted with 2,4-difluorobenzylamine **10** in the presence of acetic acid, but in our method, compound **3** underwent selective monoester hydrolysis using a base; the expensive fluorinated amine **10** was only introduced at a later stage of the synthesis. In addition to that, our modified approach was also motivated by the literature findings that the direct amidation of unactivated esters proved to be challenging in the flow.^13−15^

Acid **4** was obtained in 64% isolated yield by reacting **3** with LiOH at 0 °C for 4.5 h in methanol. This was followed by acetal deprotection and cyclization, which were done in situ. To achieve this, intermediate **4** was reacted with 62% aqueous sulfuric acid and 98% formic acid, which was used as both the solvent and the reagent at 5 °C for 3 h. This afforded aldehyde **5** in 65% isolated yield, which was subsequently cyclized after isolation by reacting with 3-(*R*)-aminobutanol **9** in the presence of acetic acid for 2.5 h at 100 °C to form 66% isolated yield of tricyclic acid **6**. The subsequent stage, namely, selective amidation of **6** with 2,4-difluorobenzylamine **10** to form amidoester **7** (33% isolated yield), proceeded in the presence of a coupling reagent (CDI) for 4 h. Lastly, the *O*-debenzylation reaction of benzyl dolutegravir intermediate **7** under acid conditions to form the free acid of dolutegravir **1** was conducted. This reaction was performed using trifluoroacetic acid at 39 °C over 2 h, affording dolutegravir **1** in 90% isolated yield.

Having successfully achieved the modified batch procedure to prepare standards toward dolutegravir **1**, with an overall reaction time of 34.5 h from step 1 to step 6, it was realized that long reaction times were required for the reaction to reach completion. Moreover, some intermediates required a large excess of reagents, and the products were attained in lower yields. For example, the first reaction step, the amination reaction of pyran **2,** gave pyridinone intermediate **3** in 86% isolated yield after 18.5 h at room temperature using methanol as the solvent. A long reaction time was required for this reaction step to reach completion. This was worrisome, as this is the first step of the synthetic route, which will affect the subsequent steps and therefore the overall reaction cycle or process time toward the final drug. With the understanding of the positive effect of using microreactors on shortening the residence times through the process intensification,^16−18,19^ it made sense to begin the investigation on the possible reduction of the residence time, while maintaining a high yield when employing continuous flow systems. Taking advantage of the already discussed benefits of flow chemistry over the traditional batch,^16,20^ the successful batch synthesis of the individual steps toward the desired drug **1** helped as a guide for the investigation and optimization when transferring the batch reactions into the flow.

### Continuous Flow Synthesis of Pyridinone **3**

**Scheme 2:**
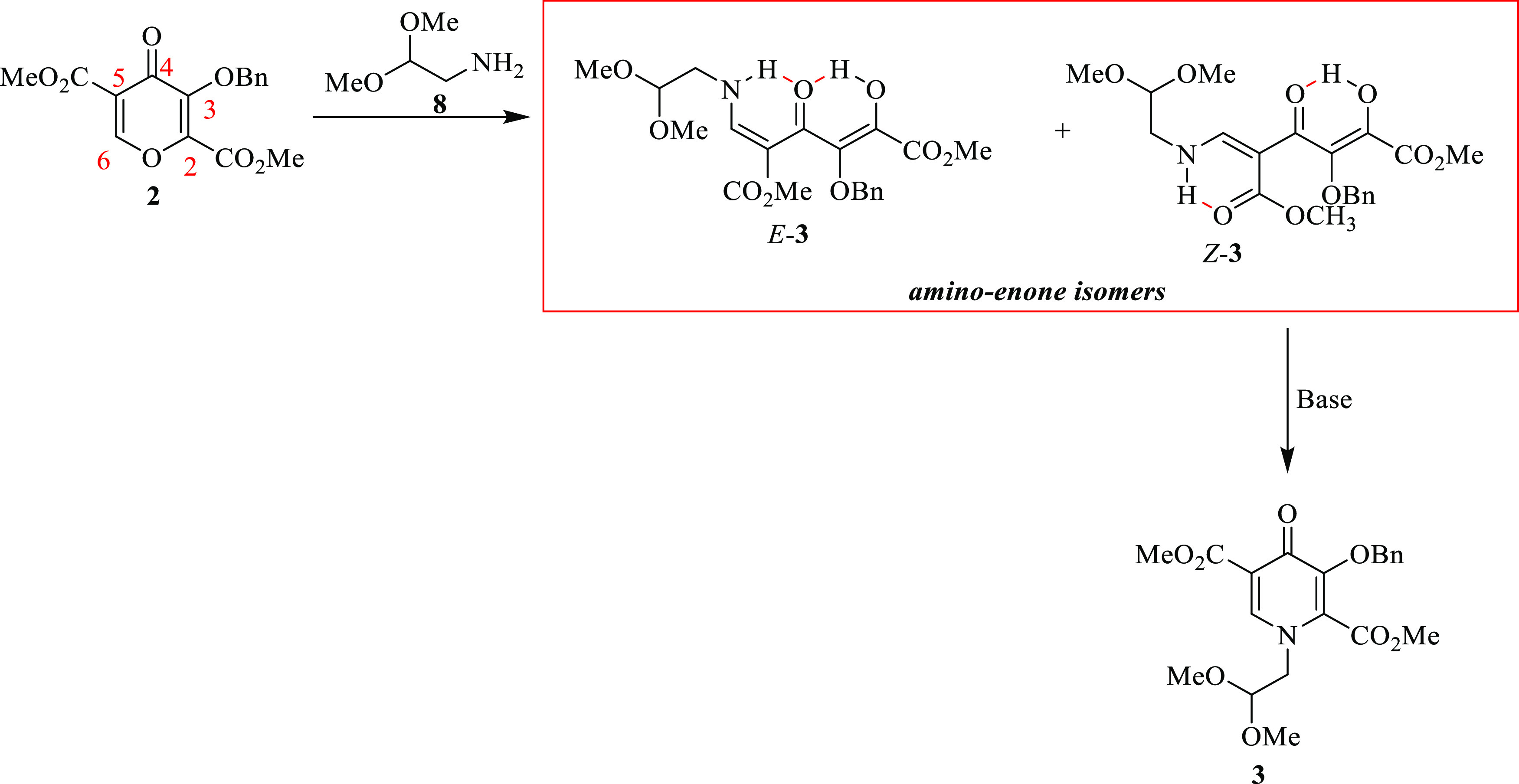
Schematic Diagram of the Proposed Process Isomers Formed in the Amination Stage 1 of the Process

This reaction step involves the formation of a mixture of in situ acyclic intermediates (*E*-**3** and *Z*-**3** isomers) formed upon the synthesis of pyridinone intermediate **3,** with a proposed reaction mechanism illustrated in Scheme 2. Pyran **2** consists of two electron-withdrawing carbonyls at positions 2 and 5, respectively, and some electrophilic centers, which have the ability to undergo 4-pyrone ring-opening transformation and cyclization. Mechanistically, treatment of **2** with amine **8** resulted in a ring-opening transformation; this is due to the pyrone ring that is highly electrophilic and thus readily reacts with *N*-nucleophiles to give a mixture of isomers *E*-**3** and *Z*-**3**. Strong intramolecular hydrogen bonds are formed from the labile protons involved during this transformation. The addition of *N′,N’*-diisopropylethylamine transformed amino-enone into the desired product dimethyl 3-(benzyloxy)-1-(2,2-dimethoxyethyl)-4-oxo-1,4-dihydropyridine-2,5-dicarboxylate **3** (Scheme 2).

**Figure 2:**
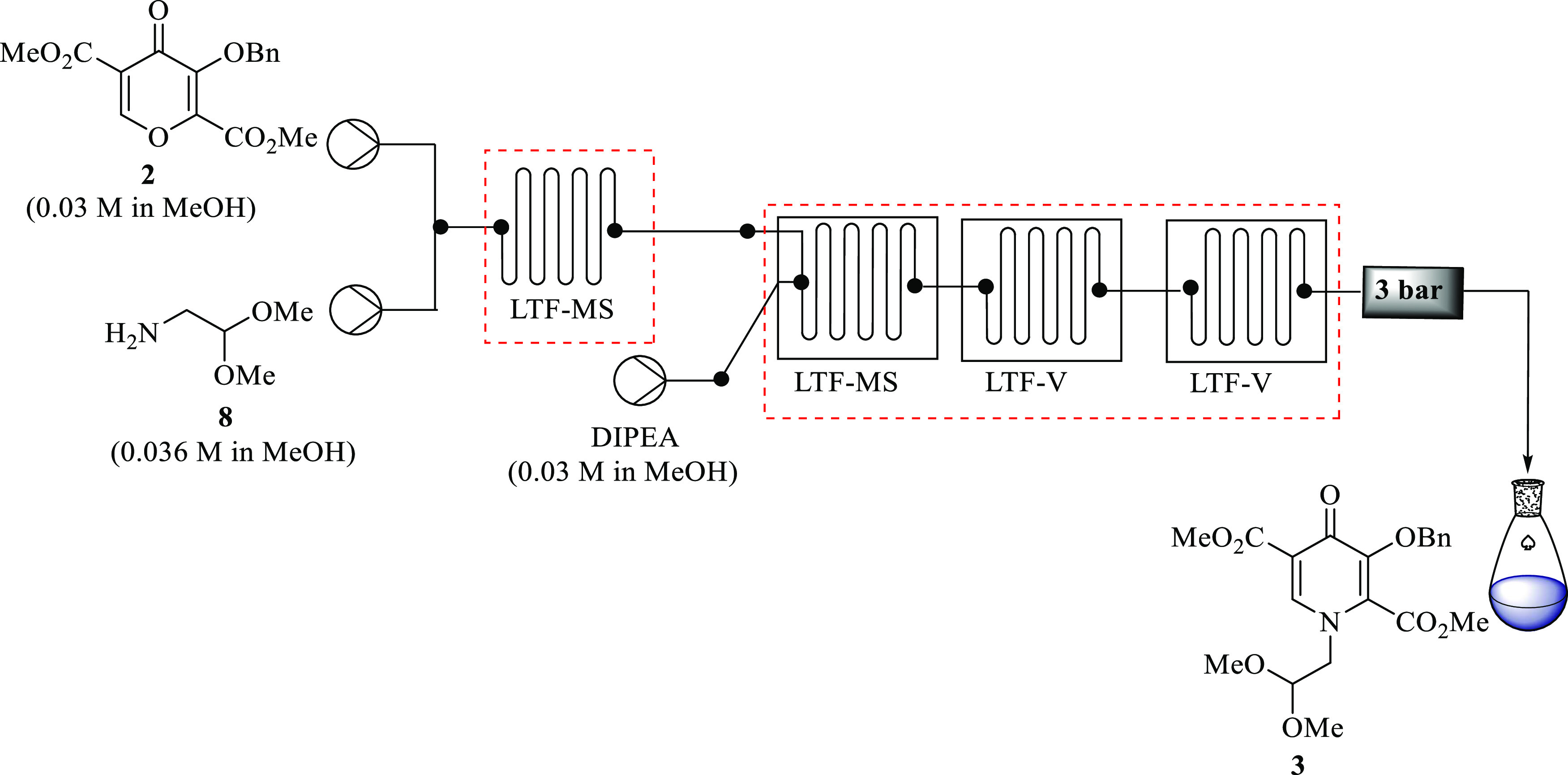
Continuous flow synthesis of pyridinone **3**.

Pyridinone **3** was prepared in continuous flow using the setup assembled as depicted in Figure 2 to conduct the preliminary optimization studies. In the preliminary experiment, pyran **2** (0.03 M, 1 equiv) was treated with aminoacetaldehyde dimethyl ether **8** (0.036 M, 1.2 equiv) to afford the in situ acyclic intermediates before the addition of a base. Both syringes were pumped at the same flow rate, and DIPEA (0.03 M, 1 equiv) was pumped at half the total flow rate of the first microreactor. These equivalent amount ratios were directly translated from the batch. A total residence time of 9 min was arbitrarily selected in the first instance, and the temperature was kept at 25 °C, similar to the batch temperature. Interestingly, 100% conversion of **2** was attained; however, selectivity toward the desired pyridinone intermediate **3** was less than 15%. This is due to the formation of the in situ acyclic *E-***3** and *Z-***3** intermediates, observed during the analysis, which required a longer reaction time to be cyclized to the desired product **3**.

**Figure 3:**
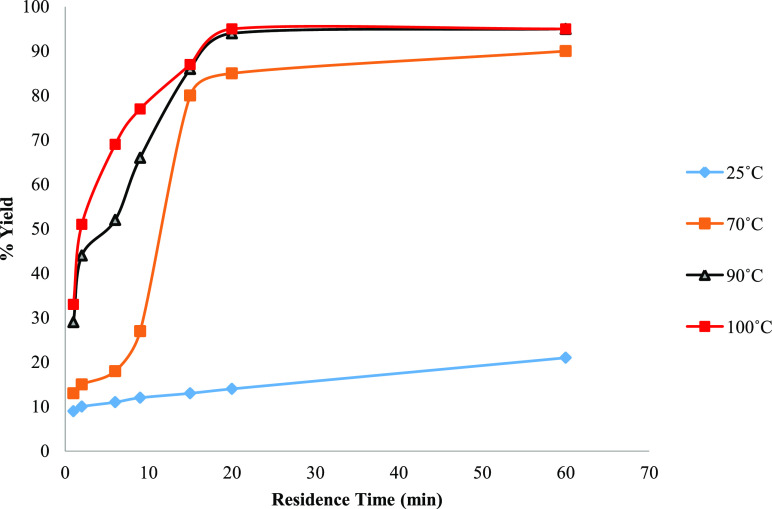
Investigation of the effect of increased temperature and total residence time on the amination reaction of pyran **2** in methanol.

With the results at hand, an intensive optimization study on the effect of residence time, temperature, and concentration was investigated. On the initial attempt, preliminary reaction conditions were kept constant (pyran **2**, amine **8,** and DIPEA at 0.03 M, 0.036 M, and 0.03 M molar concentrations, respectively, 25 °C), and the effect of increasing the residence time was examined. The conversion was 100% with a lower yield of 41% by HPLC toward the desired product **3** over a total residence time of 3 h at room temperature. The two acyclic intermediates were still observed. The results show that an increase in the residence time did not have a drastic effect on the yield of the desired product **3**; in addition, such long residence times are not practically feasible. Next, the effect of increasing the reaction temperature to 70, 90, and 100 °C was examined, with a backpressure regulator (3 bar) fitted to pressurize the system. All other parameters were maintained constant. It was realized that temperature is a crucial parameter in this reaction; thus, the observation showed that the formation of pyridinone **3** is thermodynamically favored. The best results were obtained at 100 °C in 20 min, with 95% yield (by HPLC) of pyridinone **3**. At residence times longer than 20 min, the reaction reached its plateau (Figure 3).

**Figure 4:**
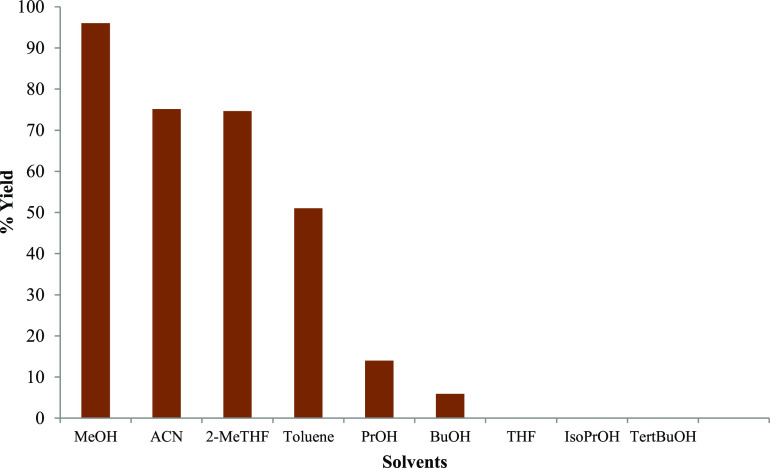
Solvent screening for the formation of pyridinone intermediate **3** from pyran **2** (0.03M) at 100 °C in 20 min.

Inspired by the results at hand, we also conducted an examination of the effect of performing this amination reaction using different solvents for the acceleration of the formation of pyridinone intermediate **3**. A series of different solvents, protic hydrocarbon, ester, and chlorinated solvents were examined. The results are summarized in Figure 4. The highest yield of 96% by HPLC was obtained, with methanol as the solvent, followed by acetonitrile and other alcohols. The trend pointed out that polar protic solvents were efficient for the reaction, especially alcoholic solvents. Methanol was considered the best solvent with its high dissolving ability, which was effective to also prevent the formation of any side products. This is consistent to the literature batch data of this reaction, wherein alcoholic solvent pointed out to be the most effective, particularly methanol.^8^ Interestingly, a trend was observed that as the polarity of alcoholic solvent decreased, i.e., with the increase of chain length (MeOH < propanol < butanol), the yield toward the desired product lowered. The reaction did not occur in all branched hydrocarbons examined.

When the reaction was carried out using toluene as the solvent, an overall yield of 51% by HPLC was obtained at 20 min residence time. The lower yield can be attributed to the nonpolar nature of this solvent. Other solvents were screened, and it was found that acetonitrile and 2-methyltetrahydrofuran were also viable. Acetonitrile and 2-methyl THF gave better yields than toluene, affording 75% (by HPLC) of **3,** respectively, but lower compared to methanol. However, 2-methyltetrahydrofuran is more expensive; using it as a solvent of choice would be of greater advantage, not only because of its high boiling point, high polarity, and Lewis base strength but also because of its ecofriendly nature. It has been well documented in the literature that this solvent is manufactured from renewable resources, and using it in an industrial scenario results in more than 97% reduction emission compared to typical tetrahydrofuran.^21^ The reaction did not occur when using the THF solvent itself.

Next, a study of alternative bases that can be used for the cyclization of acyclic intermediates was investigated. Using MeOH as a solvent, different bases, organic and inorganic, were explored at 100 °C. A series of tertiary alkylamines, other aromatic heterocyclic amines, and different inorganic bases was investigated and optimized (Table 1). Optimum conditions were found at 100 °C, and 2 min residence time afforded **3** in excellent percentage yield (HPLC) and 97% selectivity, using 1:1.2 equiv ratio of pyran **2** and amine **8** in the presence of KOH (1 equiv) as the optimum base (Table 1, entry 2). This reaction, when conducted in a traditional batch method, is done in 18 h using 1:1.2 equiv ratio of pyran **2** and amine **8** in the presence of DIPEA (1 equiv), affording 86% isolated yield.

**Table 1:** Effect of Different Bases on the Amination of 2 (0.03 M) with Amine 8 (0.036 M) at 100 °C Using Methanol

experiment name	time (min)	base (0.03 M)	% yield^a^
1	9	DIPEA	77
**2**	**2**	**KOH**	**97**
3	9	TEA	73
4	9	imidazole	58
5	2	NaOH	94
6	9	TBA	63
7	2	DBU	95
8	9	DBACO	62
9	9	THA	73

a Yield by HPLC.

### Selective Monohydrolysis Reaction of Pyridinone Diester 3 in Continuous Systems

**Figure 5:**
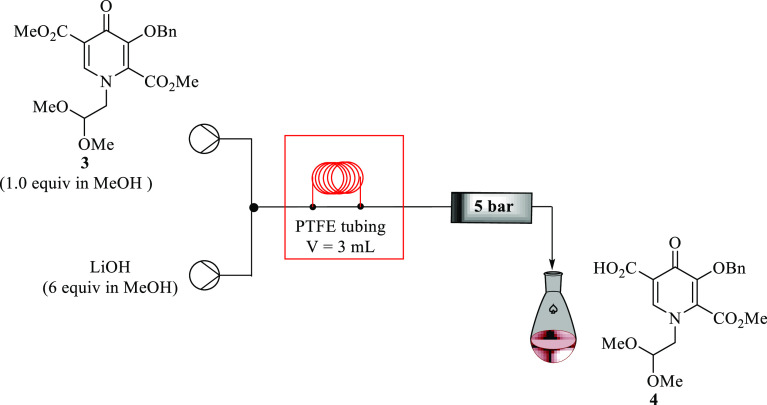
Continuous flow systems of selective monohydrolysis reaction of **3** in a PTFE tubing reactor.

After having fully optimized the first intermediate **3**, the next stage involved the development of a continuous flow synthesis of the second step of the synthetic route toward the formation of pyridinone acid **4**. Pyridinone acid **4** was prepared by treating **3** with a suitable base in a PTFE tubing reactor system (Figure 5, Table 2).

**Table 2:** Condition Screening and Optimization of Selective Mono-ester Hydrolysis Reaction of Pyridinone **3** in Continuous Flow^a^

experiment name	**3**/base^b^	base	temperature (°C)	time (min)	% yield^c^
1	1/6	LiOH	50	5	5
2	1/6	LiOH	75	5	14
**3**	**1/6**	**LiOH**	**100**	**5**	**100**
4	1/4	LiOH	100	15	100
5	1/3	LiOH	100	20	100
6	1/3	KOH	100	20	100
7	1/3	NaOH	100	20	84
8	1/3	TBAOH	100	20	41
**9**^d^	**1/3**	**KOH**	**100**	**1**	**98**–**100**
10^e^	1/3	KOH	100	1	98–100

a Standard conditions: feed one—pyridinone (0.1 M, 1 equiv), feed two—base, and using methanol as a solvent in both feeds.

b Molar equivalence.

c Carboxylic acid **4** percentage yield determined by HPLC using a synthetic standard.

d Cosolvent mixture (2:9 ratio; MeOH: H_2_O (V/V)).

e Cosolvent mixture (10:1 ratio; MeOH: H_2_O (V/V)).

Initially, guided by the batch conditions, a solution of **3** in methanol (0.1 M, 1 equiv) and a methanol solution of LiOH (0.6 M), each delivered at varying flow rates, were combined in a T-piece and passed through the tubing reactors one and two wherein the temperature was maintained at 0 °C to allow mixing. Under these conditions, the reaction progression was unsuccessful after 25 min residence time. An increase in temperature from 0 to 100 °C afforded pyridinone acid **4** in full conversion and 100% yield by HPLC in only 5 min residence time (Table 2, Entry 4). Increasing the residence time beyond did not affect the reaction. Noteworthily, a ratio of 1:6 equiv of the starting diester **3** to lithium hydroxide was used for the reaction to go to completion. The overuse of the base despite the transfer from batch to flow inspired us to further conduct a study on the reduction of the amount of a base while maximizing the reaction conditions with the aim to still attain **4** at high yields. The desired acid **4** was obtained again in 100% yield by HPLC in 20 min when 3 equiv of LiOH was employed (Table 2, Entry 5); however, a further decrease in the equivalent was accompanied by a decrease in the conversion of **3**. While the residence time may seem unimpressive, it is worth noting that **4** still attained 100% yield by HPLC while using 3 equiv of LiOH, which is half of the amount used in batch for 4.5 h. Three equiv amount of LiOH was chosen as an optimum to further the investigation.

A series of different bases were introduced to investigate the effect on the hydrolysis of **3**: potassium hydroxide, sodium hydroxide, and TBAOH at the same conditions. The use of KOH afforded the best results, and the use of TBAOH gave very low yields (41% by HPLC). LiOH is a common base that has been used in the literature to effect the selective hydrolysis of a similar compound because it is stated to improve the chemoselectivity. For instance, Wang et al.^11^ demonstrated the use of LiOH for this similar reaction in a one-pot batch operation over four steps to afford the desired product in 61%. Recently, Kong and co-workers stated in their batch study toward a dolutegravir intermediate that LiOH presented satisfactory results; however, the reaction time was very long (24 h). They attempted the use of KOH; however, their results revealed a very low selectivity with a high percentage of unwanted diacid byproducts.^22^

In the case of this flow study, it was therefore concluded that KOH was the best base for the hydrolysis study of **3**. The advantages of using potassium hydroxide is that it is somewhat environmentally friendly and allows the straightforward formation of **4**.

The use of cosolvent (MeOH/H_2_O) was investigated. There have been very few studies reported about the water-mediated desymmetrization reactions, wherein a quantity of water is added as a cosolvent to accelerate the reaction.^14,23,24^ The increase in the reaction rate is attributed to the hydrophobicity of diesters. With the results obtained and the knowledge at hand, this encouraged us to investigate the effect of the water-mediated hydrolysis reaction to form **4**. The reaction was carried out at 100 °C in methanol at 1:3 equiv of pyridinone **3** to KOH. A cosolvent ratio of 2:9 (methanol: water) was arbitrarily selected in 3 min to begin the study. The synthesis afforded the highest yield by HPLC and selectivity of **4** (98%) compared to 29% obtained in the absence of water at the same reaction conditions. It was evident that indeed the presence of water as a cosolvent had an effect in enhancing the formation of the desired **4**. Next, the proportion of methanol as a cosolvent was changed by increasing the amount of water while keeping the total volume of the reaction mixture constant. It was apparent from the results that increasing the content of water did not have a significant effect on the yield, as it was only slightly greater by small increments. The results showed that the addition of water as a cosolvent to methanol afforded **4** in higher yields and selectivity (98–100%) in at most 1 min residence time at 100 °C (Table 2, Entry 9).

The optimum conditions established are 1:3 molar equiv of pyridinone diester **3** to potassium hydroxide at 100 °C to afford 100% carboxylic acid **4** by HPLC in 20 min when using methanol or alternatively 98–99% by HPLC in 1 min when using the methanol–water cosolvent (2:9 ratio, methanol: water). This is very advantageous as the successful ability of conducting this reaction in a water cosolvent makes the approach more environmentally friendly and represent green chemistry. The same reaction when conducted in batch afforded **4** in 64% isolated yield at 4.5 h using 6 equiv of LiOH. As aforementioned, in one recent batch study, when KOH was used, it gave low selectivity; however, in this study, KOH also proved to be better. This can be attributed to one of the advantages of using flow, where reactant ion conditions (temperature, residence time, and concentration) were accurately controlled and subsequently resulted in accelerated rates and improved selectivity and yield. Selective monohydrolysis of **3** in continuous flow systems was therefore successfully achieved at reduced reagent volume (molar equivalence), higher yields, and selectivity in a reduced reaction time.

### Two-Step Synthesis of Pyridinone Acid **4** from Pyran **2** via Pyridinone **3**

Encouraged by the excellent yield attained at shorter residence times during the single-step continuous flow synthesis of amination and selective ester hydrolysis, respectively, the next stage was to investigate the possibility of synthesizing pyridinone acid **4** directly from pyran **2** via pyridinone **3** in a single step. This was achieved after obtaining the optimized continuous flow process for each step. As briefly discussed in the previous section, pyridinone **3** was formed in 97% yield by HPLC under the following flow conditions: pyran **2** (0.03 M), aminoacetaldehyde dimethyl acetal **8** (0.03 M, 1 equiv), and KOH (0.03 M, 1 equiv) at 100 °C in 2 min in methanol using the LTF reactor system. Under the optimized conditions of 100 °C in 1 min residence time with pyridinone **3** (1 equiv) and KOH (3 equiv), pyridinone acid **4** was obtained in 100% yield by HPLC using the LTF reactor system.

**Figure 6:**
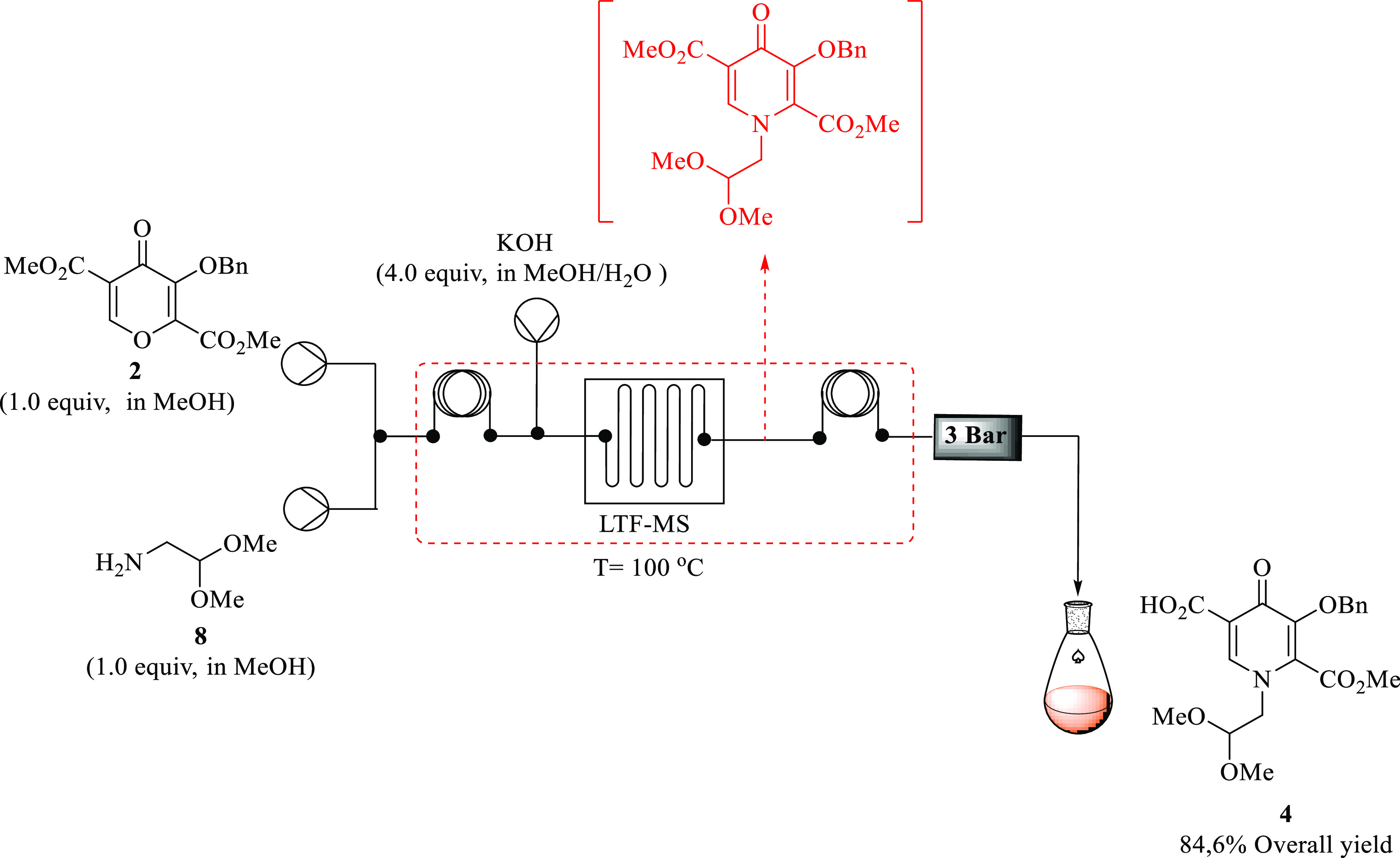
Two-step synthesis of pyridinone acid **4** from pyran **2**.

Given the complexity of the individual reaction setup of these two steps at optimum conditions, some challenges that would experience when telescoping these systems were foreseen, including the pressure challenges due to the many reactors used in these systems at high temperatures. To circumvent the issue beforehand, the first thing was to develop a simpler setup that would allow the reaction to be conducted under the same conditions. The setup was assembled as illustrated in the Figure 6, by combining the PTFE tubing reactor and LTF-MS reactor, giving a total volume of 3 mL of the system.

The abovementioned optimum conditions were used, and this allowed the initial amination to afford **3**, which was hydrolyzed to afford the desired **4**. However, when using these conditions, the system was stalling due to high pressure, which resulted from flowing reactions at a fast flow rate to maintain a shorter total residence time of 2 min for amination (mixing **3** and **8** in the presence of excess KOH over 1 min) and hydrolysis (reaction of the eluent with excess KOH over 1 min) attained during the optimization of individual steps.

The reactants were then pumped at slower flow rates and therefore longer residence times. The results attained are summarized and illustrated in Table 3. Formation of **4** was dependent on the increase of residence time obtained in an approximate yield of 30, 51, and 85% by HPLC at 6, 15, and 20 min total residence time, respectively, at 100 °C. Full conversion was attained; however, the selectivity dropped to at most 85% in 20 min compared to the expected higher selectivity in a shorter time, as formed when optimizing the reaction steps individually. Increasing the equivalence of KOH was investigated;, however, this did not improve the yield, but a trend of the decrease in selectivity was noticed. An impurity was observed, however, with no evidence of pyran **3** or **4** in the sample product.

**Table 3:** Multistep Synthesis of Pyridinone Acid **4** from Pyran **2** via Pyridinone **3** in Methanol/Water Cosolvent

experiment name	total residence time (min)	temp (°C)	KOH equiv	selectivity (%)
1	2	100	4	- (stalling)
2	6	100	4	30
3	15	100	4	80
**4**	**20**	**100**	**4**	**85**
5	6	100	6	52
6	15	100	6	49
7	20	100	6	44

The feasibility of the direct formation of pyridinone carboxylic acid **4** from the starting pyran **2** with pyridinone **3** formed in situ through a multistep synthesis in continuous flow systems was demonstrated. At most, **4** was achieved in 85% yield by HPLC in 20 min at 100 °C using a methanol–water solvent mixture (Table 3, Entry 4). The optimum ratio between methanol and water was 2:9. When these two steps were synthesized in batch, they were attained in a total 22.4 h reaction time, affording **3** in 86% isolated yield and **4** in 64% isolated yield, respectively. While there was a decrease of the optimum selectivity of **4** during the multistep synthesis, it is worth noting that these results highlight the advantage of using continuous flow in reducing the overall reaction time compared to their overall batch synthesis.

### Acetal Deprotection Pyridinone Acid **4** in Continuous Flow Using Formic Acid

**Figure 7:**
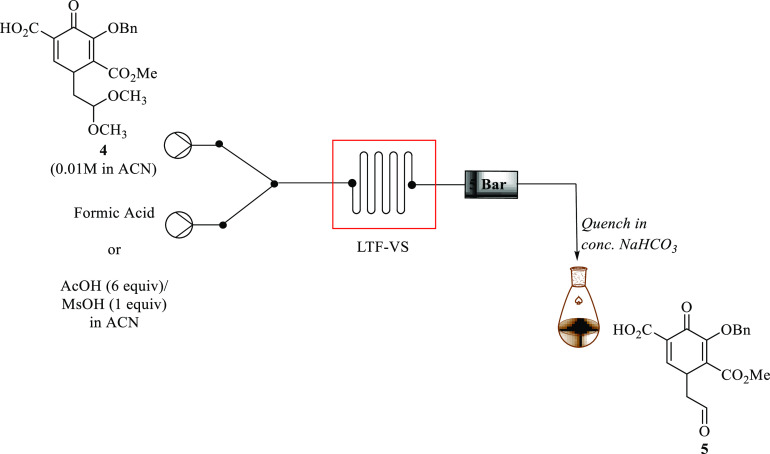
Continuous flow acid-mediated acetal deprotection in a LTF reactor.

The acetal deprotection of **4** using either formic acid, methanesulfonic acid/acetic acid (Figure 7), or acidic resin catalysts (Amberlyst-15, A-36, or A IR-120, respectively) was investigated in continuous flow to afford compound **5**. In the preliminary experiments, a solution of acid **4** (0.01 M) in acetonitrile was reacted with neat formic acid using an LTF microreactor to facilitate the reaction at 25 °C for 1 min. This led to the formation of aldehyde **5** in 81% yield by HPLC.

For reaction optimization, first, an effect of increasing the residence time at 25 °C was investigated. It was observed that increasing the residence time up to 30 min gave the same results. Raising the temperature to 50 and 75 °C, respectively, resulted in full conversion and afforded the desired aldehyde **5** in 100% yield (by HPLC) in 1 min, and the reaction reached its plateau. Next, this reaction can be conducted at an even shorter residence time using a temperature of 50 °C while keeping all other parameters the same. At best, the reaction could be done in 30 s which led to **5** in 100% yield by HPLC. When the reaction was done at residence times shorter than 30 s, there was a pressure buildup due to very high flow rates, and the system was stalling.

**Figure 8:**
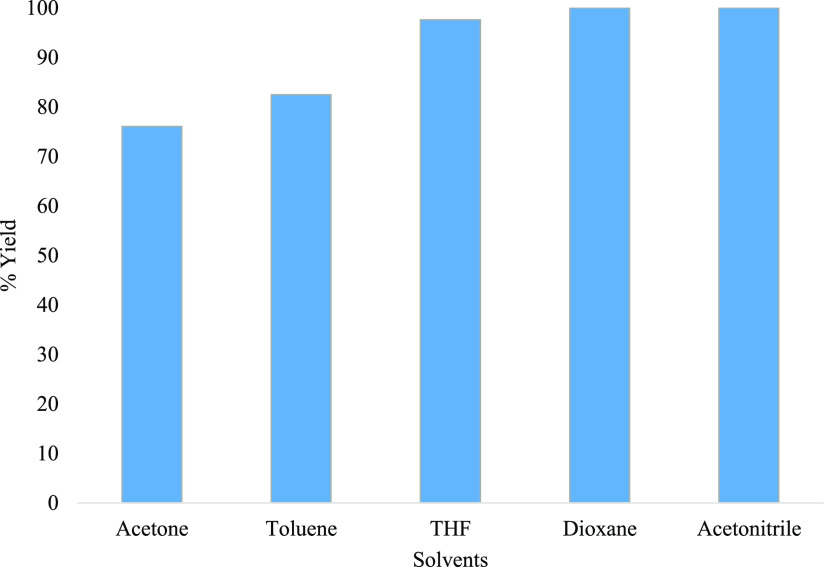
Solvent screening for the formation of aldehyde **5** at 50 °C in 30 s.

To expand the scope of the investigation, a study of the effect using different solvents to carry out this reaction was examined. A series of solvents were examined while keeping the concentrations constant, and the reaction was carried out at 50 °C in 30 s. The results showed that the highest yield was obtained when using acetonitrile or dioxane, which afforded **5** in 100% yield by HPLC, respectively (Figure 8). Using toluene and acetone gave slightly lower yields of 82 and 76%, respectively. Since the grand goal was to telescope this step with the following cyclization step of **5** which was achieved using acetonitrile, this common solvent was chosen as the optimum instead of choosing dioxane or THF. After ascertaining that acetonitrile is still the best solvent, the effect of decreasing the concentration of formic acid was then investigated. The molar concentration of neat 98% formic acid used was effectively 23.6 M, and the goal was to synthesize **5** using formic acid in low concentration while maintaining high yields by HPLC and shorter residence times. The results clearly show that the reaction can be done using a lower formic acid concentration and full conversion, and 100% yield by HPLC of the desired **5** was observed when using 15 M. In contrast, when using a 10 M concentration and below, a yield of 60% by HPLC and less was attained. Therefore, at best, 15 M formic acid was required, and this is a huge improvement as opposed to that when using it neat. Optimum conditions established for the acetal hydrolysis reaction step were pyridinone acid (0.01 M) and formic acid (15 M) at 50 °C at full conversion to afford 100% yield of **5** (by HPLC) in 30 s using acetonitrile as the best solvent. Noteworthily, when this same reaction was conducted in batch, **5** was obtained in 65% isolated yield using 98% neat formic acid as a reagent and solvent in the presence of 62% H_2_SO_4_ at 0 °C in 3 h.

### Acetal Deprotection of Pyridinone Acid **4** in Continuous Flow Using Acid Resin Catalysts

**Figure 9:**
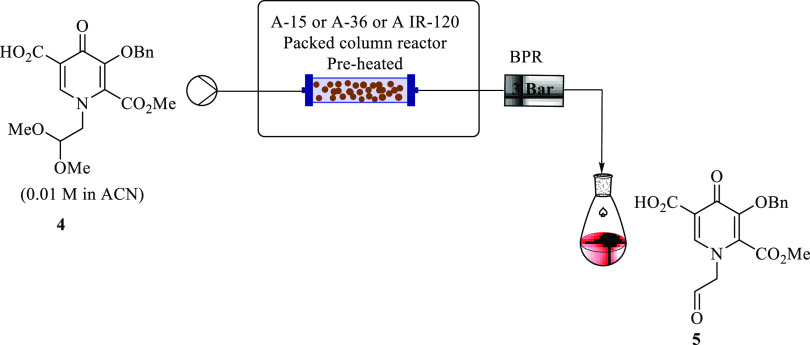
Continuous flow synthesis of aldehyde **5** using acid resin catalytic column systems in acetonitrile.

Guided by the optimum conditions obtained for acetal deprotection using Little Things Factory (LTF) microreactor continuous flow systems, aldehyde **5** was synthesized in a Uniqsis packed bed continuous flow system packed using acidic resin catalysts (Amberlyst-15, A-36, or A IR-120 respectively). A flow system was assembled, as depicted in Figure 9.

As the first tentative investigation, the following flow conditions were used: 50 °C, packed with Amberlyst-15 (2.6 g), pyridinone acid **4** (0.01 M), and acetonitrile as a solvent. Under these conditions, the reaction did not work. The effect of performing the reaction at higher temperatures while keeping the other parameters constant was then examined. The reaction was done at 80, 100, and 110 °C, respectively. Fresh Amberlyst-15 was used for each run, and used columns were regenerated for reuse. The study was not extended beyond this to specifically determine the life span of each Amberlyst-15 packed column reactor with usage. During the analysis, pyridinone acid **4** conversion was 100% at 100 and 110 °C; however, low selectivity of at most 33 and 48% was attained at 5 min residence time, respectively. A complex mixture of unwanted unidentified byproducts was observed. At 80 °C, the reaction did not occur. When the reaction was done at different residence times ranging from 1 to 20 min at respective temperatures (80, 100, and 110 °C), while keeping the concentrations, solvents, and catalyst resin the same, it was observed that selectivity dropped with an increase in residence time. Many unidentified byproduct peaks were observed, with no evidence of the starting material.

To circumvent the issue, alternative resin catalysts were explored, thus with packed A-36 and A IR-120, respectively, instead. Again a similar trend to Amberlyst-15 results was observed during the analysis for both catalysts: full conversion, however with very low selectivity, with a lot of side products. Changing different parameters, such as temperature and residence times, disappointedly did not improve the desired **5** flow synthesis. The results pointed out that using acidic catalyst resins does effect acetal hydrolysis but with a high content of complex byproducts and low selectivity. Using formic acid in LTF flow systems still proved to be the best; hence, we opted to use this approach to further the subsequent reaction.

### Formation of Benzyl Dolutegravir **5** Using Formic Acid or MSA and AcOH

**Figure 10:**
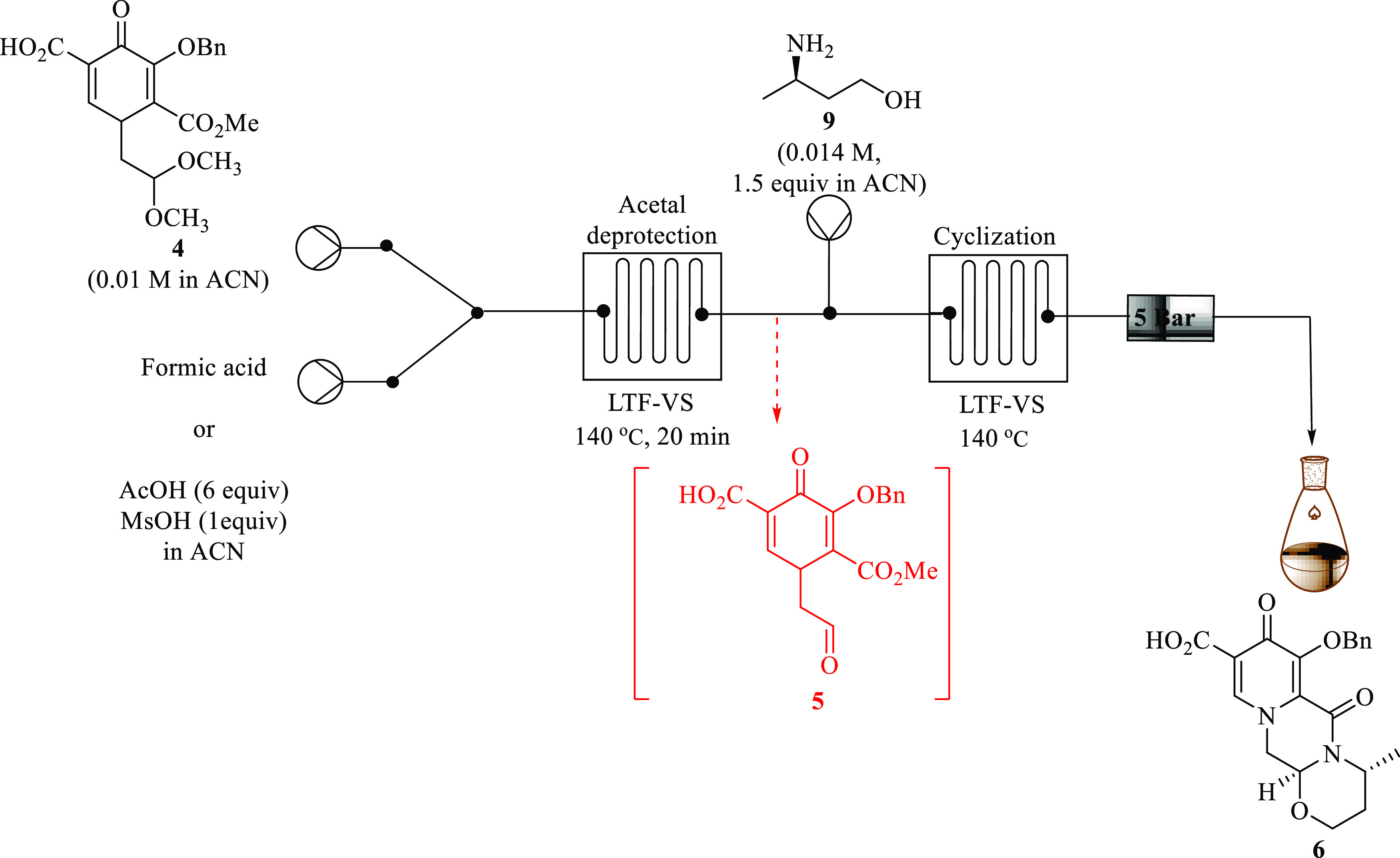
Continuous flow synthesis of intermediate **6** from acid **4** via aldehyde **5** formed in situ.

The next reaction is the diastereoselective cyclization of aldehyde **5** with 3-(*R*)-aminobutan-1-ol **9** to form benzyl dolutegravir **6** (Figure 10). Keeping the flow optimum conditions for the acetal deprotection reaction transformation in the first microreactor, a solution 3-(*R)*-aminobutanol **9** (0.014 M, 1.42 equiv) in acetonitrile was introduced in the stream and allowed to react with the resultant aldehyde in the second microreactor at half the total flow rate of the first reactor. During preliminary investigation, the reaction was conducted at 50 °C for both reactors in 3 min total residence time (reactor 1: 30 s, and reactor 2: 2.5 min). Disappointedly, there was no evidence of the desired product **6**. An effect of increasing the residence time of the second reactor while maintaining the first reactor and all other parameters the same was investigated. Again, the reaction did not occur, and the desired intermediate **6** was not attained. Unreacted aldehyde **5** was observed.

Next, keeping the residence time and temperature of the first step the same (30 s and 50 °C), the cyclization reaction with 3-(*R*)-aminobutanol **9** was conducted at increased temperatures and time. The temperatures were varied to 75, 100, 120, and 140 °C at varying residence times between 2.5 and 19.5 min. However, the best results afforded 92% conversion of aldehyde **5** and 34% yield by HPLC of intermediate **6** and at 3 min total residence time at 120 °C.

Conducting the reaction at longer residence times did not improve the selectivity. The major challenge was the formation of an unidentified complex mixture of byproducts produced during the transformation of acid **4** to desired **6** via aldehyde **5** as the temperature increased. It was reasoned that the acidic nature of the resultant mixture of the first step due to the use of formic acid in high concentrations affects the side reactions.

In the reported literature on batch routes toward dolutegravir, Sankareswaran and co-workers^8^ detailed that the temperature and the acidic nature of the reaction mass attribute to the formation of the side reaction. They reported that tuning parameters such as molar equivalence of the acid and temperature would minimize the formation of impurities; however, during this study, both investigations did not improve the yield. To eliminate this challenge, an alternative method toward the formation of intermediate **6** from **4** via aldehyde **5** was explored.

Pyridinone acid **4** (0.01 M) in acetonitrile was treated with a premixed solution of glacial acetic acid (0.06 M, 6 equiv) and methanesulfonic acid (0.01 M, 1 equiv) in a thermally controlled microreactor system (Figure 7). Initial studies using the batch temperature (65 °C) showed that the reaction was not successful; unreacted **4** was recovered. The conversion of **5** to **6** improved with an increase in temperature and the residence time, and the optimum conditions were found to be 140 °C and 30 min residence time. A 100% conversion afforded the desired aldehyde **5** in 67% yield (HPLC). Controlling the formation of the undesired side products to effect an increase in selectivity toward **5** proved to be challenging. Nevertheless, the study demonstrated that acetal deprotection using this method can be successfully done in continuous flow, affording the desired **5** in full conversion, and improved yield can be successfully obtained using a continuous flow system.

Having successfully optimized the acetal deprotection of **4** to aldehyde **5**, the next step was to form the corresponding tricyclic intermediate **6** by treating the optimized **4** with 3-*R*-aminobutanol **9**. An LTF continuous flow system was used to optimize the synthesis of tricycle **6** from the optimized aldehyde intermediate **15** (Figure 10).

Keeping the optimum conditions of the acetal deprotection step (pyridinone acid **4** (0.01 M), acetic acid (0.06 M, 6 equiv), and methanesulfonic acid (0.01 M, 1 equiv) over 20 min residence time at 140 °C), the resultant reaction mixture was further pumped into a second LTF-VS reactor and cyclized with an addition of a solution of 3-*R*-aminobutan-1-ol **9** (0.014 M, 1.42 equiv) at half the total flow rate of the first microreactor through a T-mixer. The temperature for both reactors was kept at 140 °C. Full conversion (100%) of **4** was observed within 3 min of residence time. The best results afforded 71% selectivity (HPLC) of the desired tricycle **6** at 5 min residence time (Table 4, Entry 5). At the residence time beyond 5 min, the selectivity did not improve but plateaued. The lower selectivity was due to the presence of an unidentified complex mixture of byproducts, which proved to be difficult to isolate. This continuous flow procedure proved to be superior to the long batch procedures which afforded **6** (66%) after 5.5 h when using formic acid and 40% after 36.5 h when using acetic/methanesulfonic acid.

**Table 4:** Continuous Flow Synthesis of Tricycle 6 From Pyridinone Acid **4** via Aldehyde **5** in Acetonitrile

reactor 2 residence time (min)	temp (°C)	selectivity (%)
1	140	- (stalling)
3	140	64
**5**	**140**	**71**
7	140	71
9	140	73

### Amidation Reaction of Pyridinone Tricycle Acid **6** in Continuous Flow Systems

**Figure 11:**
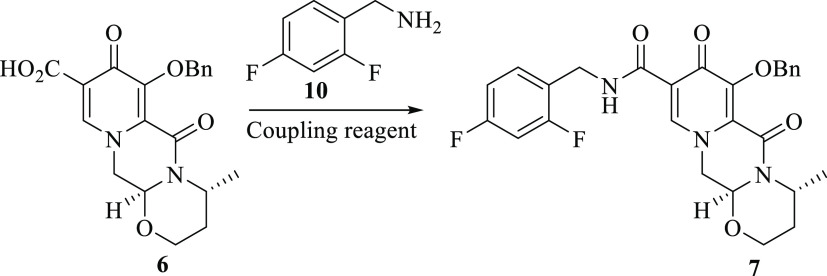
Amidation of tricycle **6** in the route to the synthesis of dolutegravir.

After having successfully synthesized tricyclic dolutegravir intermediate **6** using continuous flow, the next step of the synthesis was the formation of dolutegravir amide **7** (Figure 11). General amide bond formation typically occurs through the reaction of a carboxylic acid and an amine. However, as well documented in the literature, such reactions only take place at elevated temperatures (e.g., temperatures above 200 °C) due to the necessary removal of water involved in the process, hence the activation process of carboxylic acid came into play.^25^ The preactivation process is carried out by converting the hydroxyl group of the carboxylic acid into a good leaving group prior to reacting with an amine. Various methods for the carboxylic acid activation include coupling via acid chlorides, with thionyl chloride and oxalyl chloride being the most commonly preferred chlorinating reagents.^26−28^ Another popular method of activation is via acyl imidazole, wherein carbonyl diimidazole (CDI) is used.^25,26,29^ In addition, other activation methods via mixed anhydride, activated ester, carbodiimide, phosphonium salt, guanidinium, and uronium salt have been well documented in the literature.^25,26,30,31^

The continuous flow process involves a two-step system, whereby carboxylic acid is initially preactivated and then telescoped to amidation. Development of a flow process of this step was also inspired when Phull et al.^12^ reported amide synthesis from carboxylic acid via the continuous flow process toward dolutegravir formation in a patent application in the midst of this research.^10^ While they demonstrated a successful flow process, their method involved the formation of the amide via mixed carbonic acid anhydrides, wherein carboxylic acids were reacted with ethyl chloroformate in the presence of *N*-methylmorpholine as a base. Their approach was definitely a success in the goal to develop a continuous flow process toward dolutegravir formation, affording the desired amide in good yield (optimum 80% yield by HPLC) after 1.15 min by reacting the acid intermediate with ethyl chloroformate (1.31 equiv) and amine **10** (1.4 equiv) in the presence of *N*-methylmorpholine base (1.4 equiv) at 0 °C. However, as aforementioned, there are various methods that can be used for amide bond formation. It was therefore evident that further research was needed for the development of an alternative process to address this gap and therefore provide an alternative novel approach toward dolutegravir formation that would be convenient, simple, high-yielding, selective, and economically viable.

In this study, due to the lower yield and longer reaction time required in the batch process when using CDI, it made sense to begin the investigation by translating this into flow. There is no literature on the continuous flow synthesis of the dolutegravir amide via CDI. Moreover, this was a very advantageous process because CDI is cheaper compared to ethyl chloroformate. The adaptation of this novel coupling process toward dolutegravir amide **7** formation and therefore the exploration of other alternative methods was demonstrated. The use of CDI, COMU, PyOxim, and triphosgene toward acid activation was explored.

### Preactivation of Tricycle Intermediate **6** in Continuous Flow

**Figure 12:**
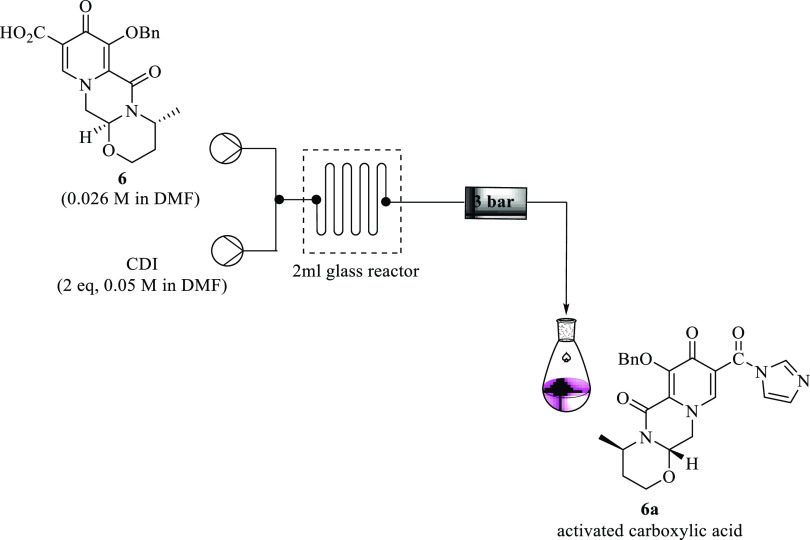
Activation of tricycle pyridinone acid **6** using CDI in a continuous flow.

Preactivation of acid **6** using CDI was investigated in a Uniqsis continuous flow system (Figure 12). In the preliminary studies, acid **6** (0.026 M, 1 equiv) was treated with CDI (0.05 M, 2 equiv) using anhydrous DMF as a solvent of choice at 80 °C. To start with, the effects of the residence time and temperature on the activation of **6** were examined.

An increase in the residence time and temperature did not have an effect on the reaction. Disappointingly, despite the use of the Uniqsis flow reactor, which allows intensive mixing, the reaction did not occur. Unreacted starting acid **6** was recovered. A study on the effect of increasing and decreasing the molar equivalence of the coupling reagent CDI on the reaction was conducted. For unknown reasons, the reaction was still unsuccessful, and unreacted **6** was observed. An introduction of a base in the reaction while employing CDI as an activating agent was also investigated. It was postulated that the base would allow the deprotection of the acid to an intermediate ion which would instantaneously react with CDI to afford the desired activated stable imidazole intermediate **6a**. DIPEA was arbitrarily selected as a strong inorganic base. After a series of reactions at varying residence times and temperatures, the reaction was unsuccessful.

It was successfully demonstrated that using coupling reagents to allow carboxylic acid preactivation prior to amide coupling in continuous flow systems and COMU worked remarkably well. Despite the relatively higher cost of COMU compared to CDI and other coupling reagents, this novel continuous flow procedure with COMU proved to be very advantageous with regard to the yields and the residence times. The reaction was straightforward as it reached completion in less than 30 s at room temperature in the presence of DIPEA. The detailed results of the optimum conditions obtained during the investigation are summarized in Table 5. To the best of our knowledge, there is no literature on this COMU-based activation of acid **6**.

**Table 5:**
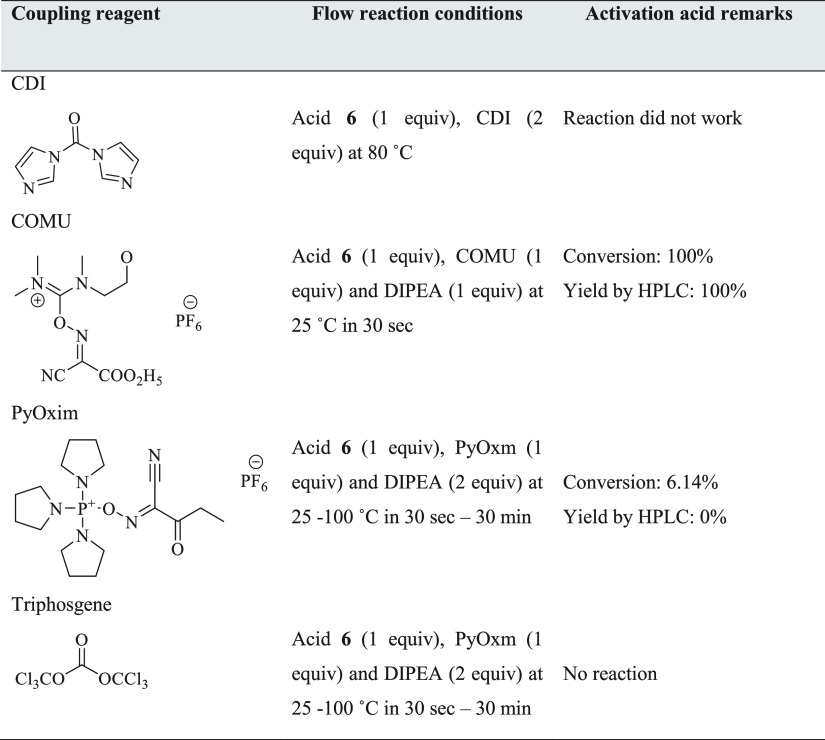
Summary of the Reactivity (Yield) of Different Coupling Reagents on the Formation of Activated Acid in Continuous Flow Systems

### Amidation of Tricycle Intermediate **6** via Activated Acid in Continuous Flow

After having successfully optimized the preactivation process of acid **6** in excellent yields and time using COMU in continuous flow, the next step of the reaction was to form the corresponding dolutegravir benzylamide **7** by treating the optimized activated carboxylic acid **6b** with 2,4-difluorobenzylamine **10**.

**Figure 13:**
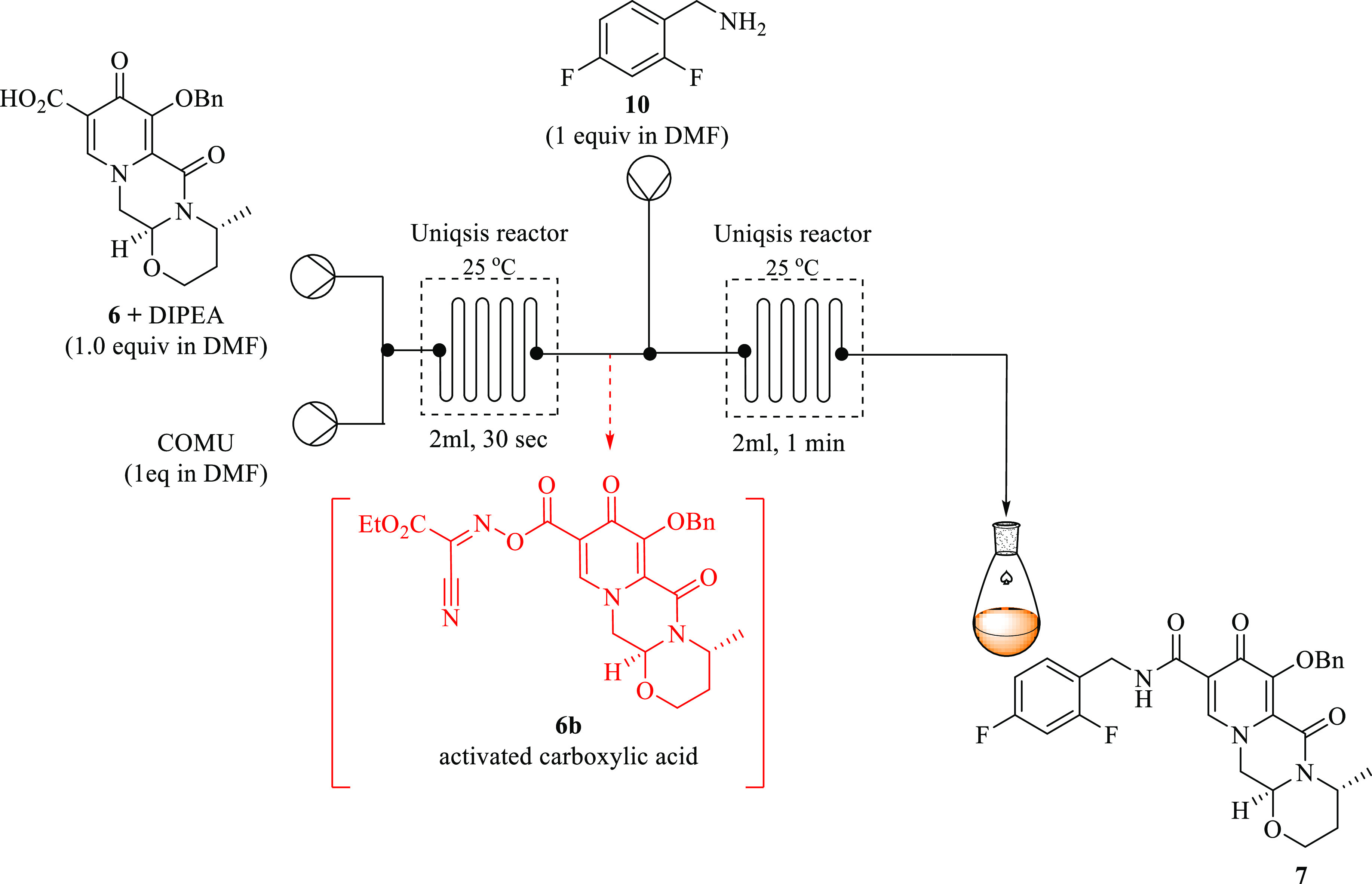
Telescoped tricycle pyridinone acid **6** activation and dolutegravir amide bond **7** formation with COMU in continuous flow systems using DMF at room temperature.

With this knowledge at hand, and having successfully optimized the preactivation step toward **6b**, amidation was then carried out through direct telescoping in the continuous flow process. The amidation was done using two Uniqsis microreactor systems fitted in series, as depicted in Figure 13, via in situ activated acid **6b** formation.

As aforementioned, the optimum conditions for activated carboxylic acid **6b** synthesis were 1 equiv amount of COMU, 1 equiv DIPEA, 30s residence time at room temperature, and full conversion, affording **6b** in 100% yield by HPLC.

Keeping the optimum reaction conditions for the activation step the same without isolation, the aim was to study the amidation process toward the desired **7** by introducing a solution of amine **10** (0.026 M, 1 equiv) in DMF into a channel reactor in half the flow rate of the first reactor at room temperature. The amidation reaction was investigated while keeping the optimum conditions of the preactivation constant. The residence time of reactor one (preactivation step) was kept constant at 30 s while varying the time of reactor 2 (amidation reaction).

After a residence time of 1 min, full conversion was observed and the desired amide **7** was attained at an excellent yield of 100% by HPLC. At a residence time above 1 min, the reaction reached its plateau. However, it was difficult to do further investigations at even lower residence times because the system was stalling due to high pressure when pumping the reagents at fast flow rates. Therefore, the amidation process of **6** was successfully achieved in a combined two step at an optimum total residence time of 1.5 min at room temperature in a continuous flow system, affording dolutegravir amide **7** in 100% selectivity with no side products.

An optimized continuous flow process for the synthesis of dolutegravir amide **7** via COMU-based coupling was successfully developed and described with excellent yield (100% by HPLC) and very low residence time at ambient temperature. The remarkable thing about this process is that carrying out this reaction in flow not only reduced the total reaction time to 1.5 min as opposed to the 4.5 h required in traditional batch affording **7** in 66% isolated yield but also the synthesis of activated acid intermediate was achieved in situ. This clearly emphasizes the advantages of flow systems as opposed to the batch process.

### *O*-Debenzylation of Benzyl in Continuous Flow Systems

Continuous flow was finally applied to synthesize the free acid of dolutegravir **1** through *O*-debenzylation of benzyl dolutegravir **7** in the synthetic route. Ziegler et al. demonstrated free dolutegravir synthesis **1** in 89% yield through the demethylation continuous flow process of methyl dolutegravir in 30 min residence time at 100 °C using PTF tubing in the presence of lithium bromide (2.2 equiv),^3^ Phull et al.^12^ also reported a 15 min continuous flow synthesis of **1** from methyl dolutegravir at 60° in the presence of lithium bromide (2 equiv) in 86% yield.^10^ Both of these flow reports, which to the best of our knowledge are the only documented literature of flow on **1** synthesis, employed the demethylation method with Lewis acid lithium bromide in excellent yields. However, these reactions still proved to be slower.

In contrast, in the case of this study, the debenzylation step proceeded smoothly in batch with Brønsted acid TFA (5 equiv) at 38 °C in 2 h using DCM to afford **1** at 90% isolated yield. The feasibility of translating this process into flow with the aim of forming **7** with a shorter residence time while retaining or even improving the higher yield was examined. To date, to the best of our knowledge, there has not been any literature on the synthesis of **1** in continuous flow with TFA as a debenzylation agent.

**Figure 14:**
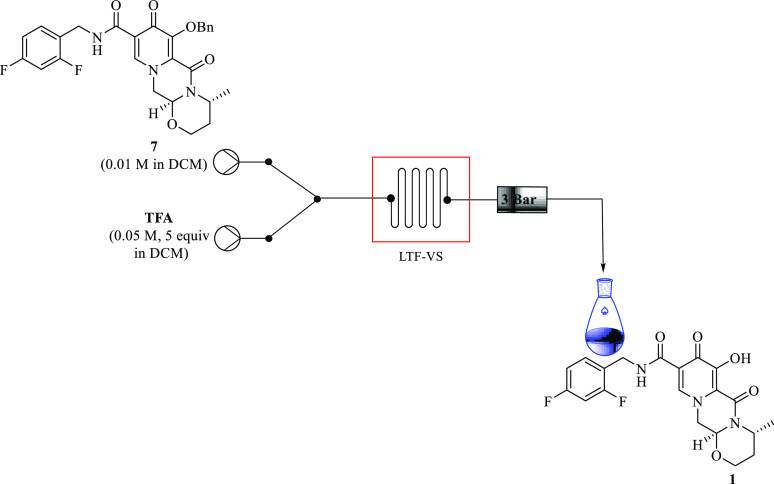
Continuous flow *O*-debenzylation of benzyl dolutegravir **7**.

In the preliminary experiments, benzyl dolutegravir **7** (0.01 M, 1 equiv) was treated with TFA (0.05 M, 5 equiv) in an LTF flow reactor using DCM at 38 °C in 9 min (Figure 14). Interestingly there was no product formed after 9 min. An increase in temperature and residence time resulted in an increase in the conversion of **7**. At most, the desired dolutegravir **1** was attained in 90% yield by HPLC in 30 min residence time at 80 °C (Table 6, Entry 4). When the temperature was raised to 100 °C with the aim of improving the yield, the system was boiling and could not handle the high pressure from the backpressure regulator due to the low boiling temperature of DCM. Using acetonitrile, an alternative solvent that has a higher boiling point beyond 100 °C, afforded **1** in 99% in 9 min residence time at 100 °C (Table 6, Entry 8). When THF and toluene were employed respectively, the reaction did not occur. The effect of conducting this reaction at a lower molar equivalent of TFA using acetonitrile at 120 °C at a constant 9 min residence time was carried out. The results indicated that conducting this reaction at reduced amounts of TFA resulted in a decrease in conversion and yield by a smaller percentage. Dolutegravir **1** was obtained in 96% yield by HPLC at 3 equiv of TFA, while 45% (HPLC) was attained when using 1 equiv amount of TFA (Table 6, entries 9 and 10).

**Table 6:** Condition Screening and Optimization of *O*-Debenzylation Reaction of Benzyl Dolutegravir **7** in Continuous Flow^a^

experiment name	total residence time (min)	solvent	temp (°C)	THF equiv^b^	% yield^c^
1	30	DCM	38	5	No rxn
2	30	DCM	50	5	9
3	15	DCM	80	5	84
4	30	DCM	100	5	90
5	30	ACN	100	5	92
6	3	ACN	100	5	52
7	3	ACN	120	5	93
**8**	**9**	**ACN**	**120**	**5**	**99**
**9**	**9**	**ACN**	**120**	**3**	**96**
10	9	ACN	120	1	44

a Standard conditions: Feed one—benzyl dolutegravir (0.1 M, 1 equiv), feed two—THF.

b Molar equivalence.

c Dolutegravir **1** percentage yield determined by HPLC using a synthetic standard.

A continuous flow method for *O*-debenzylation of **7** in the presence of TFA was successfully presented. The results clearly demonstrated the efficient and rapid use of this methodology. It was possible to synthesize dolutegravir **1** in an overall yield of 96% (by HPLC) at 120 °C in 9 min with reduced TFA (3 equiv) and has a potential to be scaled up. This solved the long reaction time problems when performing this reaction using batch conditions (2 h, 5 equiv TFA at room temperature, 90% isolated yield). Moreover, using flow allowed us to obtain **1** in an even shorter time when using 5 equiv, wherein **1** was attained in 93% in 3 min or 96% in 5 min. This attractive procedure can therefore be used to synthesize this important last intermediate (dolutegravir **1**) in the alternative route.

## Conclusions

We successfully developed an efficient continuous flow procedure toward dolutegravir **1** over 6 six reaction steps from a commercially available starting material benzyl-protected pyran. To the best of our knowledge, there is no literature on the continuous flow synthesis of this drug from pyran. The overall total residence time was 14.5 min with yields of each step between 71 and 100% by HPLC which was an improvement compared to the 35 h batch process, yielding intermediates in lower isolated yields (least being 33%). The most significant feature of this study is the amidation reaction, wherein a novel continuous flow amidation method using coupling reagents is successfully presented. The available literature-reported route employed the formation of amide via mixed carbonic acid anhydrides, wherein carboxylic acid was reacted with ethylchloroformate in the presence of *N*-methylmorpholine as a base. It is to acknowledge that this reported flow procedure was a success; however, in the case of this research, use of ethylchloroformate was not employed. The use of a library of coupling reagents where dolutegravir benzylated amide intermediate **7** was achieved under optimum conditions using COMU in 1.5 min total residence time at 25 °C using Uniqsis microreactor was successfully demonstrated. Another key feature of this procedure is the significant selective monohydrolysis of diester **3** to afford pyridinone-acid **4**. It was demonstrated that the synthesis of this intermediate can be achieved under optimum conditions of 100 °C in 1 min residence time when using KOH and methanol–water cosolvent. Importantly, this compound was also achieved in a multistep synthesis from pyran **3** via the formation of pyridinone **4** using a cheaper and greener base KOH. Under optimum conditions of 20 min total residence time, 100 °C, the desired **4** was attained in 85%. Arguably, this is a relatively longer time; however, this is such a great improvement compared to the very long reaction times and lower yields when these steps were done in batch reactively. This further highlighted the advantage of the continuous flow technology on the development of multistep processes where possible without necessary reoptimization. Moreover, the general adaptability of the developed flow synthesis to form the dolutegravir analogue, cabotegravir, is studied. After some fine studies, this procedure can be used as an alternative approach which will have ramifications for the procurement of this on-demand antiretroviral drug globally. This procedure can potentially be adapted for the synthesis of dolutegravir analogues, cabotegravir and bictegravir, respectively.

## Experimental Procedures

### General Information

All reagents were purchased from commercial suppliers and used without any further purification. Reaction progress and products were monitored, characterized, and analyzed using different analytical techniques. These include FTIR spectroscopy, NMR spectroscopy, thin layer chromatography (TLC), and HPLC. TLC was performed using precoated Merck Kieselgel 60 HF_254_ aluminum-backed TLC plates using short-wave ultraviolet (UV) light (λ 254 nm) as a visualizing agent. To purify compounds where necessary, column chromatography was performed with silica gel 60 using a solvent mixture of hexane and ethyl acetate as a mobile phase. NMR spectra were recorded using a Bruker spectrometer (Bruker Ultrashield TM 400 plus) which was operated at 400 MHz for ^1^H (proton), 100 MHz for ^13^C (carbon), and 376 MHz for ^19^F. Deuterated chloroform (CDCl_3_) or deuterated dimethyl sulfoxide (DMSO-*d*_6_) were used to record the spectra at ambient temperature. All chemical shifts (δ) are reported in parts per million (ppm) downfield from tetramethylsilane. Infrared spectra were recorded on a Bruker Platinum Tensor 27 spectrophotometer with an ATR fitting. The analyses of samples were recorded in the range 4000–400 cm^–1^, and the peaks are reported in wavenumbers (cm^–1^). The HPLC analysis of compounds was carried out using three methods.

### Procedure 1: Continuous Flow Synthesis of Pyridinone Using LTF Microreactor Systems

A solution of dimethyl 3-(benzyloxy)-4-oxo-4*H*-pyran-2,5-dicarboxylate **2** (0.03 M, 1 equiv) in methanol in a syringe and aminoacetaldehyde dimethyl acetal **8** (0.036 M, 1.2 equiv) in methanol in another syringe were taken, and the two were pumped into an LTF–MS reactor for mixing. The reactor output was streamed into an LTF–MS reactor with the solution of *N’N*′-diisopropylethylamine (DIPEA) (0.03 M, 1 equiv) in methanol pumped at half the total flow rate of the first microreactor. The reaction mixture then flowed into two additional LTF-V microreactor residence plates to allow mixing with a BPR cartridge set at 3 bar and the reaction output going directly to an HPLC vial for analysis. Sample quenching was not necessary after collection; samples were analyzed immediately by HPLC (1.1.1). The prepared batch pyridinone **3** and the characterized standard (2.1)^8^ were used to confirm pyridinone **3** from the continuous flow reaction. Yellow viscous oil (7.6 g, 86% yield. ^1^H NMR (400 MHz, CDCl_3_) δ 8.08 (s, 1H), 7.34 (d, *J* = 7.5 Hz, 2H), 7.25 (q, *J* = 7.9 Hz, 3H), 5.19 (s, 2H), 4.38 (t, *J* = 4.5 Hz, 1H), 3.85 (d, *J* = 4.6 Hz, 2H), 3.83 (s, 3H), 3.72 (s, 3H), 3.29 (s, 6H). ^13^C{^1^H} NMR (100 MHz, CDCl_3_) δ: 171.2, 165.4, 162.2, 149.2, 146.2, 136.9, 134.1, 128.8, 128.3, 128.1, 118.2, 102.8, 74.2, 56.7, 55.8, 53.1, 52.3. IR (KBr, cm^–1^): 3000, 2854, 2890, 2835, 1723, 1615 and 1598.

### Procedure 2: Continuous Flow Hydrolysis of Pyridinone **3** Using LTF Microreactor Systems

A solution of pyridinone **3** (0.1 M, 1 equiv) and LiOH (0.6 M, 6 equiv) in methanol in 10 mL SGE glass syringes, respectively, was pumped at equal flow rates into a LTF-MS microreactor, and the output was streamed into an LTF-VS residence microreactor plate fitted with a 5 bar backpressure regulator. At the outlet, the samples were collected, quenched with aqueous HCl, and analyzed by HPLC (1.1.2). The prepared batch acid **4** and characterized standard (2.2)^11^ were used to confirm acid **4** from the continuous flow reaction.

White solid, 1.7 g, 65% yield. ^1^H NMR (400 MHz, CDCl_3_) δ 14.99 (s, 1H), 8.35 (s, 1H), 7.37–7.13 (m, 5H), 5.24 (s, 3H), 4.42 (t, *J* = 2.0 Hz, 1H), 4.02 (d, 4H), 3.76 (s, 4H), 3.30 (s, 8H). ^13^C{^1^H} NMR (100 MHz, CDCl_3_) δ: 174.9, 165.9, 161.4, 147.5, 145.3, 136.6, 136.2, 128.7, 128.5, 128.5, 116.5, 102.3, 74.7, 57.4, 55.9, 53.5. IR (KBr, cm^–1^): 2954, 2985, 2834, 1715, 1616, 1528, 1068.

### Procedure 3: Continuous Flow Hydrolysis of Pyridinone **3** Using PTFE Tubing Microreactor Systems

Using two 10 mL SGE glass reactors, a solution of pyridinone **3** (0.1 M, 1 equiv) in MeOH in a syringe and LiOH (0.6 M, 6 equiv) in MeOH on a second syringe were pumped at equal flow rates through a T-mixer into a PTFE tubing reactor dipped in an oil bath at various temperatures. The PTFE tubing reactor was fitted with a 5 bar backpressure regulator at varying higher temperature conditions, and the progression of the reaction on product **4** samples which were quenched using aqueous HCl was monitored using HPLC (1.1.2).

### Procedure 4: Multistep Syntheses of Pyridinone Acid **4** from Pyran 2 in Continuous Flow Systems

Pyran **2** (0.03 M, 1 equiv) in MeOH was treated with amine **8** (0.036 M, 1.2 equiv) in MeOH in a PTFE tubing reactor at the same flow rate, and KOH solution (0.12 M, 4 equiv) in MeOH was added at the outlet by pumping the solution at half the total flow rate of the first reactor, forming pyridinone **3** in situ. The pyridinone **3** formed in situ was converted to the acid **4** intermediate by allowing the reaction to run longer in another PTFE tubing reactor directly connected into the LTF reactor. To regulate the pressure of the system, a 3 bar backpressure regulator was fitted in the system, and the product **4** samples were collected into a vial, quenched with aqueous HCl placed in the collection flask, and then analyzed using HPLC (1.1.2).

### Procedure 5: Acetal Deprotection Reaction in Continuous Flow Systems

A solution of intermediate **4** (0.01 M) in acetonitrile and neat formic acid was prepared using two separate 10 mL SGE glass syringes at equal flow rates through a LTF-VS microreactor fitted with a 5 bar backpressure regulator. The reaction output was quenched with concentrated NaHCO_3_ solution placed in the collection flask; the samples were collected and then analyzed using HPLC (1.1.2). The prepared batch aldehyde **5** and characterized standard (2.3)^32^ were used to confirm aldehyde **5** from the continuous flow reaction.

White solid, 1.7 g, 65% yield. ^1^H NMR (400 MHz, DMSO-*d*_6_) δ: 15.19 (s, 1H), 8.87 (s, 1H), 7.48 (d, *J* = 6.6 Hz, 2H), 7.38 (q, *J* = 10.0, 8.3 Hz, 3H), 5.76 (s, 1H), 5.24 (d, *J* = 10.9 Hz, 1H), 4.65 (s, 2H), 3.44 (s, 3H). ^13^C{^1^H} NMR (100 MHz, DMSO-*d*_6_) δ: 190.7, 175.5, 165.4, 164.9, 158.0, 154.7, 144.3, 136.7, 129.4, 129.0, 128.7, 115.9, 99.0, 74.4, 57.1, 53.3.

### Procedure 6: Continuous Flow Synthesis of Trycyclic Intermediate **6**

The flow setup assembled consisted of two sets; thus, the initial pumping of a solution of **4** (0.01 M) in acetonitrile and formic acid (15 M) in separate syringes was introduced into a LTF-VS reactor to give aldehyde **5**. Without isolation, the reaction mixture was further pumped into a second LTF-VS reactor and cyclized with the addition of a solution of 3-*R*-aminobutan-1-ol **9** (0.014 M, 1.42 equiv) at half the total flow rate of the first microreactor through a T-mixer. The reaction was allowed to run at thermo-controlled conditions with a Zaiput backpressure regulator fitted at the outlet. Pretreatment of the reaction samples after collection was not necessary. The samples were collected at the outlet of the continuous system and analyzed using HPLC (1.1.3). The prepared batch tricyclic intermediate **6** and characterized standard (2.4)^32^ were used to confirm the tricyclic intermediate **6** from the continuous flow reaction.

Orange solid, 0.7 g, 66% yield. ^1^H NMR (400 MHz, DMSO-*d*_6_) δ 15.54 (s, 1H), 8.79 (s, 1H), 7.58 (d, *J* = 6.2 Hz, 2H), 7.39 (d, *J* = 7.8 Hz, 2H), 5.39 (s, 1H), 5.14 (s, 2H), 4.84–4.75 (m, 2H), 4.65 (d, *J* = 13.6 Hz, 1H), 4.49–4.42 (m, 1H), 3.98 (t, *J* = 11.9 Hz, 1H), 3.87 (d, *J* = 9.5 Hz, 1H), 1.96 (t, 1H), 1.53 (d, *J* = 13.7 Hz, 1H), 1.29 (d, *J* = 5.7 Hz, 3H). ^13^C{^1^H} NMR (100 MHz, DMSO-*d*_6_) δ: 176.2, 165.7, 155.2, 151.0, 144.2, 137.4, 132.7, 128.9, 128.6, 128.5, 115.4, 76.1, 74.42, 62.3, 53.1, 45.1, 29.7, 16.2. IR (KBr, cm^–1^): 2940, 2878, 1730, 1678, 1442, 1304.

### Procedure 7: Continuous Flow Acid Activation Using a Uniqsis Glass Reactor

Acid **6** (0.0256 M, 1 equiv) was premixed with DIPEA (0.05 M, 2 equiv) in anhydrous DMF in a 10 mL SGE glass syringe and pumped through a T-mixer with a coupling reagent (0.05 M, 2 equiv) from a different 10 SGE syringe, both at equal flow rates, and then allowed to mix into a Uniqsis reactor at room temperature. The reaction output pretreatment was not necessary after collection. The samples were collected and analyzed using HPLC (1.1.3).

### Procedure 8: Continuous Flow Synthesis of Amide **7** via Acid **6**

A premixed solution of acid **6** (0.0256 M, 1 equiv) and DIPEA (0.025 M, 1 equiv) in anhydrous DMF was pumped and allowed to mix with COMU (0.025 M, 1 equiv) from a separate syringe, with a molar amount obtained after optimization. The reaction mixture was further introduced into a second Uniqsis reactor without isolation and coupled with amine **10** (0.025 M, 1 equiv) delivered from a separate syringe at half the total flow rate of the first reactor and allowed to react. The product samples were collected and analyzed directly using HPLC (1.1.3). The prepared batch tricyclic amide **7** and characterized standard (2.5)^32^ were used to confirm amide **7** from the continuous flow reaction.

Orange solid, 0.3 g, 33% yield.^1^H NMR (400 MHz, CDCl_3_) δ 10.4 (t, *J* = 6 Hz, 1H), 8.3 (s, 1H), 7.6 (d, *J* = 7.2 Hz, 2H), 7.2–7.4 (m, 4H), 6.8 (m, 2H), 5.2–5.3 (dd, *J* = 16.4 and 10 Hz, 2H), 5.1 (m, 1H), 4.9–5.0 (m, 1H), 4.6 (d, *J* = 6 Hz, 2H), 4.2 (dd, *J* = 13.2 and 3.2 Hz, 1H), 4.0–4.1 (dd, *J* = 13.2 Hz and *J* = 6 Hz, 1H), 3.9 (m, 2H), 2.1–2.2 (m, 1H), 1.4–1.5 (dd, *J* = 14 Hz and *J* = 2 Hz, 1H), 1.3 (d, *J* = 6.8 Hz, 3H). ^13^C{^1^H} NMR (100 MHz, CDCl_3_) δ: 174.5, 164.0, 160.9, 161.0, 163.4, 163.5, 159.4, 159.5, 161.9, 162.0, 155.5, 153.2, 142.0, 136.6, 130.5, 130.6, 129.4, 128.9, 128.2, 128.1, 121.3, 121.5, 118.7, 111.0, 111.2, 103.5, 104.4, 76.0, 74.4, 62.5, 53.5, 44.5, 36.4, 36.5, 29.3, 15.9. ^19^F NMR (376 MHz, CDCl_3_) δ: −111.58 (p, *J* = 7.3 Hz), −114.29 (q, *J* = 8.2 Hz). IR (KBr, cm^–1^): 3062, 2921, 1728, 1651, 1605, 1542, 1084.

### Procedure 9: Continuous Flow *O*-Debenzylation Reaction of Benzyl Dolutegravir **7**

A solution of benzyl dolutegravir **7** (0.01 M, 1 equiv) in DCM and trifluoroacetic acid (0.05 M, 5 equiv) in DCM in 10 mL SGE syringes, respectively, was pumped at equal flow rates through a T-mixer into an LTF-VS microreactor fitted with a 3 bar backpressure regulator to allow superheating. The resultant product samples were collected, quenched with aqueous ammonia, and analyzed using HPLC (1.1.3). The prepared batch dolutegravir **1** and characterized standard (2.6)^8^ were used to confirm dolutegravir **1** from the continuous flow reaction.

White solid, 0.7 g, 90% yield. ^1^H NMR (400 MHz, CDCl_3_) δ 12.4 (s, 1H), 10.3 (t, *J* = 5.6 Hz), 8.3 (s, 1H), 7.3 (m, 1H), 6.7–6.8 (m, 2H), 5.2 (m, 1H), 4.9–5.0 (m, 1H), 4.6 (d, *J* = 6.0 Hz, 2H), 4.2–4.3 (dd, *J* = 13.2 and 4 Hz, 1H), 4.1 (dd, *J* = 13.2 and 6 Hz), 4.0 (m, 2H), 2.1–2.2 (m, 1H), 1.5 (dd, *J* = 14 and 2.0 Hz, 1H), 1.3–1.4 (d, *J* = 7.2 Hz, 3H). ^13^C{^1^H} NMR (100 MHz, CDCl_3_) δ: 171.3, 164.1, 162.5, 160.9–161.0, 163.3–163.4, 159.4, 159.5, 161.9, 162.0, 156.0, 140.1, 130.2, 130.4, 121.4, 121.6, 116.6, 115.8, 111.0, 111.2, 103.4, 103.9, 76.3, 62.7, 52.4, 44.7, 36.5, 36.5, 29.2, 15.5. IR (KBr, cm^–1^): 3341, 3061, 2960, 2942, 2879, 1672, 1607, 1542, 1080.

## Data Availability

The data underlying this study are available in the published article and its Supporting Information.

## Supporting Information Available

The Supporting Information is available free of charge Additional experimental details, materials, methods, and continuous flow system setup and FTIR, ^1^H NMR, and ^13^C NMR spectra for all compounds (PDF).
