# Supplementary material for: Synthesis of Dolutegravir Exploiting Continuous Flow Chemistry
Source: J Org Chem. 2023 Aug 8;88(16):12024–40. doi: 10.1021/acs.joc.3c01365 (PMC10442919; doi:10.1021/acs.joc.3c01365)
Supplement: Supplementary file 1 — jo3c01365_si_001.pdf [file jo3c01365_si_001.pdf]

Supporting Information

- Batch synthesis of compounds **1**, **2**, **3**, **4**, **5**, **6** and **7** are included herein
- Continuous flow set ups for the synthesis of compounds **1**, **2**, **3**, **4**, **5**, **6** and **7** are included herein
- Copies of 1H and 13C{1H} NMR spectra for all compounds (**1**, **2**, **3**, **4**, **5**, **6** and **7**) are included herein
- Copies of FTIR spectra of compounds **1**, **2**, **3**, **4**, **5**, **6** and **7** are included herein

The synthesis of Dolutegravir exploiting continuous flow chemistry

Sinazo Nqeketo and Paul Watts*

Nelson Mandela University, University Way, Port Elizabeth, 6031, South Africa.
Email: Paul.Watts@mandela.ac.za

Table of Contents

1: General Experimental details S3

1.1 Analytical methodology S3

1.1.1 HPLC method 1 .... S3

1.1.2 HPLC method 2 .... S3

1.1.3 HPLC method 3 .... S4

2: Detalis batch procedures towards synthesis of Dolutegravir S4

2.1 3-(benzyloxy)-1-(2,2-dimethoxyethyl)-2,5-bis(methylperoxy)pyridin-4(1*H*)-one 3 .... S4

2.2 3-(benzyloxy)-1-(2,2-dimethoxyethyl)-2,5-bis(methylperoxy)pyridin-4(1*H*)-one 4 .... S4

2.3 3-(benzyloxy)-1-(2,2-dimethoxyethyl)-5-hydroperoxy-2-(methylperoxy)pyridin-4(1*H*)-one 5 .... S5

2.4 5-(benzyloxy)-6-(methoxycarbonyl)-4-oxo-1-(2-oxoethyl)-1,4-dihydropyridine-3-carboxylic acid 6 .... S5

2.5 (4R,12aS)-7-(benzyloxy)-N-(2,4-difluorobenzyl)-4-methyl-6,8-dioxo-3,4,6,8,12,12a-hexahydro-2H-pyrido[1',2':4,5]pyrazino[2,1-b][1,3]oxazine-9-carboxamide 7 .... S5

2.6 (4R,12aS)-N-(2,4-difluorobenzyl)-7-hydroxy-4-methyl-6,8-dioxo-3,4,6,8,12,12a-hexahydro-2H-pyrido-[1’2’:4,5]pyrazino[2,1-b][1,3]oxazine-9-carboxamide 1 .... S6

3: Continuous flow synthetic systems S6

3.1 Details of Flow Equipment used S6

3.2 Continuous flow synthesis of pyridinone using LTF microreactor systems S6

3.3 Monoester hydrolysis of pyridinone in continuous flow S7

3.3.1 Continuous flow hydrolysis of pyridinone 3 using LTF microreactor systems S7

3.3.2 Continuous flow hydrolysis of pyridinone 3 using PTFE tubing microreactor systems 8

3.4 Multistep syntheses of pyridinone acid 4 from pyran 2 in continuous flow systems S9

3.4.1 Multistep continuous flow synthesis of acid 4 using a combination of LTF and PTFE tubing reactors S9

3.4.2 Multistep continuous flow synthesis of acid 4 in PTFE tubing reactor system S10

3.5 Acetal deprotection reaction in continuous flow systems S11

3.5.1 Continuous acetal deprotection in LTF microreactor system S12

3.5.2 Continuous acetal deprotection in Uniqsis packed bed system S12

3.6 Continuous flow synthesis of trycyclic intermediate 6 .... S13

3.7 Amidation reaction of compound 115 in continuous flow systems S14

3.7.1 Continuous flow acid activation using Uniqsis glass reactor S14

3.7.2 Continuous flow synthesis of amide 7 *via* acid 6 .... S15

3.8 Continuous flow *O*-debenzylation reaction of benzyl dolutegravir 7 .... S16

4: NMR and FTIR of all compounds synthesised in the optimised synthesis of Dolutegravir ... S18

1: General Experimental details

1.1 Analytical methodology

All reagents were purchased from commercial suppliers and were used without any further purification. Reaction progress and products were monitored, characterised and analysed using different analytical techniques. These include Fourier-transform infrared spectroscopy (FTIR), Nuclear Magnetic Resonance (NMR) spectroscopy, thin layer chromatography (TLC) and High-Performance Liquid Chromatography (HPLC). TLC was performed using precoated Merck Kieselgel 60 HF_{254} aluminium backed TLC plates using short wave ultra violet (UV) light (λ 254 nm) as a visualizing agent. To purify compounds where necessary, column chromatography was performed with Silica Gel 60 using a solvent mixture of hexane and ethyl acetate as a mobile phase. Nuclear magnetic resonance (NMR) spectra were recorded using Bruker spectrometer (Bruker Ultrashield TM 400 plus) which was operated at 400 MHz for ^{1}H (proton), 100 MHz for ^{13}C (carbon) and 376 MHz for ^{19}F. Deuterated chloroform ($CDCl_3$) or deuterated dimethyl sulfoxide (DMSO-D_6) were used to record spectra at ambient temperature. All chemical shifts (δ) are reported in parts per million (ppm) downfield from tetramethylsilane. Infrared spectra were recorded on a Bruker Platinum Tensor 27 spectrophotometer with an ATR fitting. The analyses of samples were recorded in the range 4000–400 cm^{-1} and the peaks are reported in wavenumbers (cm^{-1}). The HPLC analysis of compounds was carried out using three methods.

1.1.1 HPLC method 1

The HPLC analysis of compounds was carried out using an Agilent 1100 fitted with an Agilent Zorbax C_{18} (250 x 4.60 mm x 5 microns) column under the following conditions; flow rate: 1.00 ml/min, mobile phase [acetonitrile and water (60:40)] equipped with a variable wavelength detector at ambient temperature as used for sample analysis. The wavelength used for detection was 254nm. The sample injection volume was 1 µl, eluted at a flow rate of 1.0 ml/min with a run time of 7 minutes at ambient temperature.

1.1.2 HPLC method 2

Samples were analysed using an HPLC Agilent 1100 fitted with Agilent Zorbax C_{18} (250 x 4.60 mm x 5 microns) column under the following conditions; flow rate: 1.00 ml/min, mobile phase [acetonitrile and water (60:40)] equipped with a variable wavelength detector at ambient temperature as used for sample analysis. The wavelength used for detection was 210nm. The

sample injection volume was 1 µl, eluted at a flow rate of 1.0 ml/min with a run time of 7 minutes at ambient temperature.

1.1.3 HPLC method 3

The HPLC analysis was performed on an Agilent 1220 using Agilent Zorbax C_{18} (250 x 4.60 mm x 5 microns) column under the following conditions; flow rate: 1.00 ml/min, mobile phase [acetonitrile and water (60:40)] equipped with a variable wavelength detector at ambient temperature as used for sample analysis. The wavelength used for detection was 210nm. The samples injection volume was 0.1 µl, eluted at a flow rate of 1.0 ml/min with a run time of 8 minutes at ambient temperature.

Data analysis

To determine the validity of the data, for every reaction run the samples were collected in triplicates and the average used for reporting.

2: Detalis batch procedures towards synthesis of Dolutegravir

2.1 3-(benzyloxy)-1-(2,2-dimethoxyethyl)-2,5-bis(methylperoxy)pyridin-4(1*H*)-one 3[8]

Dimethyl 3-(benzyloxy)-4-oxo-4*H*-pyran-2,5-dicarboxylate **2** (7.0 g, 21.99 mmol) was dissolved in methanol (70 ml) at room temperature in a round bottom flask. To that solution aminoacetaldehyde dimethyl acetal **8** (2.9 ml, 26.34 mmol) was added dropwise within 2 minutes followed by addition of a base *N'N'*-diisopropylethylamine (3.8 ml, 22.04 mmol). The reaction mixture was stirred at room temperature for 18 hrs. The reaction was quenched with aqueous citric acid (50% solution, 14 ml) after completion until pH 6.0-7.0 was obtained. The solvent was removed under vacuum and the residue was dissolved in dichloromethane (56 ml) and washed sequentially with water (14 ml) with separated organic layer. The remaining organic layer was dried over Na_2SO_4, removed under vacuum and afforded compound **3** (7.6 g, 86%) as a yellow viscous oil. To evade material loss and in view of clean conversion and high yields, the product was used for the proceeding steps without further purification.

2.2 3-(benzyloxy)-1-(2,2-dimethoxyethyl)-2,5-bis(methylperoxy)pyridin-4(1*H*)-one 4[11]

In a typical pyridinone ester base mediated monohydrolysis, pyran **3** (7.0 g, 7.27 mmol) was dissolved in methanol (70 ml). Lithium hydroxide (2.41 g, 0.1 mol) was added and the reaction mixture was stirred at 0 ˚C for 4.5 hrs and the reaction was monitored by TLC. After the reaction completion, the reaction mixture was quenched by an addition of 2.0 M hydrochloric

acid (1.7 ml, 49.07 mmol, 6.75 equiv) and subjected to extraction with ethyl acetate (50 ml). The ethyl acetate layer was washed with water (25 ml), and then dried over anhydrous sodium sulphate. The solvent was distilled off to obtain compound **4** as a pale yellow oil (4.4 g, 64%).

2.3 3-(benzyloxy)-1-(2,2-dimethoxyethyl)-5-hydroperoxy-2-(methylperoxy)pyridin-4(1*H*)-one 5[32]

Acid **4** (3.0 g, 7.67 mmol) was dissolved in 98% formic acid (21.14 ml) and to the resultant solution was added 62% H_2SO_4 (1.15ml) was added and the reaction was stirred at 5 ˚C for 3 hours. An aqueous solution of saturated sodium hydrogen carbonate was added while maintaining the temperature at 5 ˚C and extracted with dichloromethane (40 ml). The remaining organic layer was dried over $MgSO_4$, removed off under vacuum and afforded compound **5** as a white solid (1.7 g, 65%), which was used for the next step without further purification. m.p 210-211 ˚C (lit. N/A).

2.4 5-(benzyloxy)-6-(methoxycarbonyl)-4-oxo-1-(2-oxoethyl)-1,4-dihydropyridine-3-carboxylic acid 6[32]

Aldehyde **5** (1.0 g, 2.9 mmol) was dissolved in toluene (11.1 ml). To this was added methanol (0.45 ml), 3-*R*-aminobutan-1-ol **9** (0.41 ml, 4.11 mmol) and acetic acid (0.23 ml, 5.09 mmol) and stirred at 100 ˚C for 2.5 hours. The reaction mixture was allowed to cool to room temperature then water (5 ml) was added followed by extraction with ethyl acetate (20 ml × 2). The solvent was distilled off to give brown oil. Pure orange solid **6** (0.7 g, 66% yield) was obtained from concentrated mother liquor by column chromatography (silica gel, chloroform/methanol = 97:3 (v/v)). m.p 222-223 ˚C (lit. N/A).

2.5 (4R,12aS)-7-(benzyloxy)-N-(2,4-difluorobenzyl)-4-methyl-6,8-dioxo-3,4,6,8,12,12a-hexahydro-2H-pyrido[1',2':4,5]pyrazino[2,1-b][1,3]oxazine-9-carboxamide 7[32]

To a solution of acid **6** (0.5 g, 1.35 mmol) in anhydrous DMF (15 ml) was added carbonyl diimidazole (0.44 g, 2.71 mmol) under nitrogen atmosphere. The suspension was heated and stirred at 80 ˚C for 2 hours wherein an orange solution was formed. The mixture was allowed to cool to room temperature and 2,4-difluorobenzylamine **10** (0.33 ml, 2.78 mmol) was added dropwise within 2 minutes and stirred at room temperature for 2 hours. The reaction was quenched with water (5 ml) and extracted with DCM (10 ml). The DCM layer was distilled off and the obtained residue was purified by column chromatography (chloroform/methanol = 97:3 (v/v) to afford **7** as a faint orange solid (0.3 g, 33%). m.p 151-152 ˚C (lit. N/A).

2.6 (4R,12aS)-N-(2,4-difluorobenzyl)-7-hydroxy-4-methyl-6,8-dioxo-3,4,6,8,12,12a-hexahydro-2H-pyrido-[1’2’:4,5]pyrazino[2,1-b][1,3]oxazine-9-carboxamide 1[8]

Enol ether (1.0 g, 1.96 mmol) **7** was dissolved in dichloromethane (18 ml) and the solution was heated to reflux in a two neck round bottom flask. To the reaction mixture, a solution of trifluoroacetic acid (0.75 ml, 35.94 mmol) in dichloromethane (2 ml) was added slowly and stirred 2 hrs. After reaction completion, the mixture was cooled to 10-15 ºC and treated with water (4 ml). Aqueous ammonia (0.84 ml) was added to adjust the pH to value 7.0-9.0. Thereafter, the reaction mixture was warmed to room temperature and washed the layer sequentially with water (2 ml), brine (2 ml), and 10% citric acid (4 ml). The organic layer was taken up and distilled under reduced vacuum pressure. The residue was concentrated with toluene and stirred at 60-65 ºC for 1 hour. The precipitate formed was gradually cooled to 10-15 ºC, and dried under vacuum to afford debenzylated off white solid product **1** (0.7 g, 90%). m.p 192-193 ˚C (lit.[1] 192 ˚C).

3: Continuous flow synthetic systems

3.1 Details of Flow Equipment used

All continuous flow procedures investigated in this study were achieved by employing a use of different flow reactores; that is, Little Things Factory (LTF) microreactors, Chemtrix Labtrix Start, Uniqsis packed bed flow column reactors and PTFE coil reactor systems respectively.

3.2 Continuous flow synthesis of pyridinone using LTF microreactor systems

Firstly, to investigate the effectiveness of enabling technology in the amination reaction of pyran **2** in LTF systems, the experiments were conducted using a combination of microreactor plates (two LTF-MS plates and two LTF-V plates) as depicted in Figure S1 and the total volume of the system was 2.98 mL. Three solutions were prepared and were loaded in 10 mL SGE glass syringes respectively. In one syringe, a solution of dimethyl 3-(benzyloxy)-4-oxo-4*H*-pyran-2,5-dicarboxylate **2** (0.03 M, 1 equiv) in methanol and aminoacetaldehyde dimethyl acetal **8** (0.036 M, 1.2 equiv) in methanol in another syringe and the two were pumped into an LTF–MS reactor for mixing. The reactor output was streamed into an LTF–MS reactor with a solution of *N’N*’-diisopropylethylamine (DIPEA) (0.03 M, 1 equiv) in methanol pumped at half the total flow rate of the first microreactor. The reaction mixture then flowed into two additional LTF-V microreactor residence plates to allow mixing with a BPR cartridge set at 3 bar and reaction output going directly to an HPLC vial for analysis. In this experiment, sample

quenching was not necessary after collection, samples were analysed immediately using HPLC method 1.

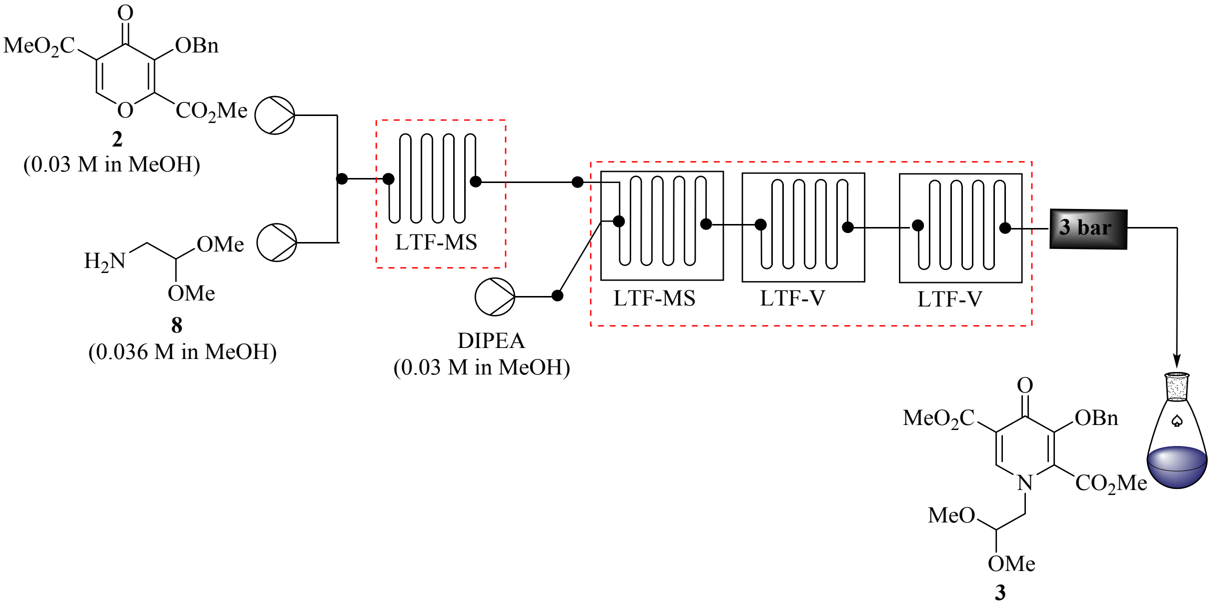

Figure S1: Schematic representation of the LTF microreactor systems used in the synthesis and optimisation pyridinone **3**

3.3 Monoester hydrolysis of pyridinone in continuous flow

3.3.1 Continuous flow hydrolysis of pyridinone 3 using LTF microreactor systems

An LTF microreactor flow system was assembled to perform hydrolysis of pyridinone **3** (Figure S2). A solution of pyridinone **3** (0.1 M, 1 equiv) and LiOH (0.6 M, 6 equiv) in methanol in 10 mL SGE glass syringes respectively was pumped at equal flow rates into a LTF-MS microreactor and the output was streamed into an LTF-VS residence microreactor plate fitted with a 5 bars backpressure regulator. At the outlet, the samples were collected, quenched with aqueous HCl and analysed using HPLC method 2.

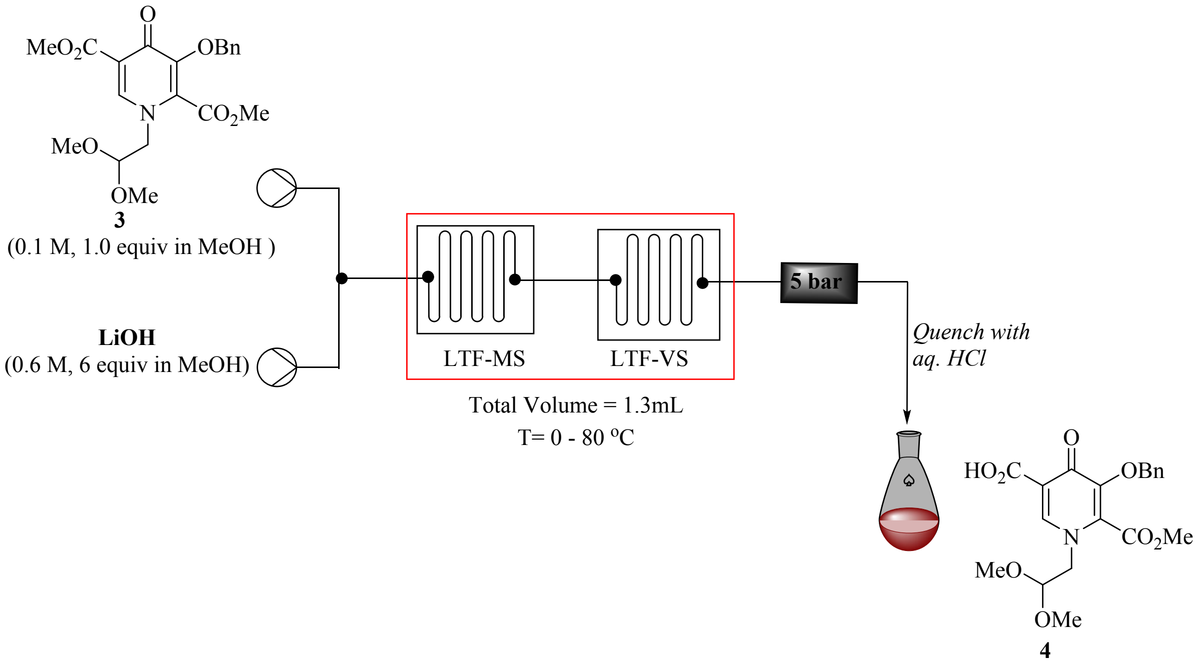

Figure S2: Schematic representation for the synthesis and optimisation pyridinone acid **4** using LTF reactor systems

3.3.2 Continuous flow hydrolysis of pyridinone 3 using PTFE tubing microreactor systems

Selective monohydrolysis of pyridinone was investigated in a 3 ml PTFE tubing reactor system to afford intermediate **4** as depicted in Figure S3.

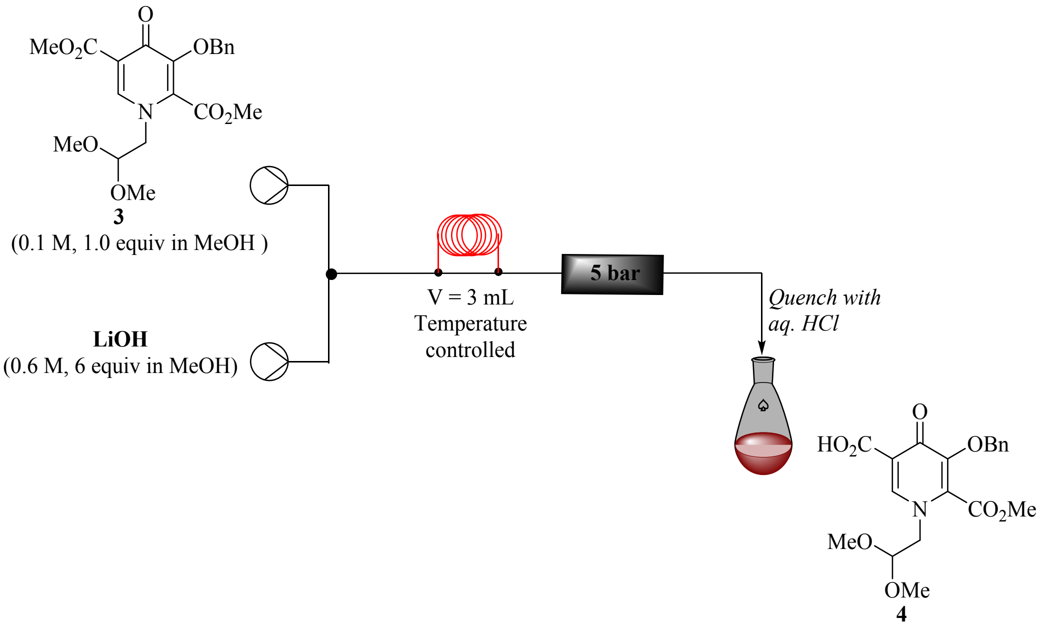

Figure S3: Continuous flow systems of selective monohydrolysis reaction in PTFE tubing

Using two 10 ml SGE glass reactors, a solution of pyridinone **3** (0.1 M, 1 equiv) in MeOH and LiOH (0.6 M, 6 equiv) in MeOH on a second syringe were pumped at equal flow rates through a T-mixer into a PTFE tubing reactor dipped in an oil bath at various temperatures. The PTFE tubing reactor was fitted with a 5 bar backpressure regulator at varying higher temperature conditions and the progression of the reaction on samples which were quenched using aqueous HCl was monitored using HPLC method 2. Noteworthy, for this reaction a series of bases (*N*,*N*-diisopropylethylamine (DIPEA), trimethylamine (TEA), 1,8-diazabicyclo-[5.4.0]undec-7-ene (DBU), imidazole, tributylamine (TBA), trihexylamine (THA), 1,4-diazabicyclo[2.2.2]octane (DABCO), KOH and NaOH) were screened.

3.4 Multistep syntheses of pyridinone acid 4 from pyran 2 in continuous flow systems

The multistep synthesis of acid **4** from pyran **2** *via* pyridinone **3** (Scheme S1) was performed after successfully optimising the synthesis intermediates **3** and **4** in continuous flow systems respectively.

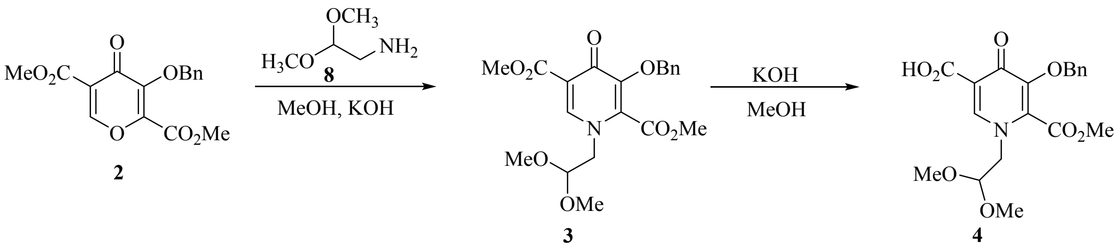

Scheme S1: Multistep synthesis of acid **4**

The optimum reaction condition studies attained were used to investigate the in different flow systems. Both steps consisted of KOH as an optimum base and a total of 4 molar equivalents of KOH was used to conduct telescoped flow investigations for **4** formation from **2** pyran *via* pyridinone **3**.

3.4.1 Multistep continuous flow synthesis of acid 4 using a combination of LTF and PTFE tubing reactors

A combination of one LTF-MS microreactor and PTFE tubing (0.88 mm ID) was using, giving a 3ml total volume of the system (Figure S4).

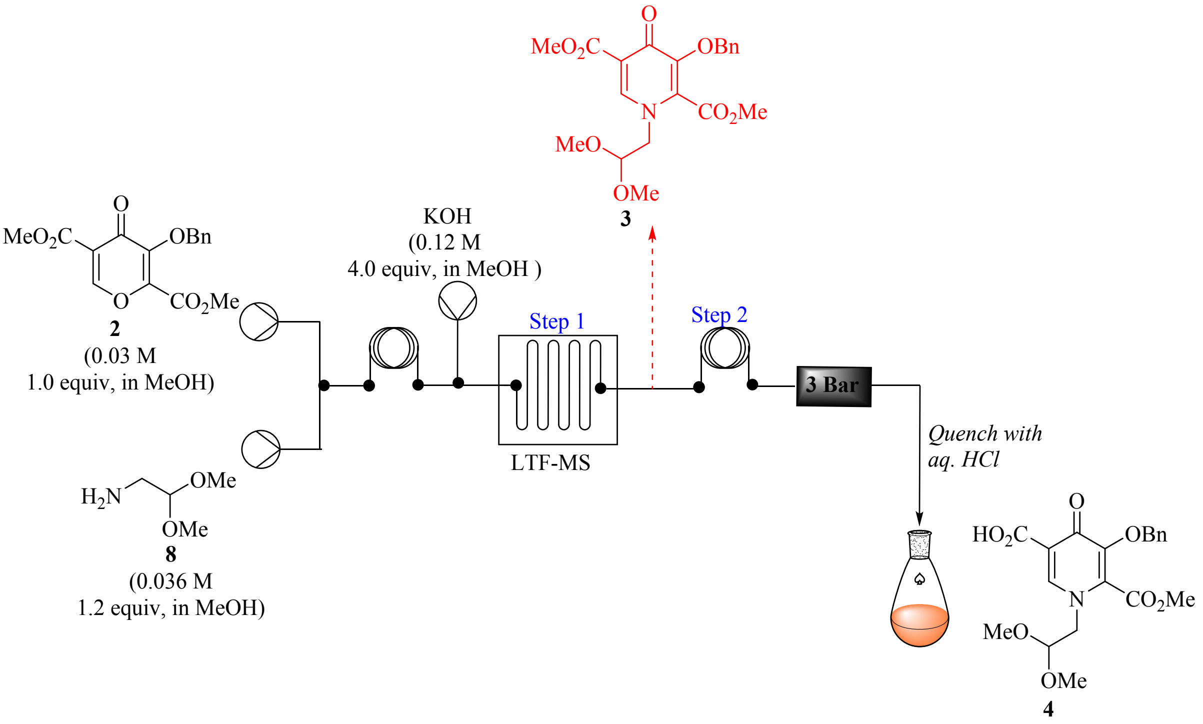

Figure S4: Multistep synthesis of acid **4** in LTF/PTFE tubing continuous flow system

Pyran **2** (0.03 M, 1 equiv) in MeOH was treated with amine **8** (0.036 M, 1.2 equiv) in MeOH in a PTFE tubing reactor at the same flow rate and KOH solution (0.12 M, 4 equiv) in MeOH was added at the outlet by pumping the solution at half the total flow rate of the first reactor forming pyridinone **3** *in situ*. The pyridinone **3** formed *in situ* was converted to acid **4** intermediate by allowing the reaction to run longer in another PTFE tubing reactor directly connected into the LTF reactor. To regulate the pressure of the system; a 3 bar backpressure regulator was fitted in the system and the collected samples into a vial, quenched with aqueous HCl placed in the collection flask, then analysed using HPLC method 2.

3.4.2 Multistep continuous flow synthesis of acid 4 in PTFE tubing reactor system

Multistep synthesis of acid **4** in a PTFE tubing system was accomplished using a 3 ml PTFE tubing reactor fitted with a 3 back pressure to regulate the pressure of the system as illustrated below (Figure S5).

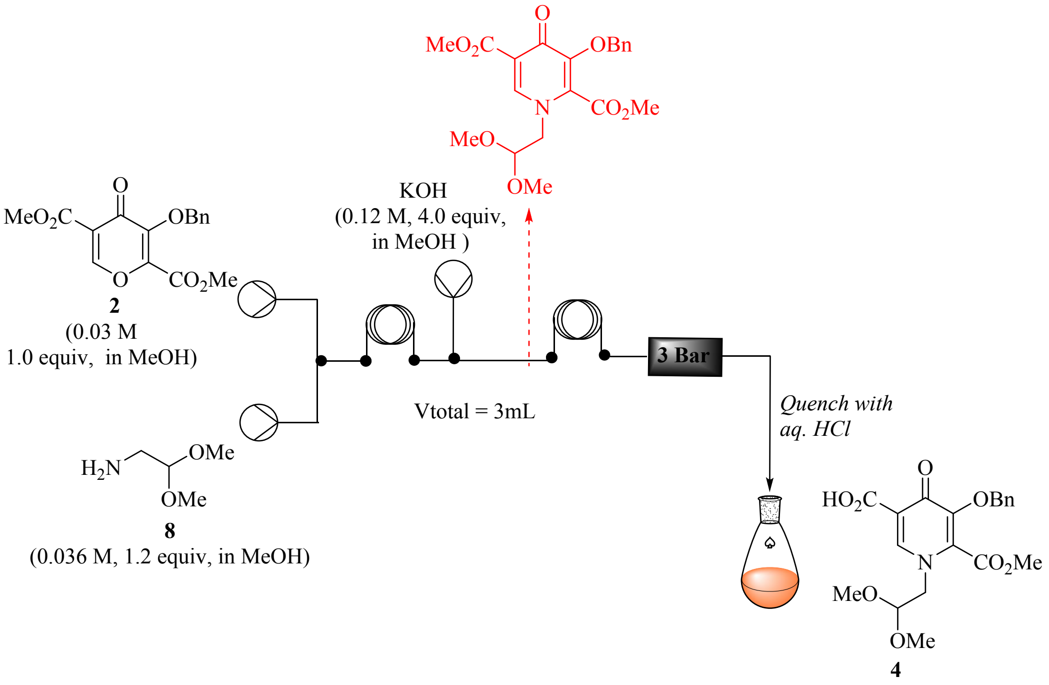

Figure S5: Multistep synthesis of acid **4** in a PTFE tubing continuous flow system

Similarly to the previously described LTF/PTFE tubing system in section 2.7.2.1, the solutions were prepared at the same concentrations (Solution A: Pyran **3** (0.03 M, 1 equiv), Solution B: amine **8** (0.036 M, 1.2 equiv), Solution C: KOH (0.12 M, 4 equiv, pumped at half flow rate)) in methanol and allowed to react in the system. The resultant samples were collected, quenched with aqueous HCl placed in the collection flask, then analysed using HPLC method 2.

3.5 Acetal deprotection reaction in continuous flow systems

Amination deprotection **5** to aldehyde **5** is in the presence of acid is the fourth step of in the synthesis of dolutegravir **1** (Scheme S2). This reaction was conducted in continuous flow, investigated and optimized using LTF microreactor systems and Uniqsis packed bed continuous flow systems respectively.

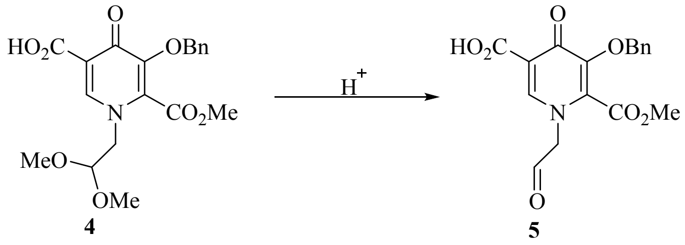

Scheme S2: Acetal deprotection of compound **4**

3.5.1 Continuous acetal deprotection in LTF microreactor system

The continuous system was achieved pumping a solution of intermediate **4** (0.01 M) in acetonitrile and neat formic acid using two separate 10 ml SGE glass syringes at equal flow rates through a LTF-VS microreactor fitted with a 5 bar back pressure regulator (Figure S6). The reaction output was quenched with concentrated $NaHCO_3$ solution placed in the collection flask, then the samples were collected then analysed using HPLC method 2.

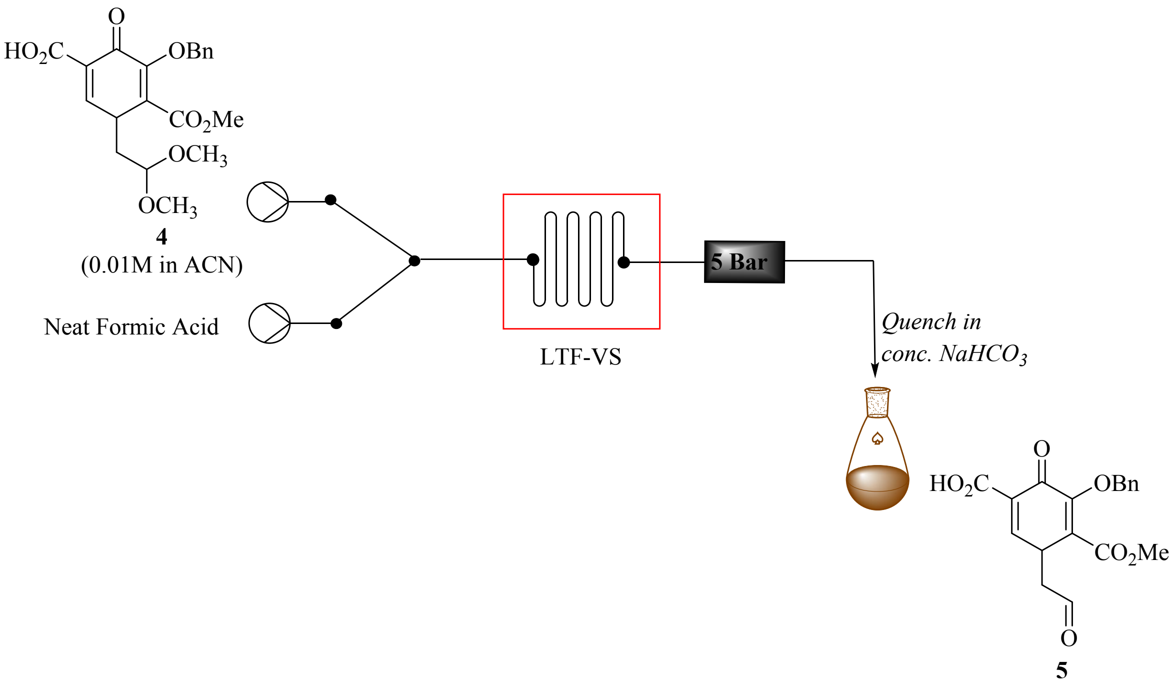

Figure S6: Acetal deprotection of pyridinone acid **4**

3.5.2 Continuous acetal deprotection in Uniqsis packed bed system

A Uniqsis packed bed glass reactor loaded with acidic resin catalysts (Amberlyst-15 (2.6 g), Amberlyst-36 (3.4 g), and Amberlite IR-120 (3.7 g) respectively. The bed length occupied by the catalyst was kept the same for all catalysts (4.8 cm bed height), however because the density is different, the catalysts were packed in different weights.

These resin catalysts occupied a bed height of 6 cm with a pore radius of 0.12 for Amberlyst-15, 0.085 for Amberlyst-36 0.083 Amberlite IRA-93 (Figure S7). A solution of acid **4** (0.01 M) in acetonitrile was pumped into the system fitted with a 3 bar backpressure regulator. The samples were collected, quenched with concentrated $NaHCO_3$ and analysed using HPLC method 2.

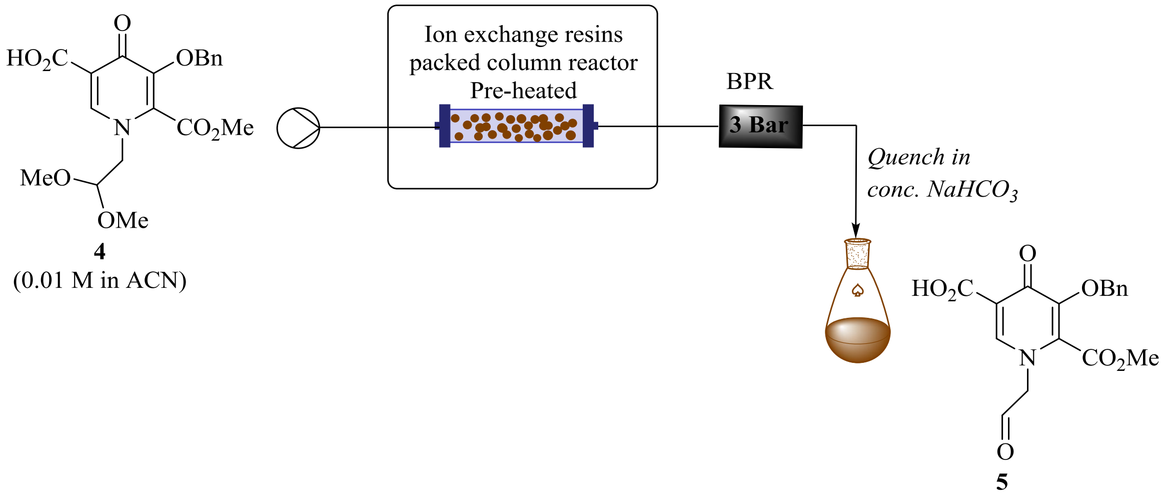

Figure S7: Continuous synthesis and optimisation of aldehyde **4** in a Uniqsis packed bed reactor

3.6 Continuous flow synthesis of trycyclic intermediate 6

Synthesis of intermediate **6** was the next stage of the reaction achieved *via* **5** in continuous flow after having achieved optimization of aldehyde **5** formation separately (Scheme S3). The system was assembled by directly integrating addition of amine **9** for cyclization without isolation.

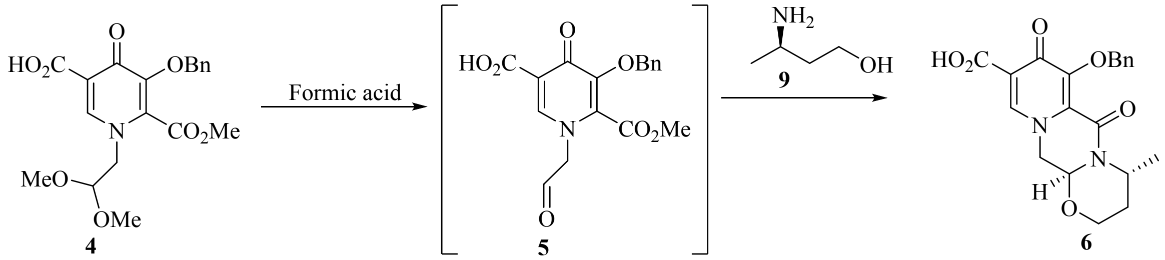

Scheme S3: Synthesis of intermediate **6** from acid **4** *via* aldehyde formed *in situ*

The flow setup assembled consisted of two sets, thus initial pumping a solution of **4** (0.01 M) in acetonitrile and formic acid (15 M) in separate syringes were introduced into a LTF-VS reactor to give aldehyde **5**. Without isolation, the reaction mixture was further pumped into a second LTF-VS reactor and cyclised by with an addition of a solution of 3-*R*-aminobutan-1-ol **9** (0.014 M, 1.42 equiv) at half the total flow rate of the first microreactor through a T-mixer (Figure S8). The reaction was allowed to run at thermo-controlled conditions with a Zaiput back pressure regulator fitted at the outlet. Pre-treatment of the reaction samples after collection was not necessary. The samples were collected at the outlet of the continuous system and analysed using HPLC method 3.

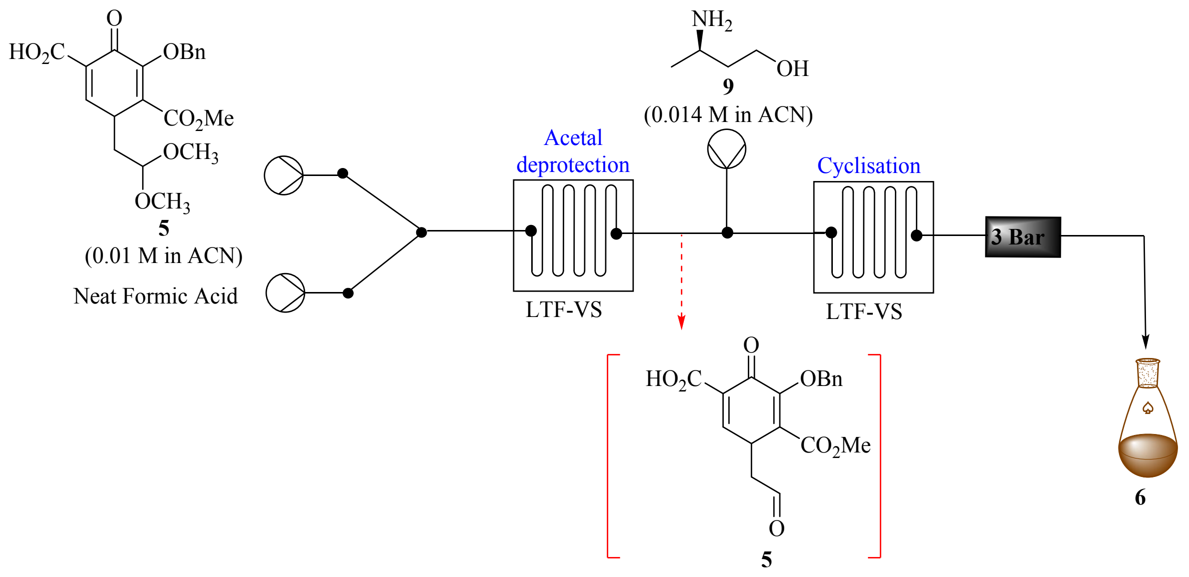

Figure S8: Continuous flow synthesis of intermediate **6** from acid **4** *via* aldehyde **5** formed *in situ*.

3.7 Amidation reaction of compound 115 in continuous flow systems

Amidation of compound **6** (Scheme S4) was optimised in two steps continuous flow process *via* acid pre-activation to form activated acid followed by amide coupling. The pre-activation step was optimized in a single flow Uniqsis system and thereafter coupled with amine **10** in a second Uniqsis without isolation.

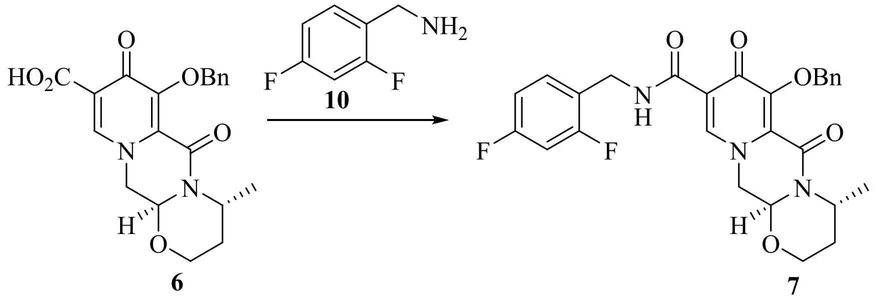

Scheme S4: Amidation reaction of acid **6**

3.7.1 Continuous flow acid activation using Uniqsis glass reactor

To achieve acid **6** pre-activation and optimization, a Uniqsis continuous flow system was assembled as depicted in schematic Figure S9. The pre-activation was conducted using different coupling reagents dissolved in DMF to afford activated species. The following coupling reagents were examined; 1,1'-carbonyldiimidazole (CDI), COMU, triphosgene and PyOxim.

Acid **6** (0.0256 M, 1 equiv) was premixed with DIPEA (0.05 M, 2 equiv) in anhydrous DMF in a 10 ml SGE glass syringe and pumped through a T–mixer with a coupling reagent (0.05 M, 2 equiv) from a different 10 SGE syringe both at equal flow rates then allowed to mix into a Uniqsis reactor at room temperature. The reaction output pre-treatment was not necessary after collection. The samples were collected and analysed using HPLC method 3.

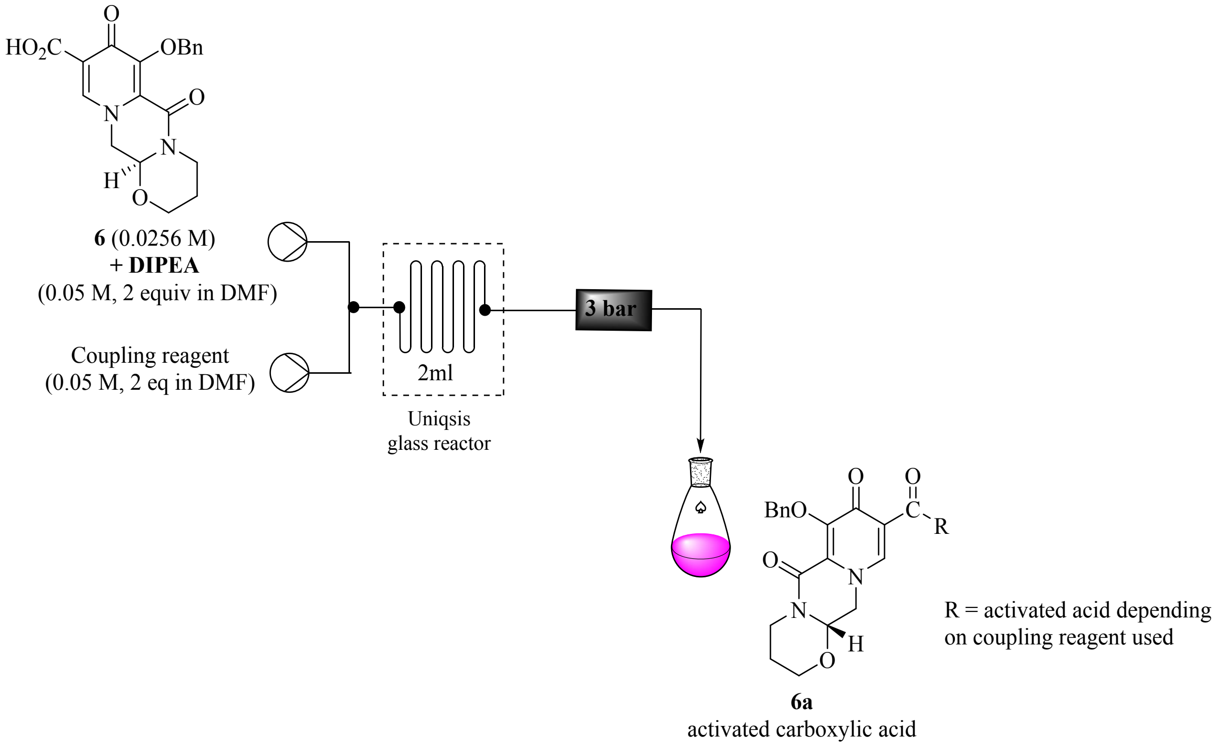

Figure S9: Continuous flow acid **6** pre-activation in a Uniqsis reactor

3.7.2 Continuous flow synthesis of amide 7 *via* acid 6

Amide **7** formation was performed after fully optimizing the pre-activation step with COMU by directly telescoping the amine for amidation and the continuous flow system was assembled as illustrated in Figure S10. The system consisted of two Uniqsis chip reactors (2 ml).

A premixed solution of acid **6** (0.0256 M, 1 equiv) and DIPEA (0.025 M, 1 equiv) in anhydrous DMF was pumped and allowed to mix with COMU (0.025 M, 1 equiv) from a separate syringe, molar amount obtained after optimization. The choice of a coupling reagent and its molar ratio 1:1 of **6** to COMU was based on optimization conditions obtained in the in the above pre-activation step briefly discussed in the results section. The reaction mixture was then further introduced into a second Uniqsis reactor without isolation and coupled with amine **10** (0.025 M, 1 equiv) delivered from a separate syringe at half the total flow rate of the first reactor and

allowed to react. The product samples were collected and analysed directly using HPLC method 3. The reaction output pre-treatment was not necessary after collection.

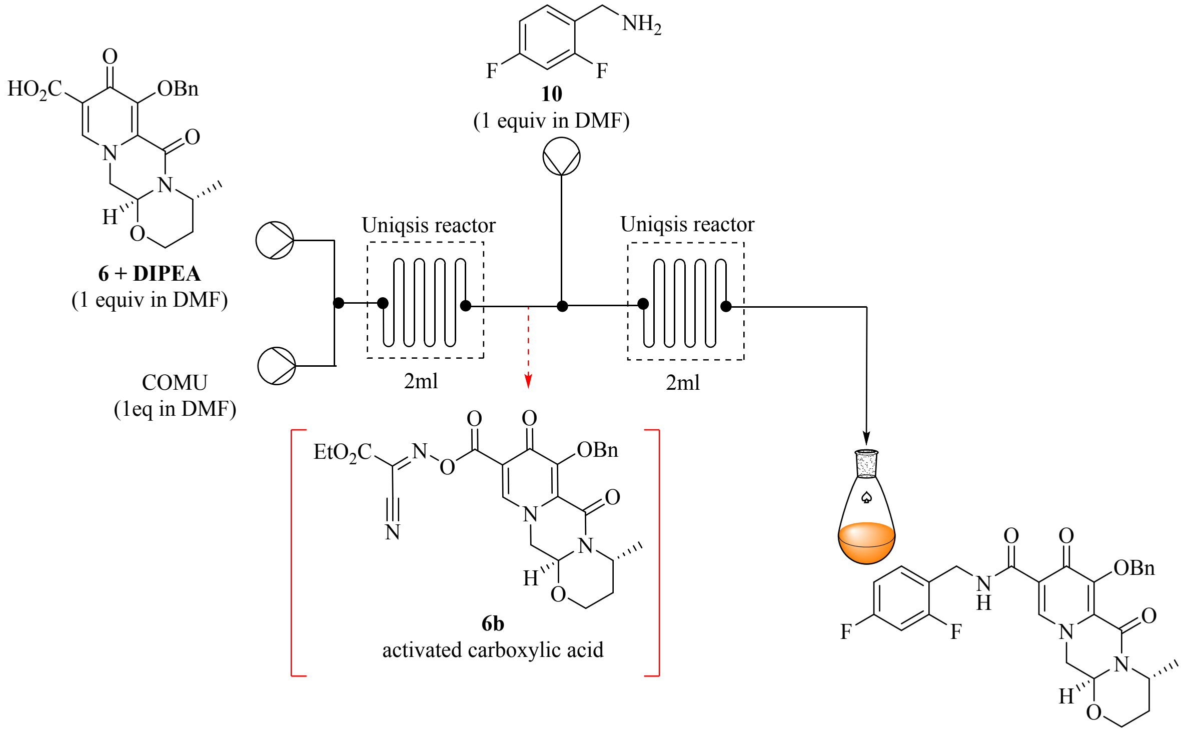

7

Figure S10: Continuous flow amidation of acid **6**

3.8 Continuous flow *O*-debenzylation reaction of benzyl dolutegravir 7

O-Debenzylation of benzyl dolutegravir **7** was the final step of the synthesis (Scheme S5). In continuous flow this reaction was achieved using a 2 ml LTF-VS microreactor systems fitted with a 3 bar Zaiput back pressure regulator as depicted in Figure 11.

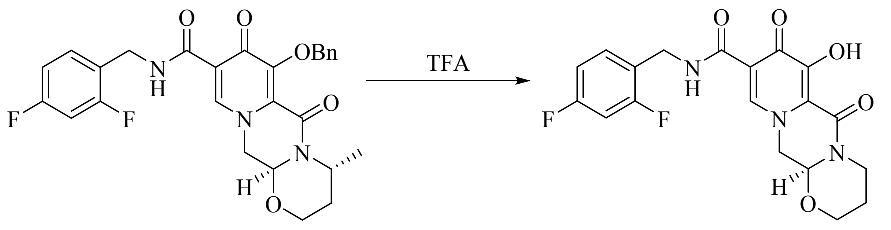

7

1
Dolutegravir

Scheme S5: *O*-Debenzylation of benzyl dolutegravir **7**

A solution of benzyl dolutegravir **7** (0.01 M, 1equiv) in DCM and trifluoroacetic acid (0.05 M, 5 equiv) in DCM in 10 mL SGE syringes respectively was pumped at equal flow rates through a T-mixer into an LTF-VS microreactor fitted with a 3 bar backpressure regulator to allow

superheating. The resultant product samples were collected, quenched with aqueous ammonia and analysed using HPLC method 3.

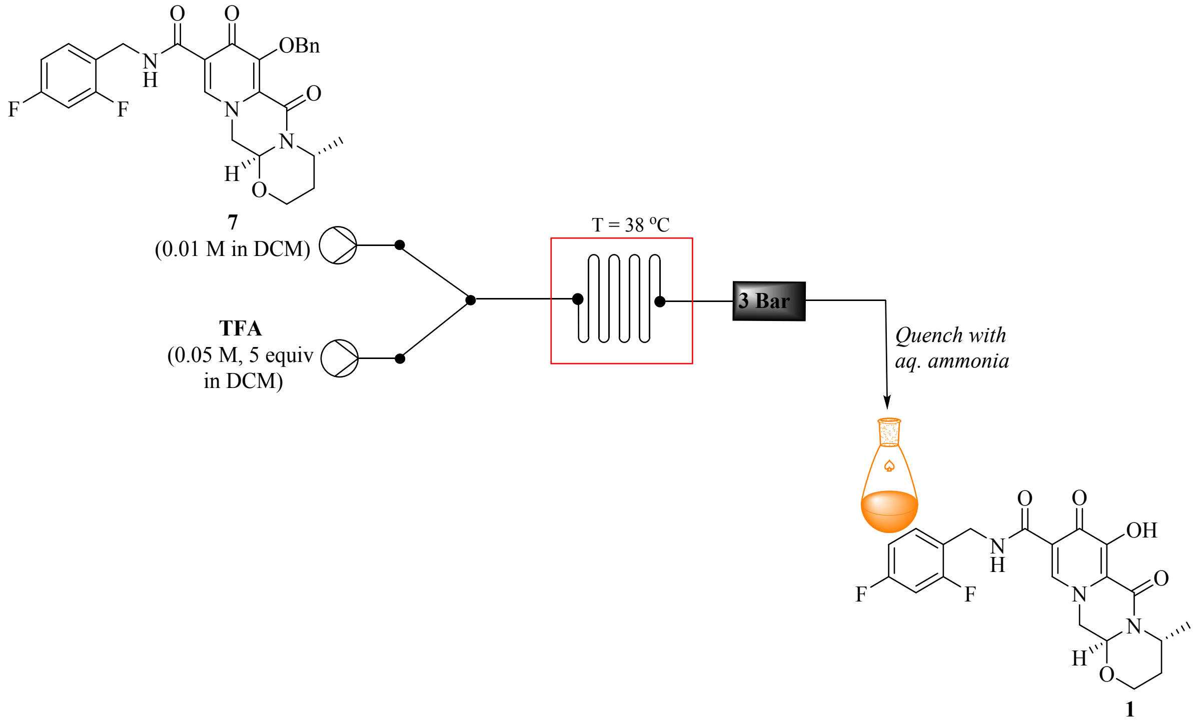

Figure S11: Continuous flow *O*-debenzylation of benzyl dolutegravir **7**

4: NMR and FTIR of all compounds synthesised in the optimised synthesis of Dolutegravir

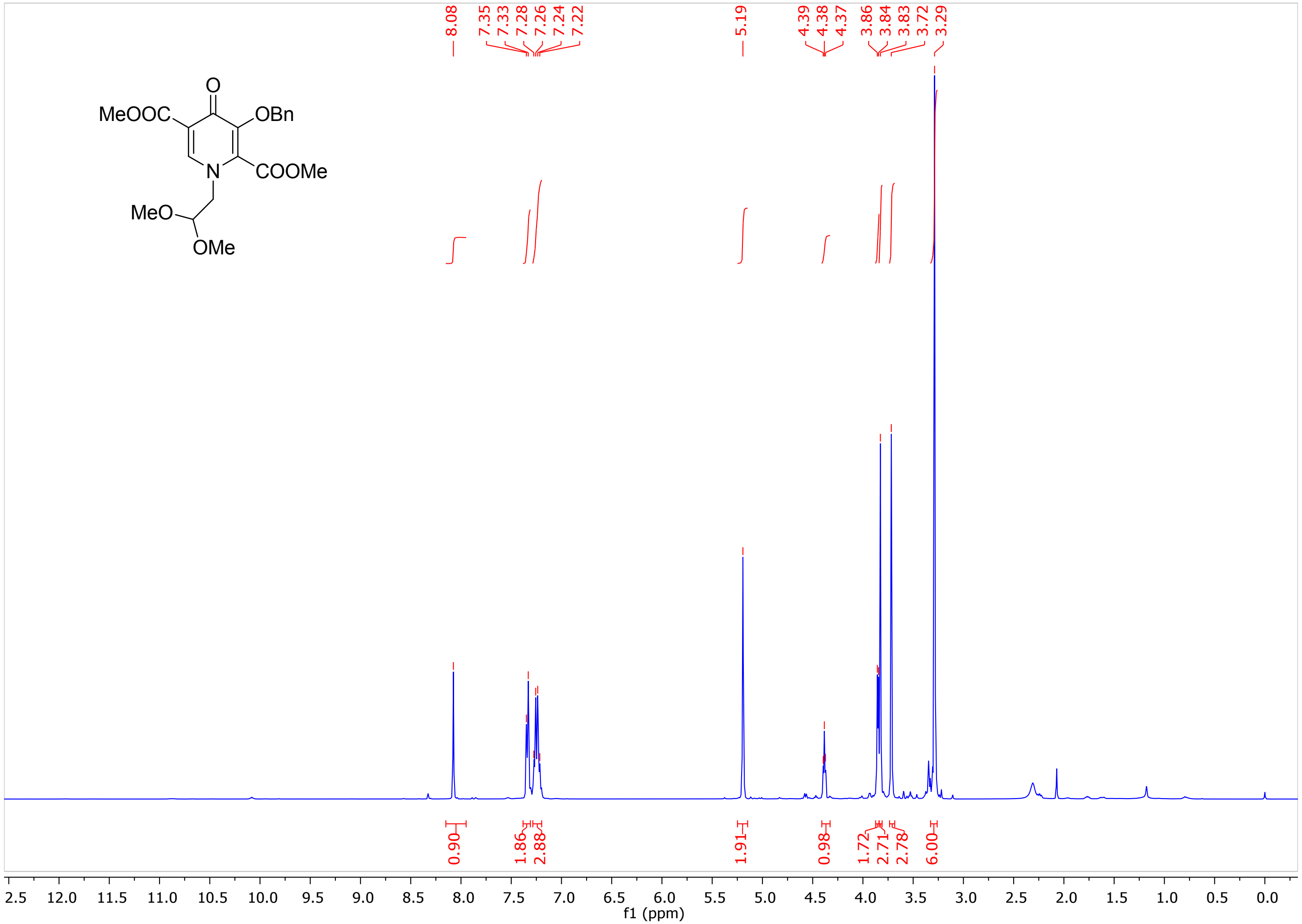

Figure S12: ^{1}H NMR (400 MHz, $CDCl_3$) for pyridinone **3**

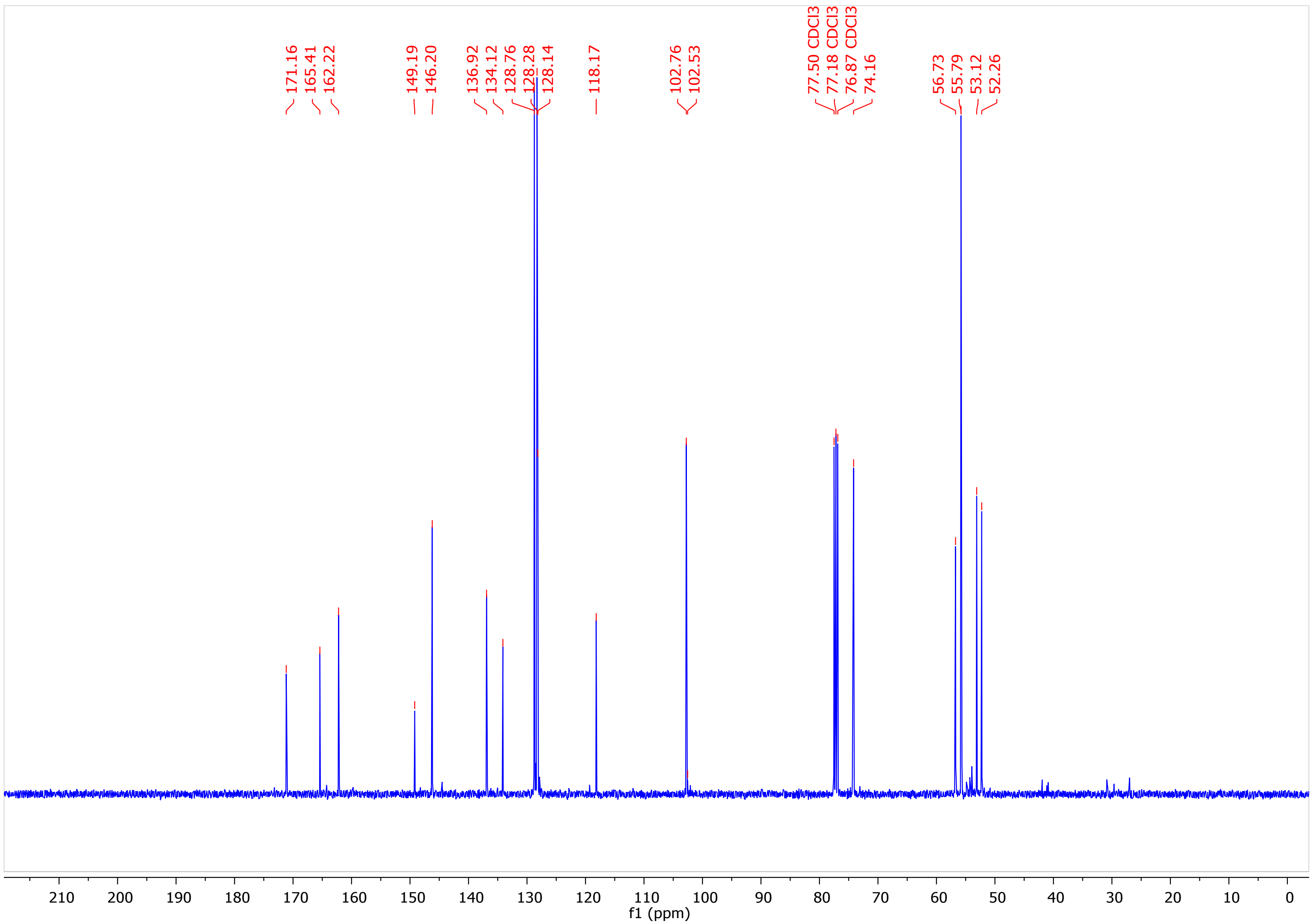

Figure S13: $^{13}C\{^{1}H\}$ NMR (100 MHz, $CDCl_3$) for pyridinone **3**

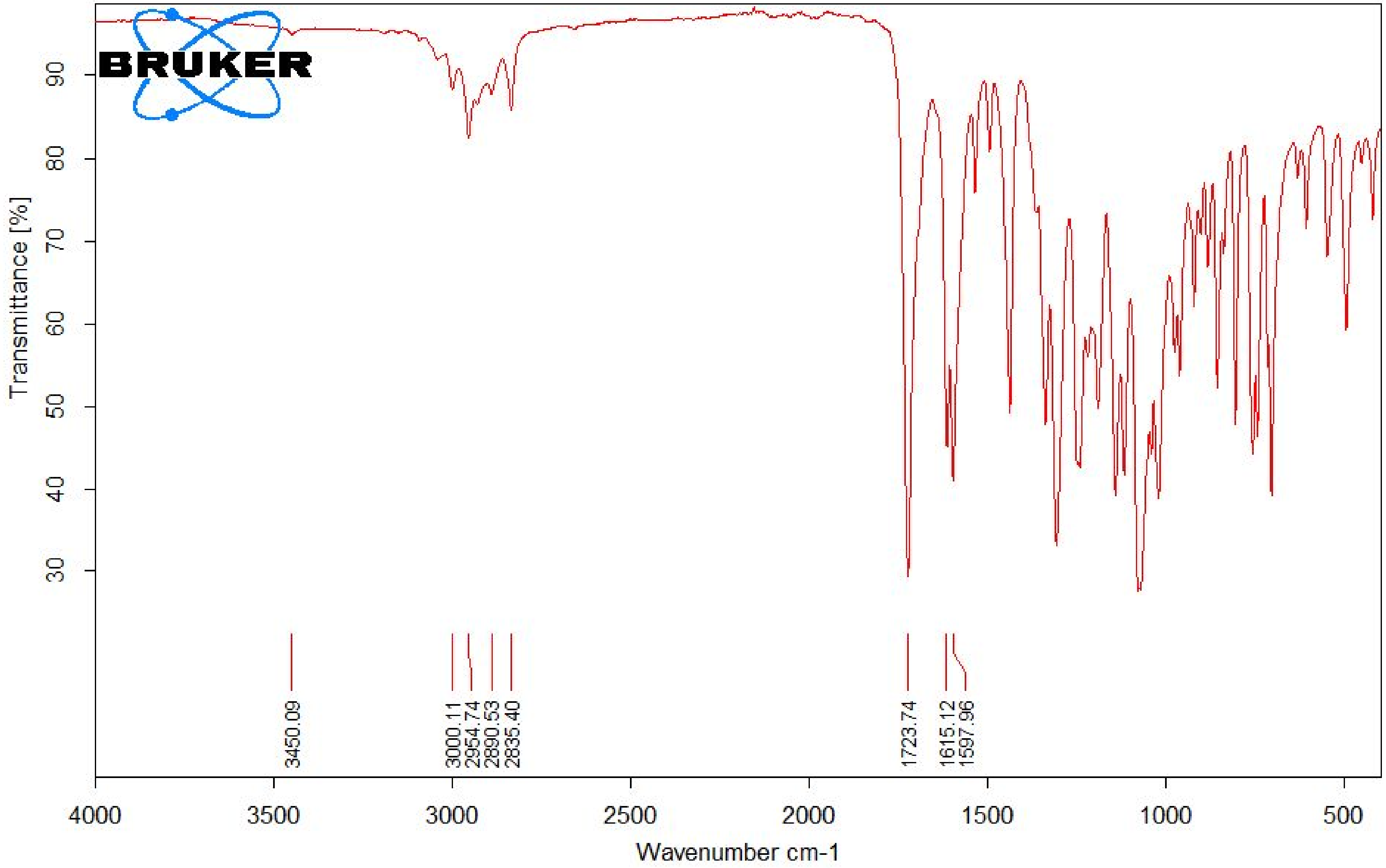

Figure S14: FTIR spectrum of pyridinone **3**

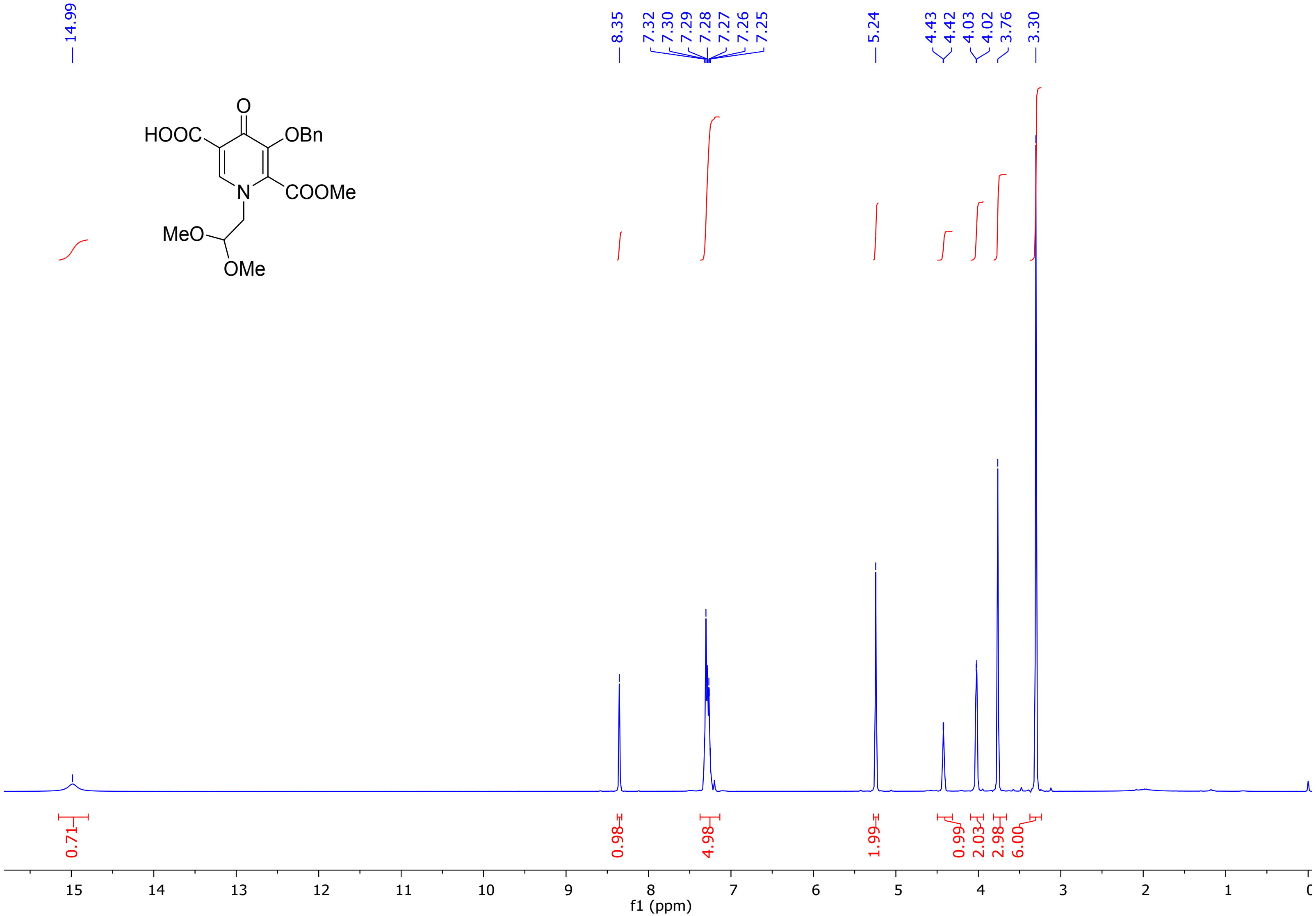

Figure S15: ^{1}H NMR (400 MHz, $CDCl_3$) for carboxylic acid **4**

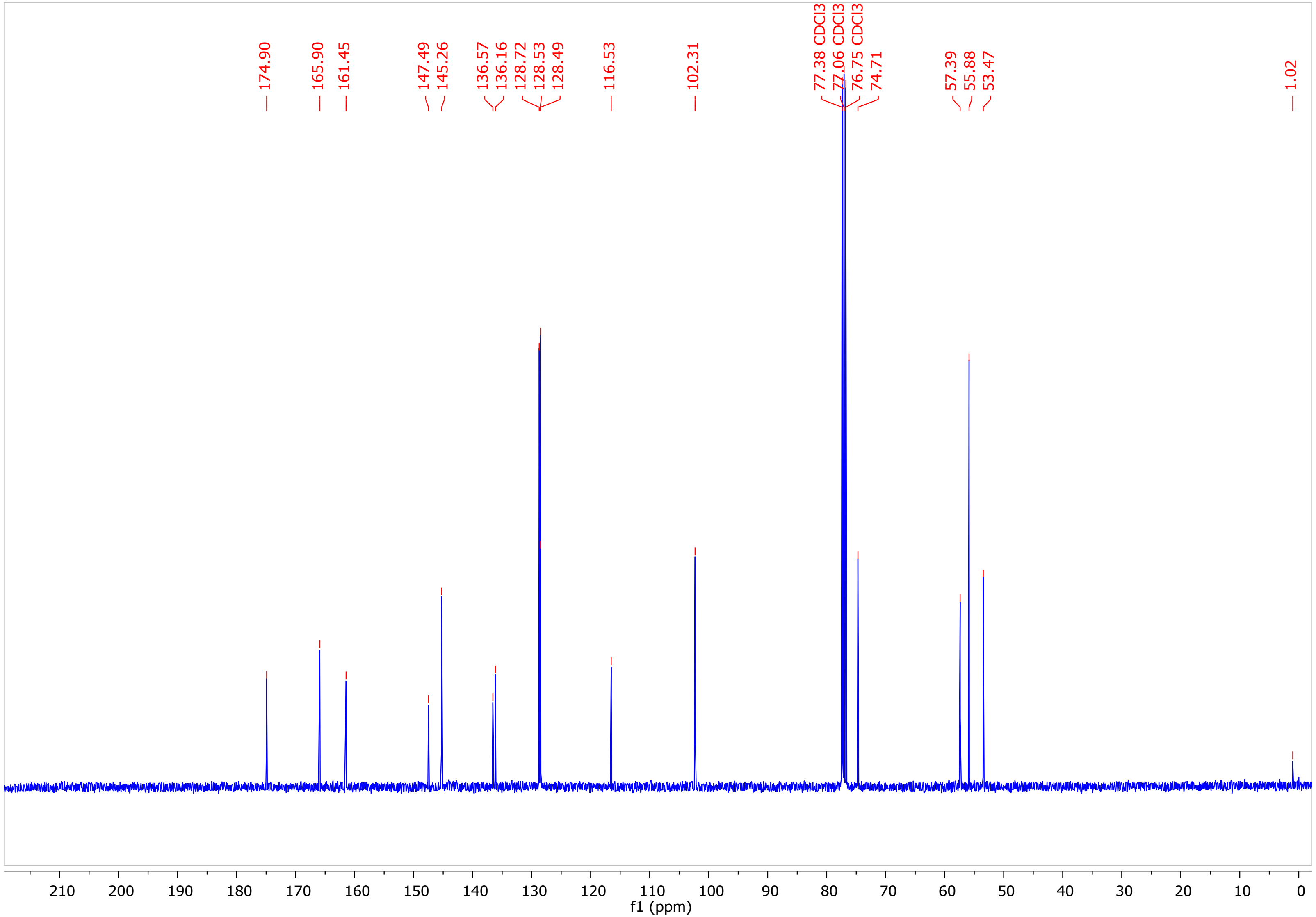

Figure S16: $^{13}C\{^{1}H\}$ NMR (100 MHz, $CDCl_3$) for carboxylic acid **4**

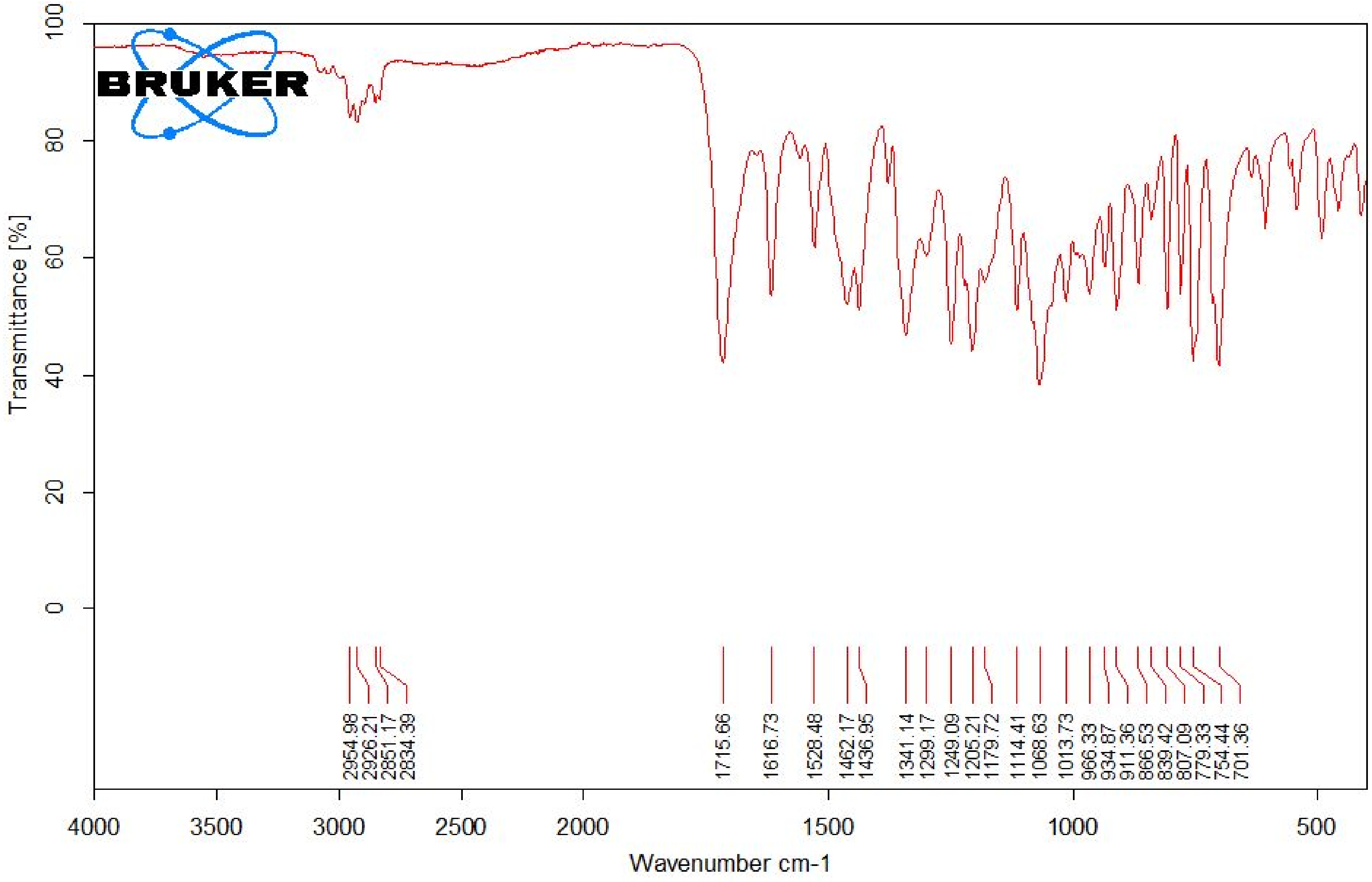

Figure S17: FTIR spectrum of carboxylic acid **4**

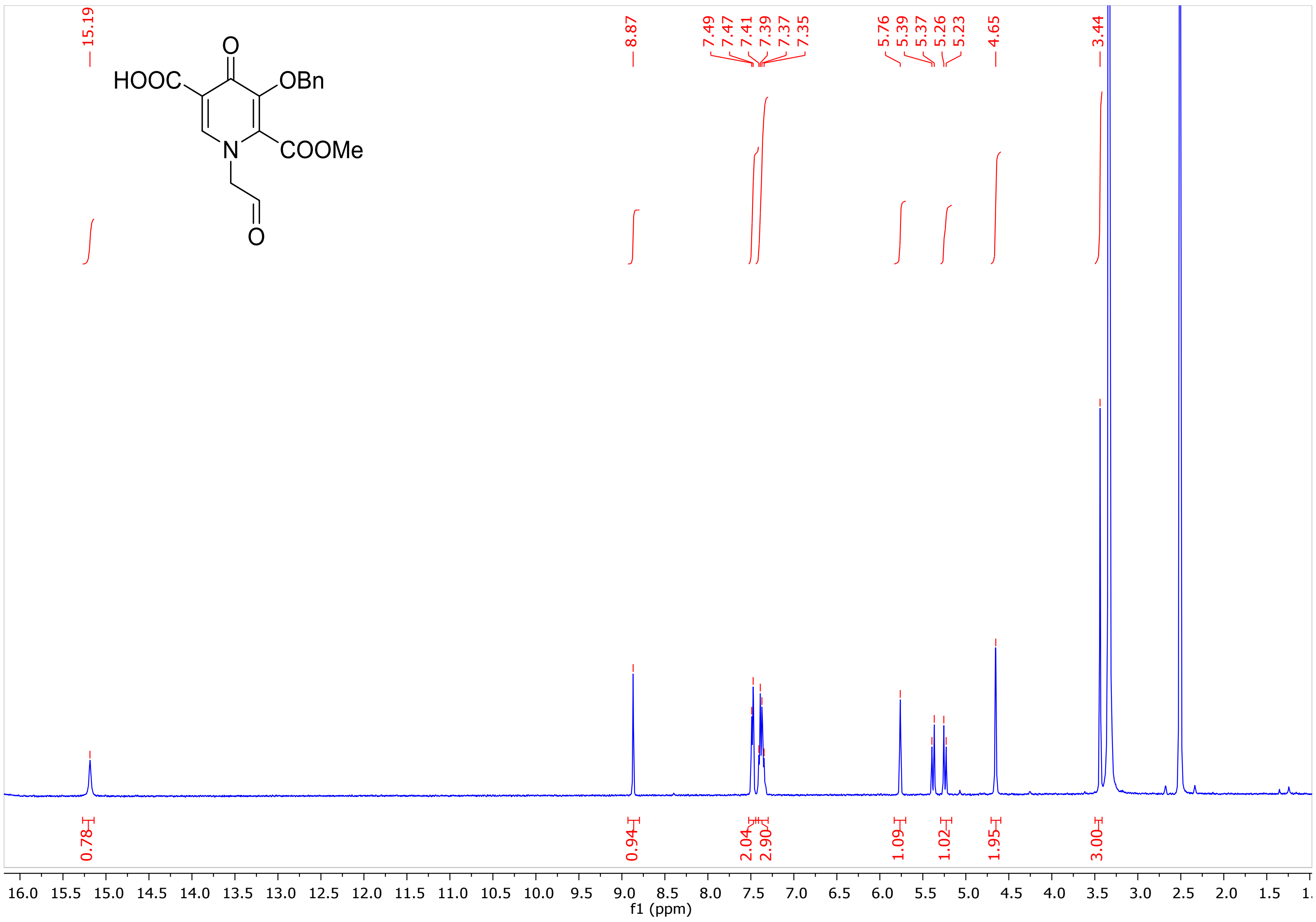

Figure S18: ^{1}H NMR (400 MHz, DMSO-d_6) for Aldehyde **5**

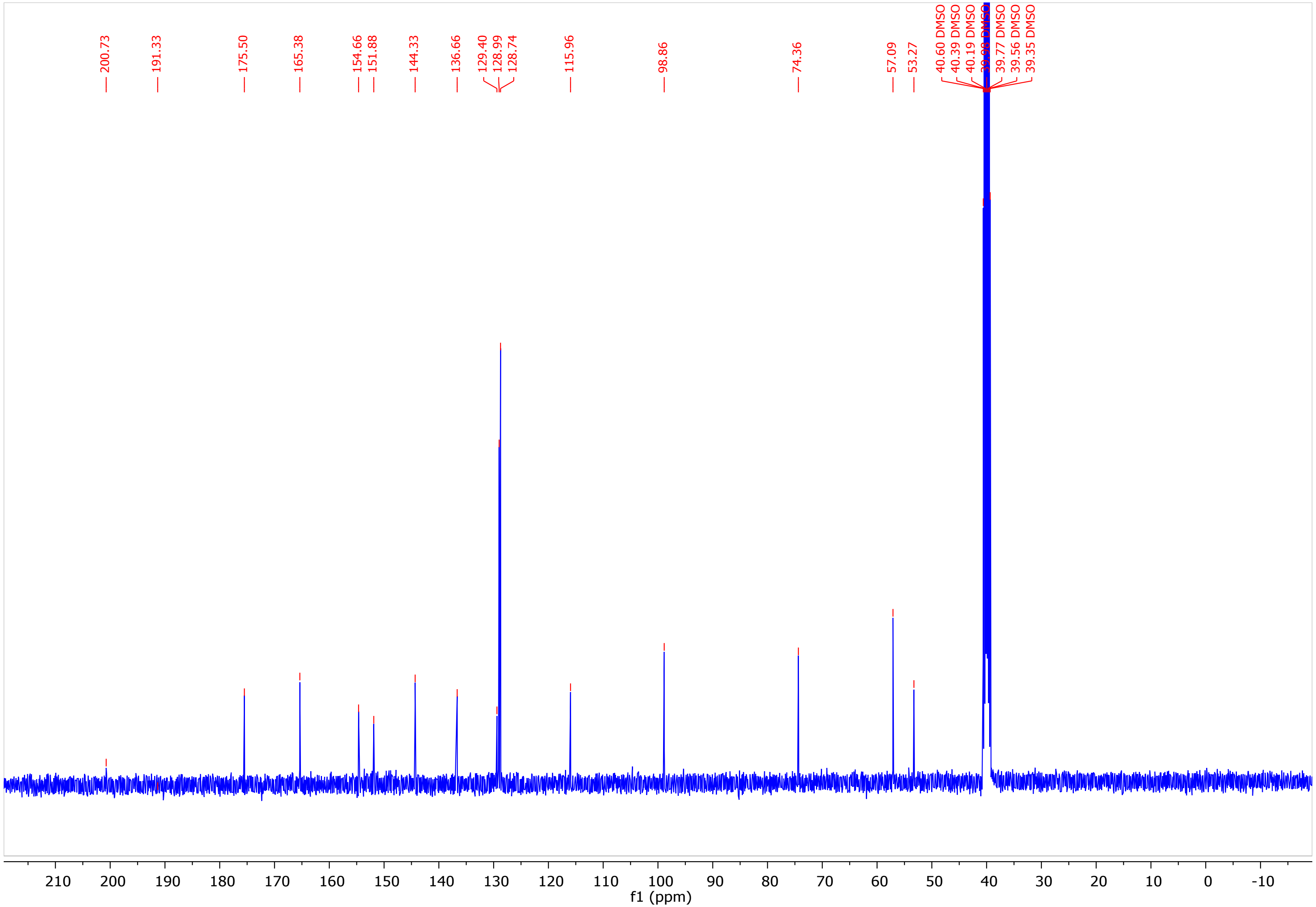

Figure S19: $^{13}C\{^{1}H\}$ NMR (100 MHz, DMSO-d_6) for Aldehyde **5**

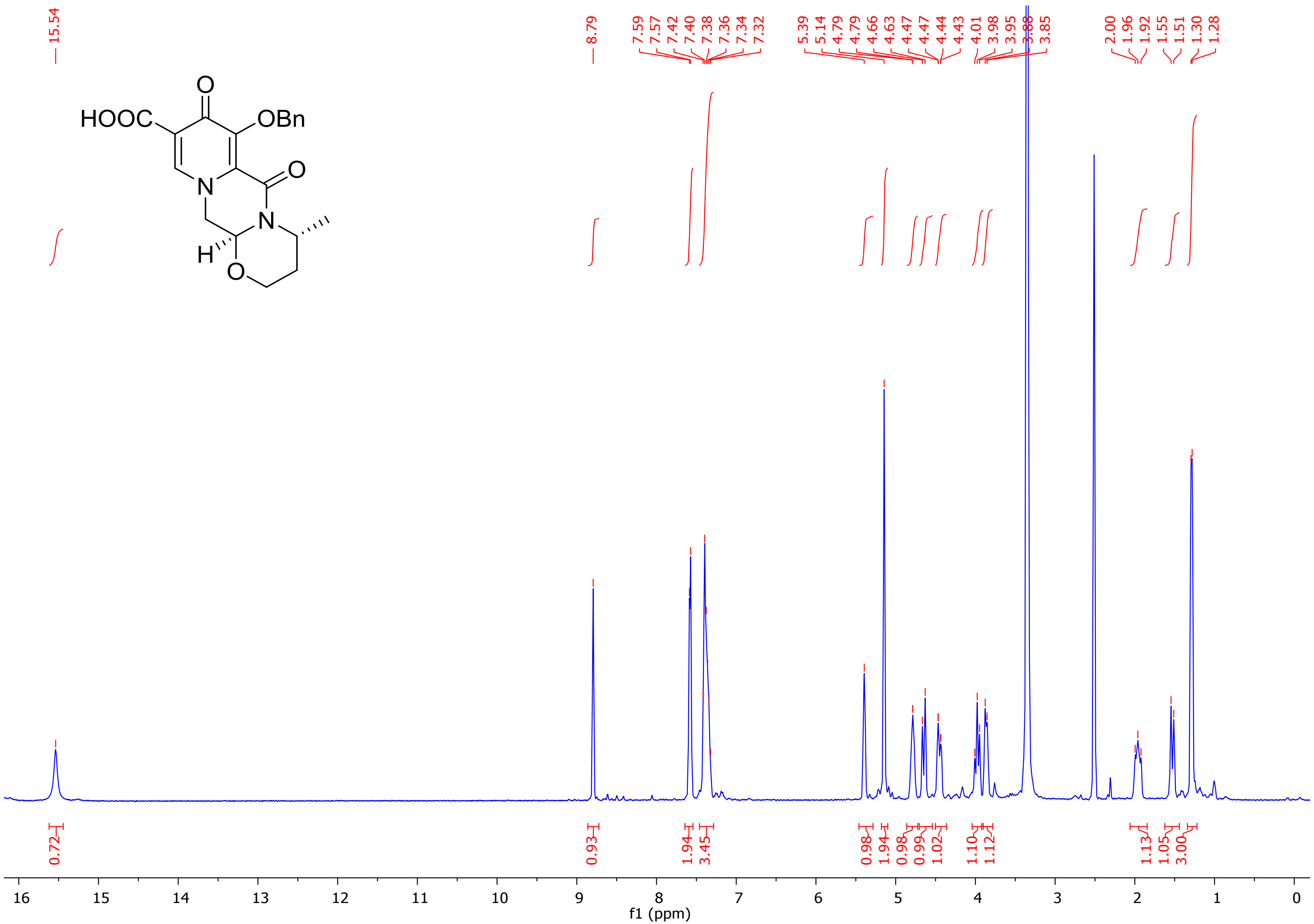

Figure S20: ^{1}H NMR (400 MHz, DMSO-d_6) for Dolutegravir penultimate intermediate **6**

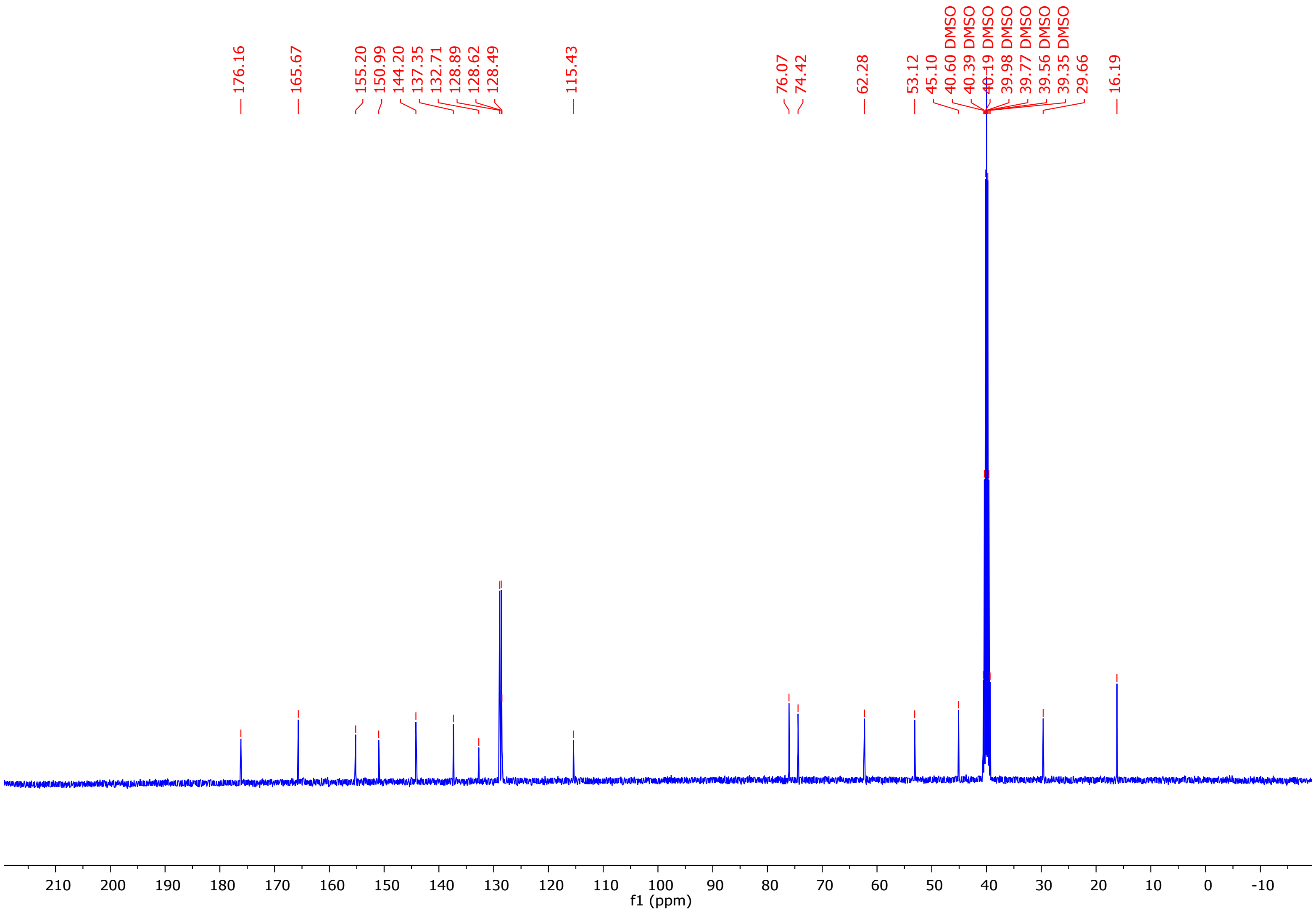

Figure S21: $^{13}C\{^1H\}$ NMR (100 MHz, DMSO-d_6) for Dolutegravir penultimate intermediate **6**

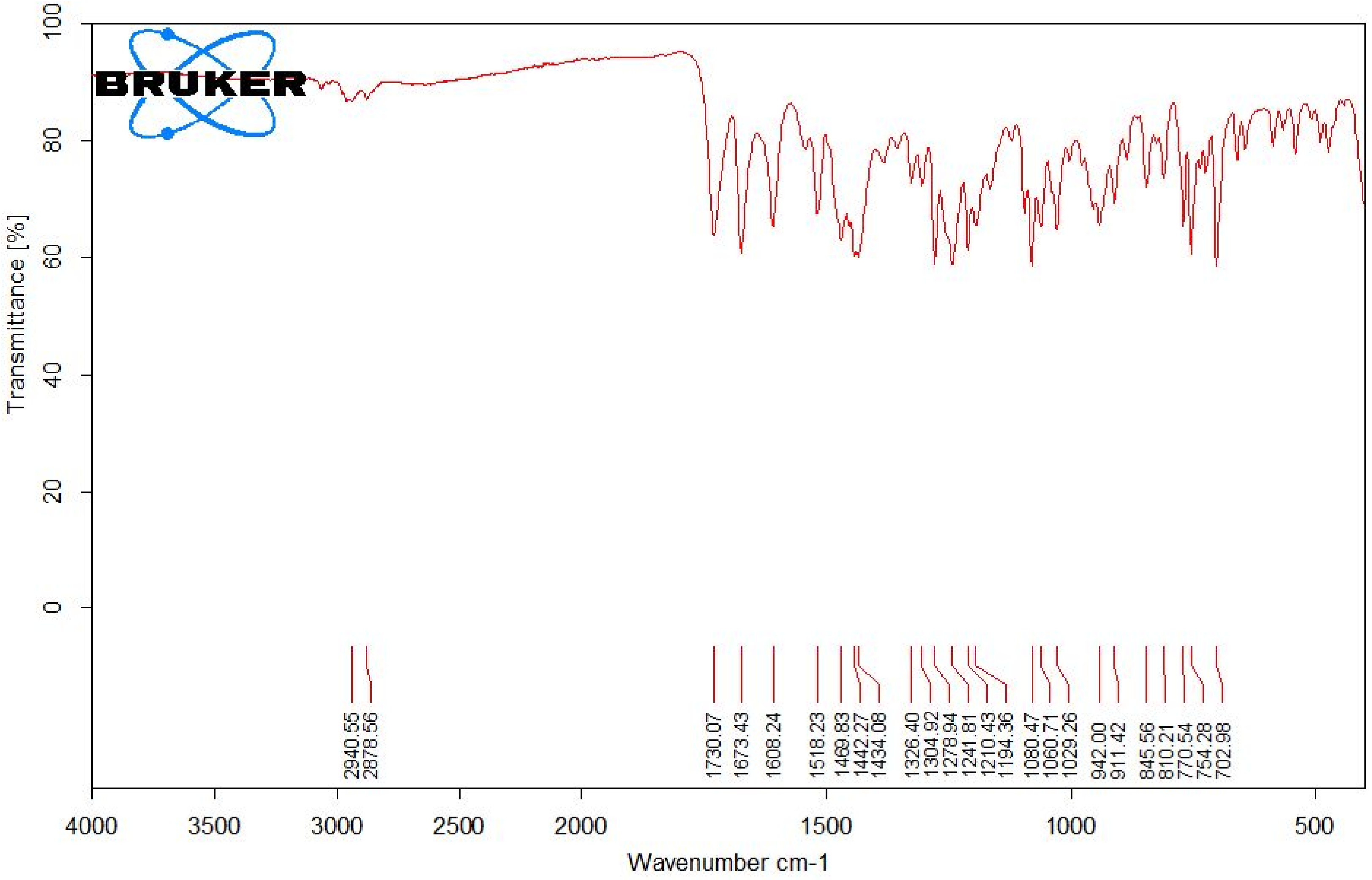

Figure S22: FTIR spectrum of Dolutegravir penultimate intermediate **6**

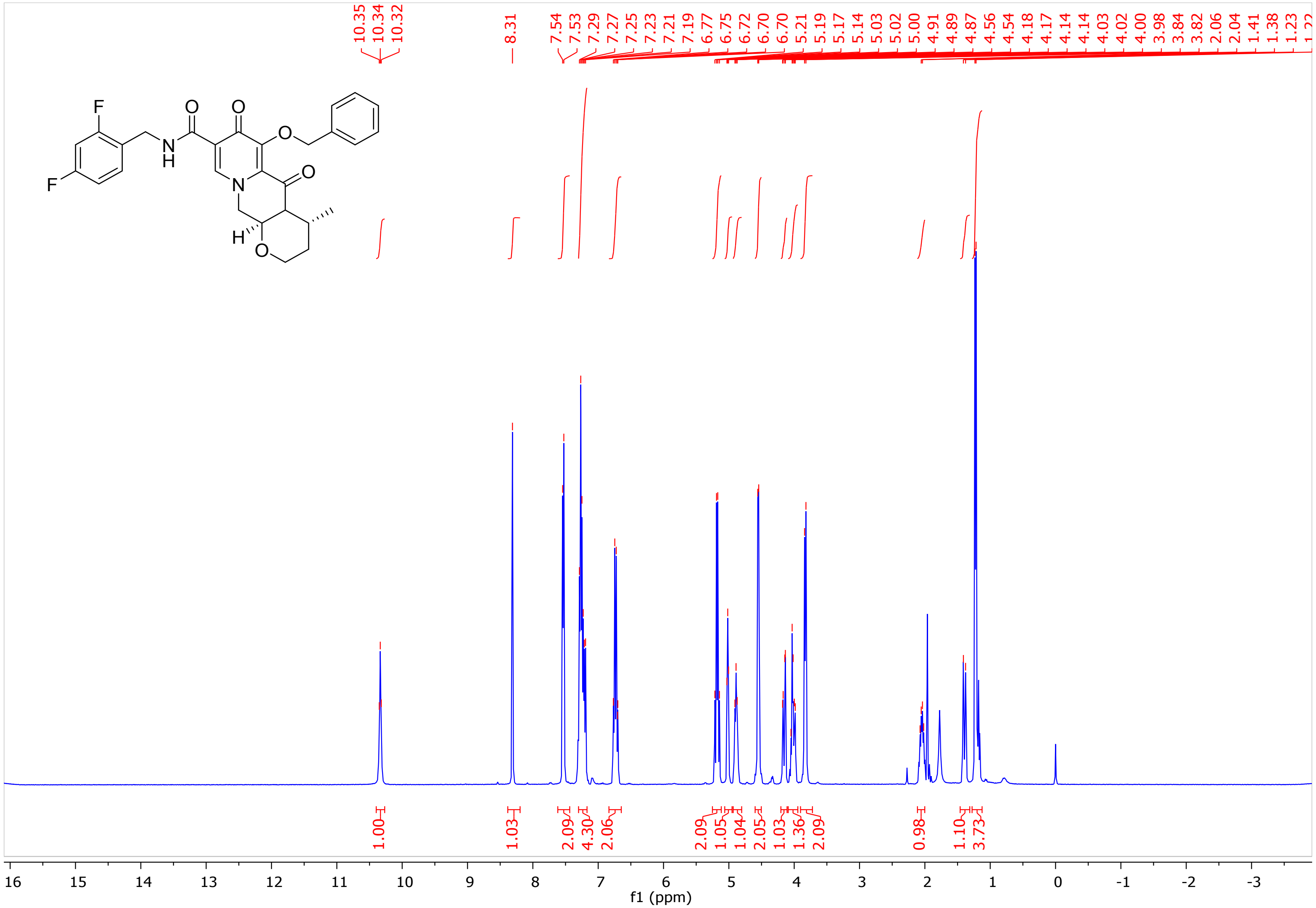

Figure S23: ^{1}H NMR (400 MHz, $CDCl_3$) for benzyl protected dolutegravir intermediate **7**

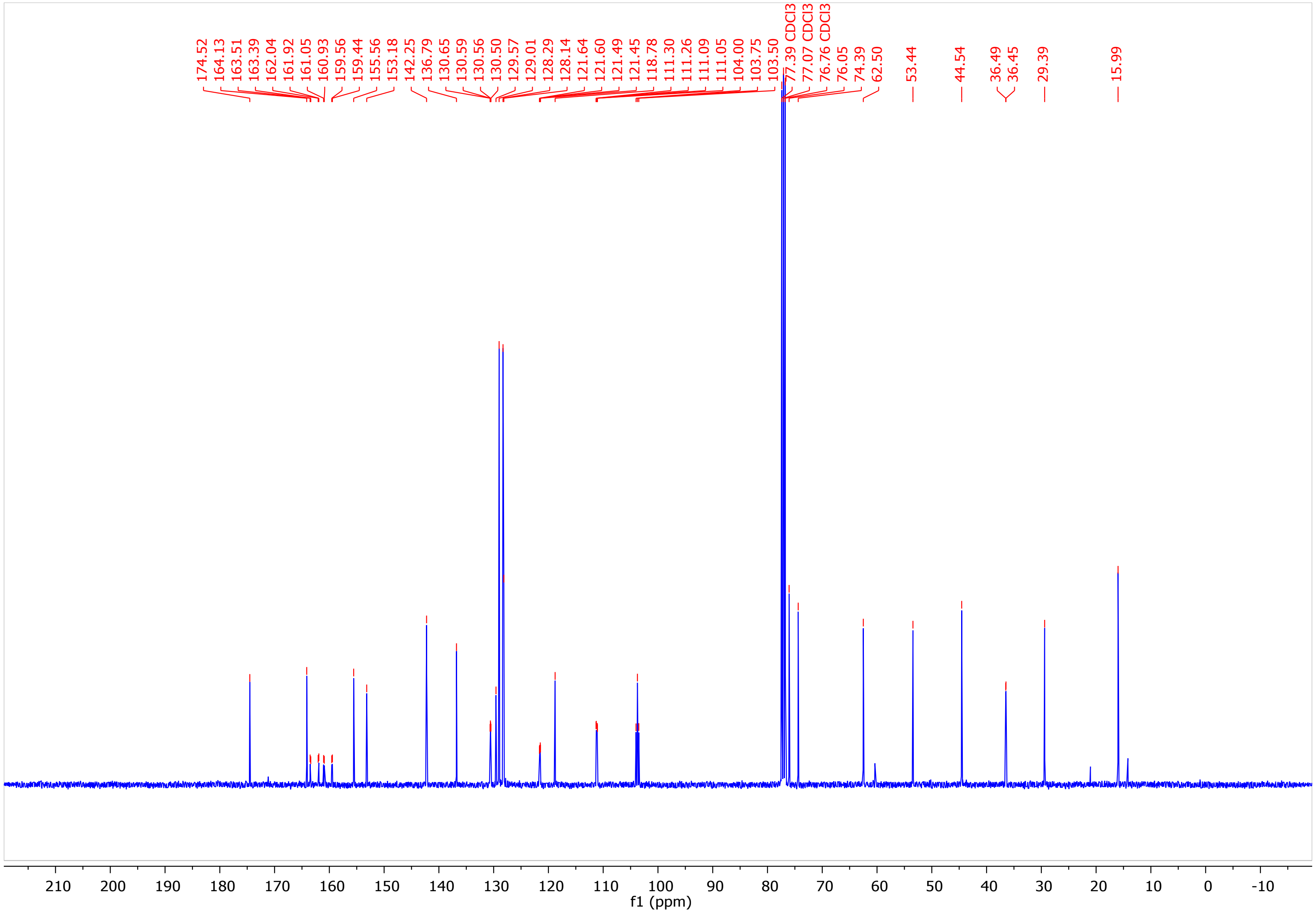

Figure S24: $^{13}C\{^{1}H\}$ NMR (100 MHz, $CDCl_3$) for benzyl protected dolutegravir intermediate **7**

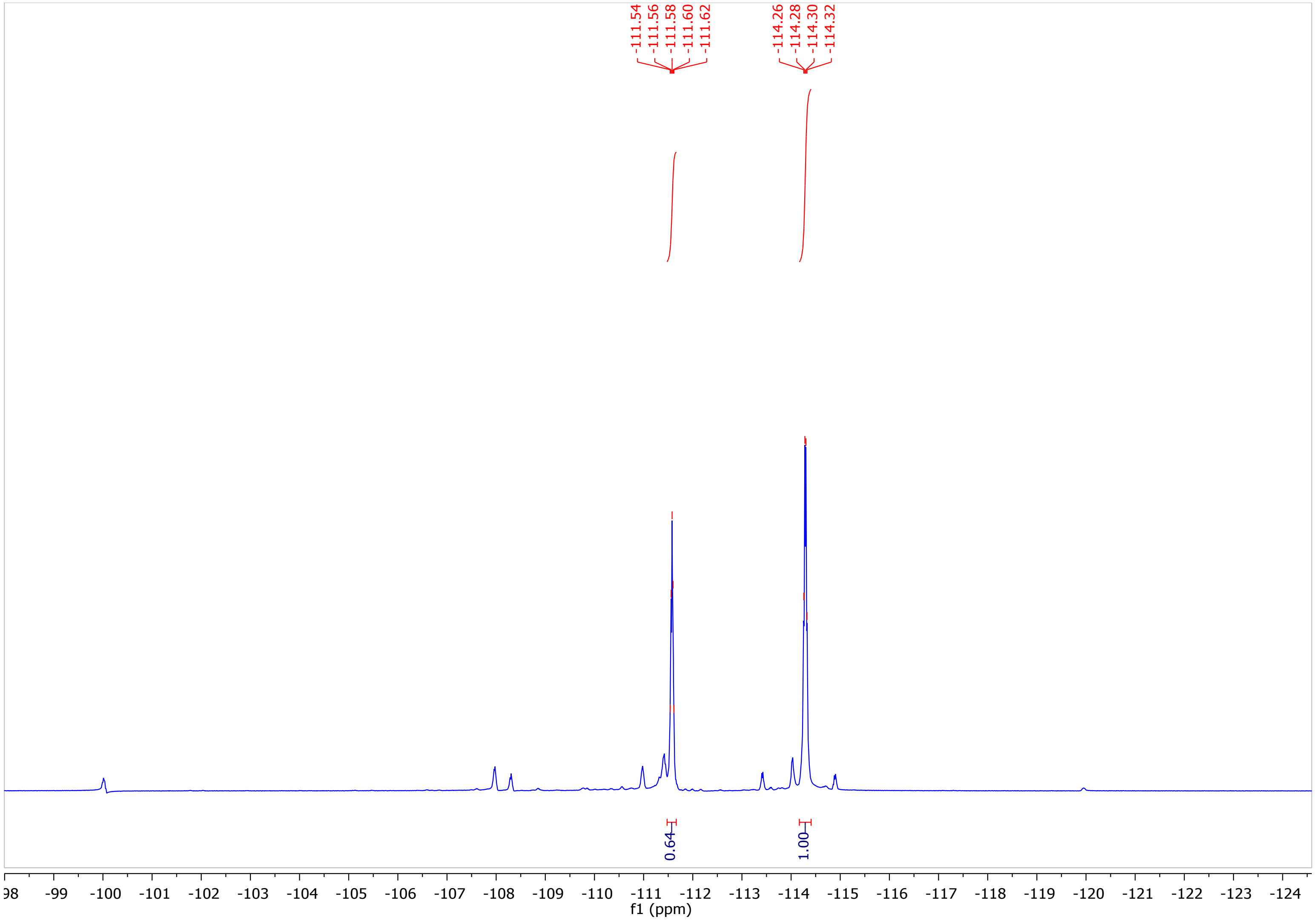

Figure S25: ^{19}F NMR (376 MHz, $CDCl_3$) for benzyl protected dolutegravir intermediate **7**

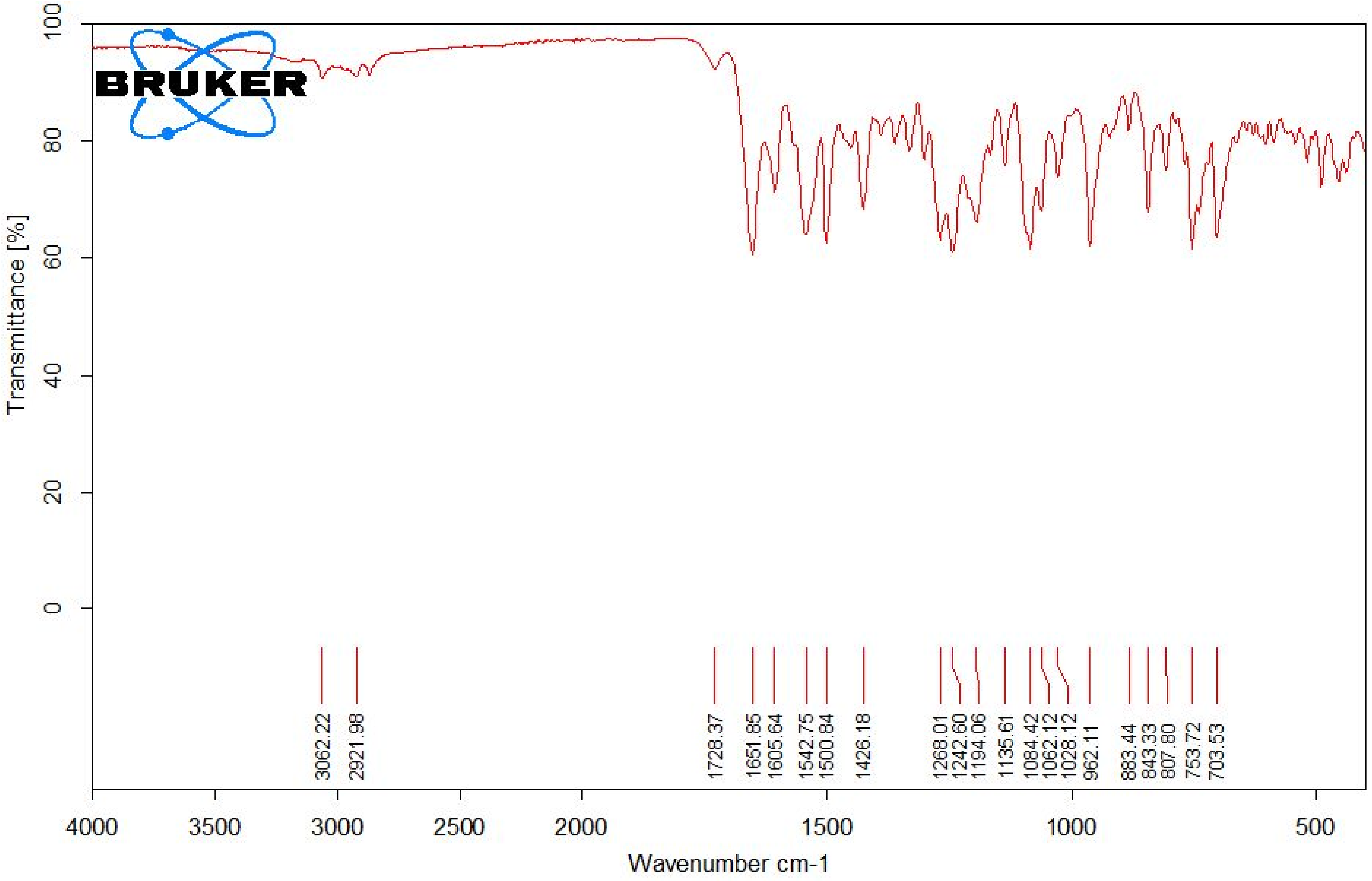

Figure S26: FTIR spectrum of benzyl protected dolutegravir intermediate **7**

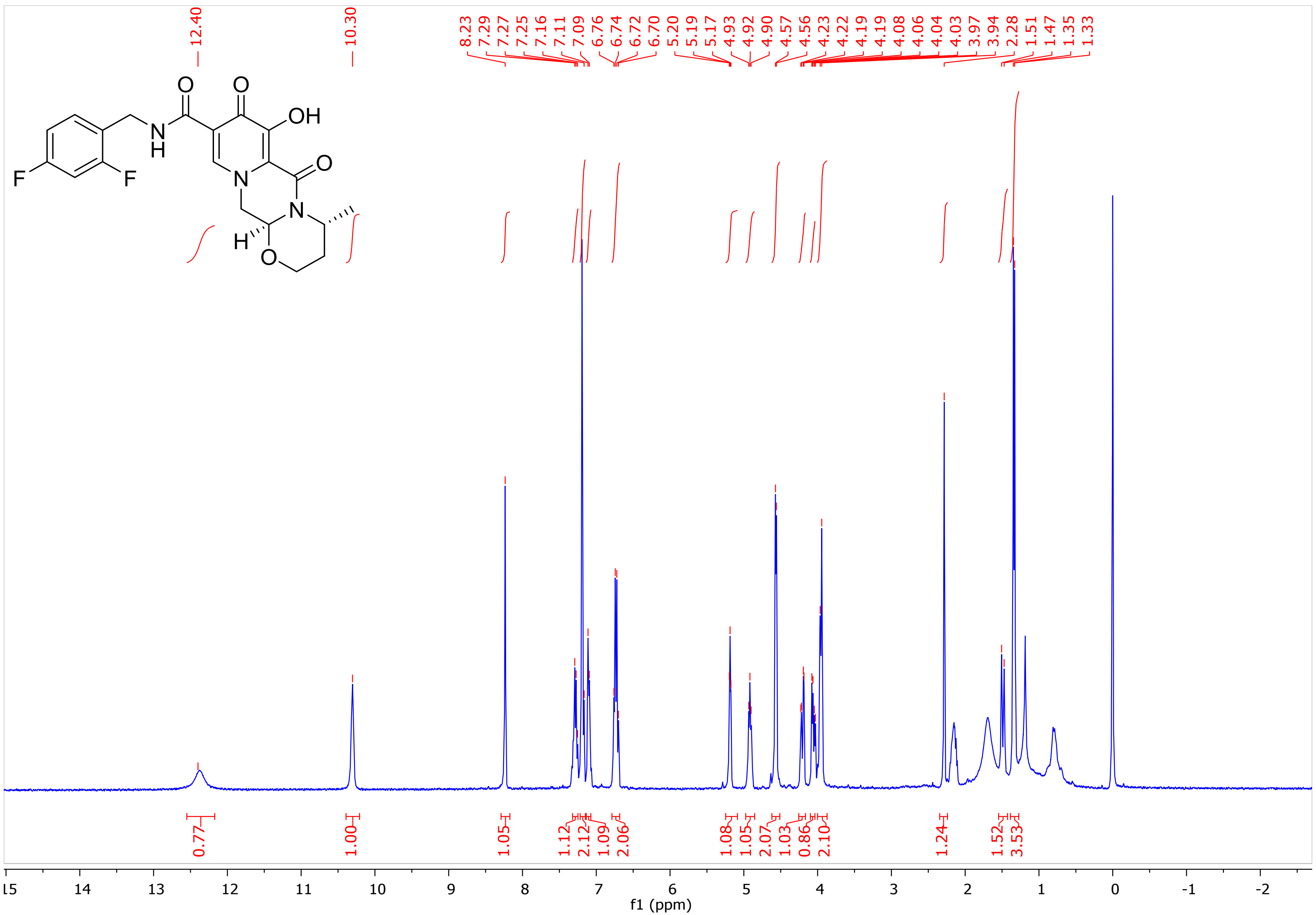

Figure S27: ^{1}H NMR (400 MHz, $CDCl_3$) for dolutegravir free acid **1**

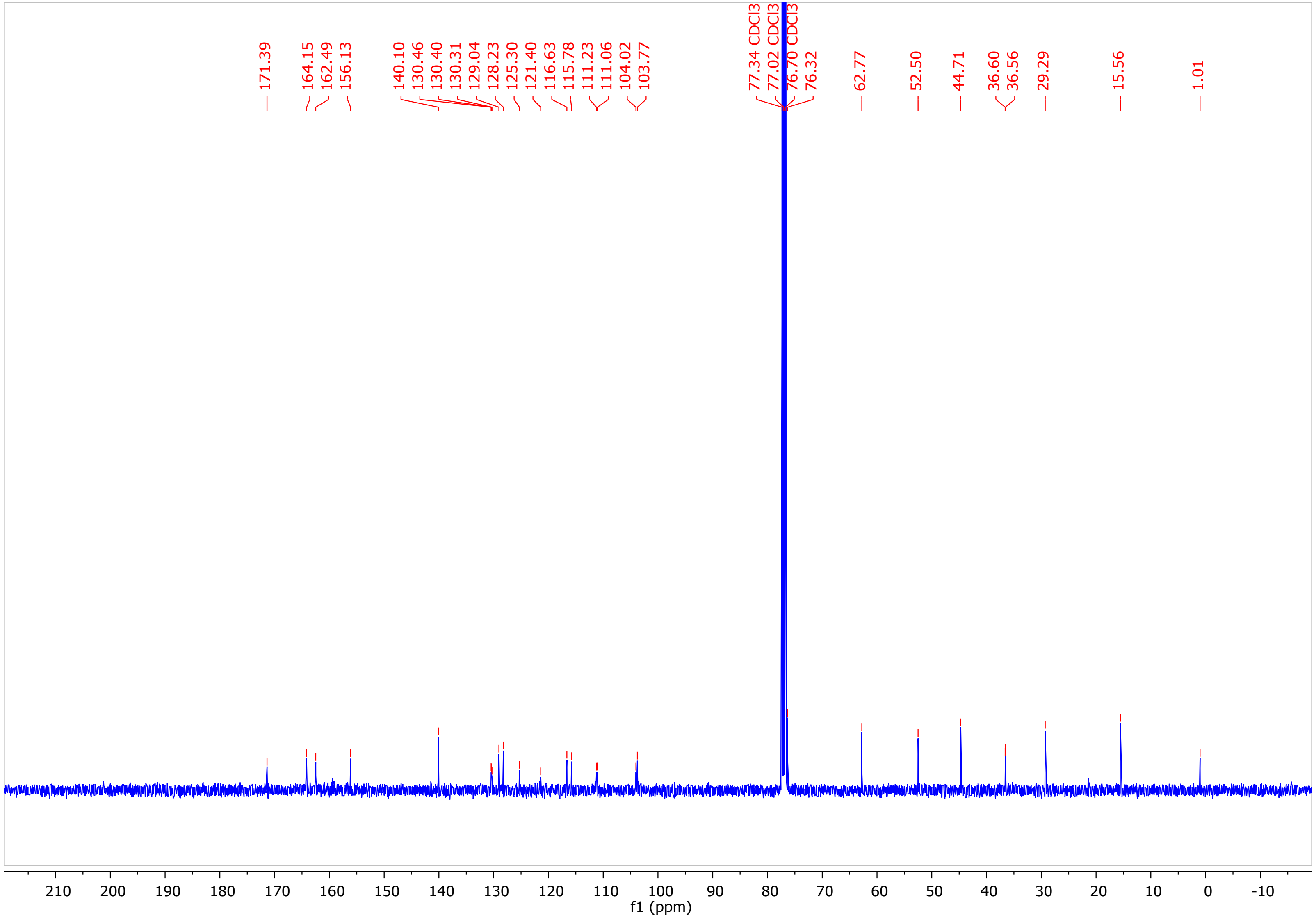

Figure S28: $^{13}C\{^{1}H\}$ NMR (100 MHz, $CDCl_3$) for dolutegravir free acid **1**

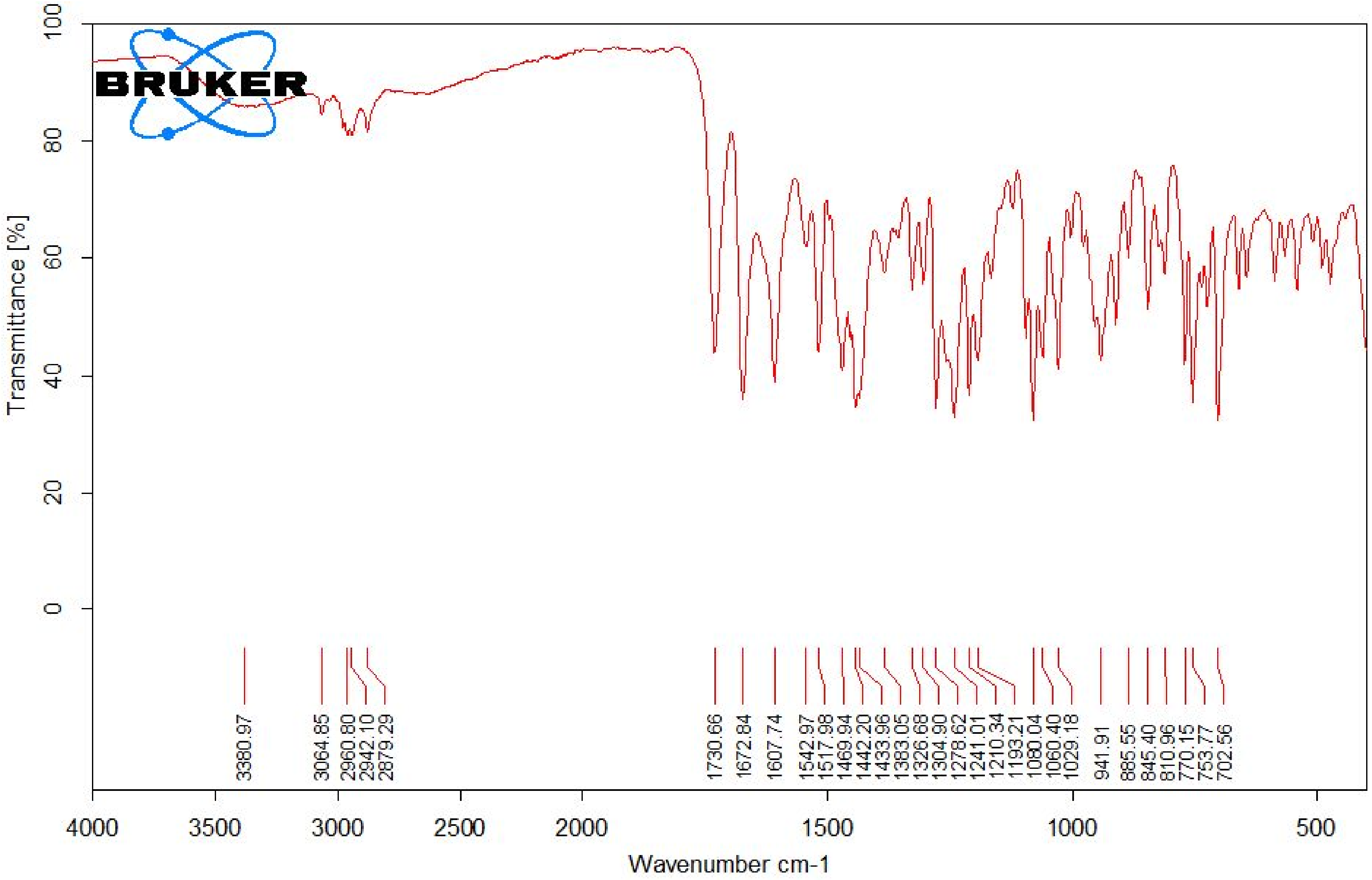

Figure S29: FTIR spectrum of dolutegravir free acid **1**
